# Strengths and Limitations of Period Estimation Methods for Circadian Data

**DOI:** 10.1371/journal.pone.0096462

**Published:** 2014-05-08

**Authors:** Tomasz Zielinski, Anne M. Moore, Eilidh Troup, Karen J. Halliday, Andrew J. Millar

**Affiliations:** 1 SynthSys, University of Edinburgh, Edinburgh, United Kingdom; 2 EPCC, University of Edinburgh, Edinburgh, United Kingdom; University of Texas Southwestern Medical Center, United States of America

## Abstract

A key step in the analysis of circadian data is to make an accurate estimate of the underlying period. There are many different techniques and algorithms for determining period, all with different assumptions and with differing levels of complexity. Choosing which algorithm, which implementation and which measures of accuracy to use can offer many pitfalls, especially for the non-expert. We have developed the **BioDare** system, an online service allowing data-sharing (including public dissemination), data-processing and analysis. Circadian experiments are the main focus of BioDare hence performing period analysis is a major feature of the system. Six methods have been incorporated into BioDare: Enright and Lomb-Scargle periodograms, FFT-NLLS, mFourfit, MESA and Spectrum Resampling. Here we review those six techniques, explain the principles behind each algorithm and evaluate their performance. In order to quantify the methods' accuracy, we examine the algorithms against artificial mathematical test signals and model-generated mRNA data. Our re-implementation of each method in Java allows meaningful comparisons of the computational complexity and computing time associated with each algorithm. Finally, we provide guidelines on which algorithms are most appropriate for which data types, and recommendations on experimental design to extract optimal data for analysis.

## Introduction

Circadian biology has been studied since the 18^th^ Century and has been an area of increasingly active research across an ever wider range of organisms since the 1950's. Circadian clocks have now been identified across the whole Tree of Life in organisms ranging from cyanobacteria [1], [2] through to mammals [3], [4]. In order to improve understanding of the various clock mechanisms and of their significance, models of the circadian clock have been developed for many organisms. These models vary from simple 3 protein post-translational oscillators, for instance the Kai A, Kai B, Kai C clock of *Synechococcus elongatus* [5] to the more complex integrated feedback and feed-forward loops of the mammalian circadian clock [3], [4]. The various models can be tested through simulation, experimentation or both.

One of the key steps in identifying the molecular components of the various clocks is to examine the rhythmicity, or arhythmicity, of time course data obtained either from simulations or experiments. If the data are found to be rhythmic then it is vital to be able to make an accurate estimate of the underlying period. Key components in the clocks of all species have been discovered by forward genetic approaches, starting from the identification of a single individual with an altered period among a large population (for example [6]). Reverse genetic or drug screens for RNAi probes or chemical compounds that alter circadian properties use very similar, large-scale period assays [7], [8]. There are many different techniques and algorithms for doing the analysis required, all with different assumptions and with differing levels of complexity. Many of the algorithms are available as parts of software packages which can be either proprietary or freely available; some of these will be discussed later. Choosing which algorithm, which implementation and which measures of accuracy to use can offer many pitfalls, especially for the non-expert.

Advances in experimental techniques have facilitated the execution of long, circadian timeseries experiments. This creates new challenges in the form of data processing and data management. **BioDare** (Biological Data Repository) was developed under the multi-site ROBuST project (http://hallidaylab.bio.ed.ac.uk/ROBuST.html) to address such issues, and continued under the TiMet project (http://www.timing-metabolism.eu). BioDare is an online service which allows data-sharing (including public dissemination), data-processing and analysis, with the main focus on time-series data produced in circadian experiments from model species. One of the most important aspects of the data processing capability is its period analysis facility. Rhythmic data analysis was initially performed using the FFT-NLLS algorithm [9]. We have since added five other analysis methods: Enright and Lomb-Scargle periodograms [10], [11], mFourfit [12], MESA [13] and Spectrum Resampling [14], which we introduce below.

Having provided six different analysis methods, several broadly-relevant questions arose, including: which method is the best? Which method should I use for our data? How often do I need to sample my data? Can I analyse only X days of data [where X is a small number]? Here we report a detailed assessment of the selected period estimation algorithms, to address these and related questions. The results have broad relevance, as the algorithms tested represent a spectrum of popular period analysis techniques. The direct motivation was an objective evaluation of the methods available in BioDare. The guidelines are particularly relevant to BioDare users but do not include any BioDare-specific protocols, which are published elsewhere [15].

This paper starts with a brief review of the six methods and some of the alternatives. The work presented here used our own implementations of the six algorithms, which were re-factored into Java. This allowed us not only to make minor adjustments to the code but also allowed meaningful comparisons of the computational complexity and computing time associated with each algorithm.

Many previous studies have measured the performance of algorithms by evaluating them on real data. In such an approach, the underlying period is not known *a priori*. To avoid this problem we generated synthetic data sets that allow us to quantify an algorithm's performance. We focus on stationary data (with constant period), which is the most common analytical approach, although period is not always stable in free-running biological systems [16]. We examined the six methods not only in terms of the accuracy of the period estimate, but also in terms of speed. This latter factor is becoming increasingly important as recent advances in assay technology and computing power have led to an almost exponential growth in the complexity of experiments and the size of the resulting data sets. For example, molecular genetic experiments using reporter genes such as Luciferase can now routinely generate total volumes of data that were once the exclusive preserve of animal activity monitors or electrophysiological readings.

Having assessed the six algorithms under a variety of conditions, we offer some guidelines as to their use, to extract the maximum useful information from experimental data.

### Algorithms

There is a plethora of different techniques available for the analysis of the periodicity of time-series data, e.g. [17], [18], and these techniques can be categorized in a variety of different ways: parametric vs. non-parametric; Fourier-transform based techniques vs. non Fourier-transform based etc. In deciding which algorithms to evaluate we tried to include algorithms from a variety of different categories and took as our starting point several of the key algorithms already used in the analysis of circadian data. In the following sections we describe the chosen algorithms, focusing on the overall concepts behind each method rather than mathematical/technical details, which can be found in the original papers.

Enright developed conceptually the simplest method for analysis of rhythmic biological data, referred to as the **Enright Periodogram** [10]. Periodic data of known period could be split into sections with the length of the sections matching the underlying period. Each section should contain similar portions of data, as the rhythmic data must contain a repeating pattern. Overlaying the sections will produce a clear waveform (with peak and trough), in which the trough time-points coincide and give a low sum across sections, peak time-points coincide and give a large sum, and the resulting waveform will have large amplitude. However, if the data were split in sections where the length does not correspond to the underlying period, then the peaks and troughs will not coincide and summing the sections together will result in a small-amplitude signal. This observation lies at the heart of the method. To analyse data with unknown period, the algorithm steps through a series of test period values, for each of them performs the procedure described above, and selects the period that gave the averaged waveform of the highest amplitude. To test the statistical significance of the period, Sokolove and Bushell [19] modified the calculation of the amplitude of the resulting waveform. This method, known as the Chi-square Periodogram, was implemented in BioDare and is here referred to as EPR.

EPR has the advantage of being intuitively straightforward and computationally simple. Its main limitation is that the step size between periods that can effectively be tested is constrained by the duration and sampling frequency of the input data.

Another general approach to period estimation is based on the idea of curve fitting. If the measured time series can be represented by a function (curve) of known period, the period of the data can be assumed to be equal to the period of that function. The challenge lies in finding such a function. Typically a model-based approach is used, i.e. a function is chosen that depends on parameters that determine not only its period but also its shape. In a ‘naive’ approach, all possible combinations of the function's parameters can be tested; for each combination, the function's time-series values can be calculated. The set of parameters which gives a time-series closest to the original data is chosen. In general, there is an untenably large number of parameter combinations, so such a naive approach is not feasible. Fortunately, there are known mathematical techniques to find optimal parameters, for example linear- and non-linear least-squares fitting [20]. Many methods of period analysis adopt this scheme, though they differ in the model functions and the selection procedures.

**mFourfit** [12] is one of the curve-fitting methods. It was developed for use with data obtained under entrained conditions, where the phase of entrainment was of particular interest. Stable entrainment implies that the underlying signal will have a single period, namely the period of the entraining cycle *T*. The waveform may be complicated but it is assumed to be the same in each entrained cycle (like the sections into which EPR splits data).

mFourfit's model function consists of a main cosine component of phase φ_1_, amplitude A_1_ and period τ. Up to 4 additional cosine components may be included, each with its own phase and amplitude but with a period that is a simple fraction of the main period τ, from τ/2 to τ/5:
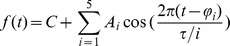
(where A_i_ is the amplitude of each cosine, φ_i_ its phase and *τ/i* its period)

Using the sum of 5 cosines allows for the construction of quite complicated shapes, while the constraint that all components have period τ or a fraction guarantees that the resulting shape has exactly the length of τ. Rather than trying to estimate the period directly, upper and lower boundaries for the period are set by the user and the algorithm then steps through the range of periods in pre-defined increments. For given period τ, mFourfit finds all the parameters for each cosine using ordinary least-squares covariance. This step establishes ‘the best shape’ of length τ that can represent the given data (Note 1). Then, for each period, mFourfit calculates a sum of squared differences between the input data and the theoretical time series generated using the calculated parameters. After iterating through all periods within the boundaries, the period that fits the data with the lowest sum of differences can be determined. This method combines the ‘naive’ approach, checking all potential period values one by one, with selecting model parameters using the least square scheme.

mFourFit also tries to minimise the number of cosine components necessary to reproduce the data shape, in order to reduce the model complexity for each tested period. During period selection, both the fitting error and the model complexity are taken into account, with preference given to periods that yield simpler models.

The main advantage of mFourfit is that it provides the same best-fit waveform for each cycle, which better reflects the underlying biology of an entrained system. The major disadvantage is that the mFourfit algorithm is designed always to return a period (without any significance measure), even if the input time series is arrhythmic. We use the abbreviation MFF to refer to mFourfit method.

Another method that is based on curve fitting is **FFT-NLLS** [9], [21]. This was originally developed at the NSF Centre for Biological Timing in Virginia, to analyse circadian data obtained in free-running conditions (i.e. without entrainment), particularly in genetic screens to identify mutant organisms with altered period. Here, the data are also modelled by a sum of cosine functions, in the form of:
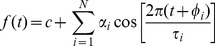
(where: *τ_i_*, *φ*_i_, *α_i_* are period, phase and amplitude of each cosine component, *c* is the offset)

The main differences between FFT-NLLS and MFF are that the periods *τ_i_* of each cosine are independent of each other in FFT-NLLS and the number of cosines *N* can be up to 25. The unconstrained periods and the large number of components mean that almost any curve can be represented by this model. For example, a long period cosine could model a data trend, a mid range cosine would match the ‘main’ oscillation in the data, and very short period cosines could represent sudden changes in the data or even noise. In reality, 5 components are sufficient to model correctly most biological data.

FFT NLLS starts with a model with a single cosine and determines the parameters (*τ_1_*, *φ*_1_, *α_1,_ c*) using a non-linear least squares fitting algorithm. This procedure is repeated using models with additional cosine components (increased N), until adding an additional cosine term does not improve significantly the resulting fit. The precise details of the algorithm can be found in [9]. Once the best model and its parameters have been found, the period is taken to be the period of the cosine component lying within a user-defined range of likely circadian periods (typically 15–35 h). If more than one cosine component belongs to the circadian range, the user has to decide which to select. Conventionally the component associated with the smallest relative amplitude error (defined as the value of the amplitude error estimate divided by the amplitude value) is chosen.

FFT NLLS performs an additional operation, namely finding confidence levels for period, phase and amplitude for all of the cosine components of the best model. This is done by determining the maximum size of perturbation which can be introduced into individual parameters before the resulting fit significantly deviates from the original.

The non-linear least squares procedure that calculates the parameters only works well if sensible initial values are provided. In order to obtain the initial values of the period and phase, a Fast Fourier Transform (FFT) is performed on the input time series [22]. The underlying principle of this common method is explained under MESA, and one of its limitations under Spectrum Resampling, below. Its relevance here is only to learn initial period values from the data, rather than using default or user-defined values. The initial values are then improved by the NLLS iterative numerical search. Hence the full name of this technique: the Fast Fourier Transform Non-Linear Least Squares algorithm, abbreviated NLLS in the Results.

The main advantages of FFT-NLLS are reported to be that the algorithm works well on relatively short and/or noisy data series; it gives confidence levels for period, phase and amplitude; and that the algorithm can identify (and report) arrhythmic data if no period can be identified with sufficiently high confidence. The postulated disadvantage of the algorithm is that it potentially has a limited ability to fit rhythmic data with non-sinusoidal waveforms.

Maximum Entropy Spectral Analysis (**MESA**) [13] uses a completely different approach based on stochastic modelling. The algorithm was championed for use in biological data analysis by Dowse [23] and has been used subsequently in marine biology to investigate swimming rhythms and vertical migration [24], [25].

MESA first fits an autoregressive model to the data. This model assumes that the value at a given time point is the combination of a number of previous values plus some stochastic process (noise): 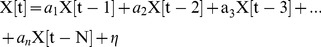
(where: a_i_ are model coefficients, X[t-i] is the data value at previous time point t-i, η is noise, N is the length of the model)

Model coefficients can also be considered as the coefficients of a prediction filter (PF) of length N, where the next value can be predicted using the previous values. Such equations can be written for each data point and filter coefficients that minimise the difference between the predicted and original values can be found using a least-squares approach.

It is possible to obtain a frequency spectrum for the data by using the prediction coefficients. In general, a frequency spectrum characterizes the presence (contribution) of each frequency within the signal, with the most common example being the Fourier Transform power spectrum [22]. Since frequency is the inverse of the period, finding the maximum in a frequency spectrum also identifies the strongest period of the data. Determination of a spectrum and finding its associated peak lie at the foundation of all frequency spectrum-based methods, such as the FFT.

In the MESA approach, the spectrum is constructed using the formula (the scaling constant is removed):
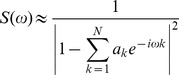
(where a_k_ are the PF coefficients and ω is circular frequency: ω = 2π/τ, τ is period)

The PF length (N) is crucial to the output of the analysis; if the filter length is too low, resolution and important detail can be lost. However, if N is too high, artificial peaks may appear in the spectrum. Although there are procedures to determine the optimal value of the model length, usually manual selection of the minimum value of N is necessary. Once the model length, N, is established, corresponding PF coefficients can be found, the spectrum S is then calculated using the formula above and the period corresponding to the highest value of S is selected.

The main advantages of MESA are that it does not model data assuming any *a priori* shape of waveform, and it has much better precision than Fourier transform based methods. The drawbacks are its dependence on the correct choice of model length and the lack of a significance/confidence measure.

Another spectral analysis technique is the **Lomb-Scargle periodogram** [11]. This method also creates a spectrum representing the significance of each frequency in the analysed data. As before, the period corresponding to the peak is chosen as the output of the method. The very simplified formula for the spectrum is:
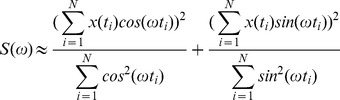
(where: x(t_i_) is the value at time t_i_, N number of data points, ω is the circular frequency: ω = 2π/τ, τ is the period).

It can be instructive to examine this formula to determine why it reflects the presence of a given periodicity in the data. In the first term the measured data are convolved with the cosine function (for each time point, the recorded value in data is multiplied by the corresponding value of a cosine at this time point). Assume a long data series of period T and phase 0, and consider the result of convolving the data with the equivalent cosine, cos(2π/T). Each time the data has its maximum value so does the cosine and the result of repeated multiplication followed by summation will be high, whereas each time the cosine has a negative value the data has its lowest value, and a small value will be subtracted from the sum. Thus the overall sum in the numerator will have a relatively high value. If the same data is convolved with a cosine of period other than T, the cosine no longer peaks at times nT and hence, the elements in the nominator sum will start to cancel each other, leading to a smaller summation than the previous case. The denominator value is a scaling factor which reflects how much ‘value’ the cosine contributed at the given times. The second sine-based term will behave similarly but with rhythmic data of phase T/4. The combination of both cosine and sine terms can measure contributions to the frequency regardless of the data phase (see MFF note 1).

The main advantage of this method is that, unlike most of the spectral methods (for example MESA), it can handle non-evenly spaced data. We refer to this method as LSPR.

**Spectrum resampling** [14], abbreviated as SR in this manuscript, was developed to improve period estimation when the data are non-sinusoidal. The approach uses a power spectrum created by carrying out a Fourier Transform on the time series.

The Fourier Transform and its discrete implementation, the Fast Fourier Transform (FFT), are standards in timeseries processing [22]. Similar to the LSPR, the FFT finds frequency contributions by convolving cosine and sine functions with the analysed signal. However, the spectrum obtained using the FFT cannot be directly used for period estimation of circadian data, due to its poor frequency precision. The precision varies across the spectrum, and is directly correlated with the input data length: to obtain a precision of less than an hour around 24 h periods, there must be over 1000, hourly-sampled time points, i.e. more than one month of measurement. In most cases, biological data must be padded to artificially extend the length of the time series prior to FFT analysis.

The main idea behind spectrum resampling is to find a period “between the cracks” of the original FFT spectrum. The algorithm starts by calculating an ordinary FFT power spectrum. The initial spectrum is smoothed using kernel smoothing. Kernel smoothing can be explained as a more sophisticated form of moving average. Each data point is averaged with scaled values of its neighbours. The kernel method ensures that more distant neighbours contribute less to the average.

The smoothed spectrum forms the basis for the algorithm. Noise is added to the base spectrum, this creates a new sample spectrum, which is successively smoothed, and the frequency value corresponding to the maximum peak in the smoothed spectrum is recorded. This procedure of adding noise to the base spectrum, smoothing, and recording the peak is repeated 1000 times (a process known as boot-strapping).

The recorded peak frequencies are averaged and the mean value is converted to the corresponding period and reported as the data period. For example, if the data has a true period of 24.5 hours, but the precision in the FFT spectrum is limited to about 1 hour around this period due to the data length, each bootstrap iteration could produce period values of 23, 24, 25 or 26 h. The average bootstrap period can be 24.5 (for example 500 peaks at 24 and 500 at 25). The distribution of period values recorded during the bootstrap iterations provides a confidence interval for the period estimates.

The main advantage reported for this algorithm is that it was designed to be more robust to noise and non-sinusoidal time series. The disadvantage is that, because it uses boot-strapping, it is very computationally intensive.

These six methods represent a broad range of the many published approaches to period estimation. EPR is distinct; LSPR, MESA and SR represent spectrum-based methods, while MFF and NLLS are representative examples of curve-fitting methods (for example, the popular Halberg's cosinor procedure [26] is equivalent to MFF with only one cosine). Autocorrelation has not been considered as MESA has been recommended as its replacement, especially for the short data series investigated here.

We omitted two classes of methods that deserve a longer comment: wavelet-based and Bayesian methods. Simple wavelet methods use wavelet transforms as low/high pass filters, which smooth the data and remove trends: such signal pre-processing is not our focus here. The processed data are then analysed with a standard method, for example a simple peak finding algorithm [27]. Alternatively, a continuous wavelet transform may be performed and then changes of period over time can be extracted from the transformation results [28]. Those methods are well suited for non-stationary periods which, although common in biological systems, are not our current focus. Taking into account the lack of evidence that wavelet methods are superior in analysis of stationary periods and the poor support for wavelets currently available in Java, we judged that at this stage, the extra effort necessary to implement such methods for BioDare was not proportional to the potential gain.

Bayesian methods are attractive as they provide well defined confidence levels for period estimates [29], [30]. However, due to their computational complexity we deemed them unsuitable for general use in BioDare. If we consider the typical number of sampling iterations performed by the algorithms (typically above 10,000), we would expect Bayesian methods to be 100 times slower than SR, already the slowest algorithm considered. Furthermore, the documentation of the published algorithms did not allow easy re-implementation.

### Implementations

Hand in hand with the choice of algorithm goes the choice of implementation. This paper uses our implementations of the six algorithms in Java, which have been incorporated into the online BioDare repository. As mentioned briefly in the introduction, period analysis is only one of many BioDare features. The main features of BioDare are:

an online repository for experimental data accompanied by extensive metadata including details about environmental conditions and biological material used
rhythm analysis and period estimation using the mentioned algorithms
generation of secondary data (normalized, detrended, averaged …)
graphical output of data, secondary data and rhythm analysis
simple text-based search throughout metadata
search for data based on biological sample, assay and conditions
data aggregation and export
group-based privacy settings for collaborative research followed by public dissemination.

BioDare was designed to offer flexibility and adaptability to new techniques, data types, experimental designs and use cases. Thus all experimental metadata and system data is stored in XML documents, allowing easy conversion between formats using XSLT. Furthermore, as XML is human readable it speeds up debugging and testing. Java was chosen as the programming language for its self-documenting character, simple error tracing and large number of supported standards and technologies.

The user enters the experiment description in Pedro (a tool that can generate forms which are then populated using the information required by XML document definition) and exports them to XML format. The XML metadata are transformed with XLST to BioDare internal representation and mapped to Java objects for further manipulations using JAXB. The numerical time series are read from Excel files using the Apache POI library. Subsets of the metadata and the time series are stored in a MySQL database using JPA for Java to DB mapping.

Period analysis is controlled by separate subsystem called JobCenter, which allows simultaneous analysis submission by multiple users for multiple data sets. Its main functions are: queuing and housekeeping of the submitted jobs, dispatching analysis to the correct implementation (which can be local or remote using a WebService interface) and sending back completed results. In order to increase the overall performance, JobCenter takes advantage of multiprocessor servers and processes time series in parallel (in the current set-up, 4 time series are analysed in parallel).

BioDare can be found at http://www.biodare.ed.ac.uk, and its source code can be accessed from http://sourceforge.net/projects/biodare. Publicly-accessible data are available to browse and download using the “public” account, while the “demo” account allows users to test the analysis methods.

There are also alternative software packages that offer access to period analysis methods (typically EPR, LSPR, and FFT-based), some of which are listed here. **Clocklab** is a commercial package produced by Actimetrics (www.actimetrics.com/ClockLab/ *),* **Circadian Rhythm** software is a non-commercial suite of programs developed by Refinetti (http://www.circadian.org/softwar.html), **Circwave** and **Chronoshop** are available at (http://webpage2.woelmuis.nl/downloads.htm). MFF and NLLS are provided in **BRASS** developed by Paul E. Brown with the Millar group [31], [32], which is available from (www.amillar.org) but is superseded by BioDare for almost all applications.

## Materials and Methods

In order to evaluate the performance of any period estimation algorithms it is, of course, necessary to know the period *a priori*. This means that it is not practical to use real biological data for any initial algorithm evaluation. Thus different artificial data sets were generated and used to compare the different algorithms. In all cases the time series were stationary so that the underlying period is constant.

The first group of data sets comprised so-called mathematical test signals which, whilst not biologically meaningful, allow the algorithms to be tested against artefacts such as sudden changes in amplitude.

**Figure 1:**
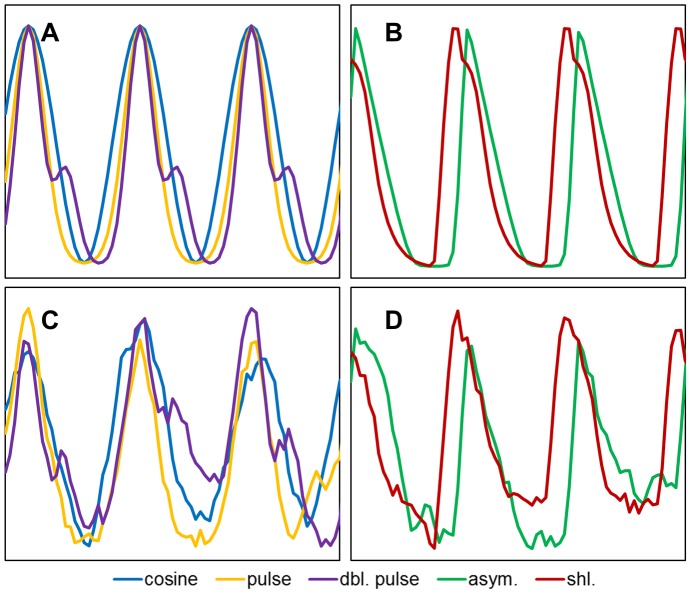
Examples of artificial time series used in the analysis. A) the three mathematical test signals: cosine, pulse and double pulse (dbl. pulse). B) the two model-derived (DNFL) time series: moderate shoulder (shl.) and moderate asymmetry (asym.). C, D) the same test signals with 80% walking noise added.

The mathematical test signals comprise:

- a pure cosine of known frequency, and hence known period;
- a pulsed waveform which comprises pulses of a Gaussian waveform with a standard deviation of (period/7);
- a double pulsed waveform which comprises two periodic Gaussian waveforms, the first is as in the single pulse waveform and the second is a Gaussian waveform of a quarter of the amplitude of the first Gaussian waveform, shifted by period/3 relative to the original Gaussian and with a narrower standard deviation of (period/9);

The second group of time series were designed to be more representative of biological systems whilst still allowing exact knowledge of the underlying period. This group comprised simulated clock data generated using a delayed negative feedback loop (DNFL) model. This model, which is similar to the clock model used by Goldbeter [33] and which was developed by Monk and Heron [34], [35], generates synthetic mRNA and protein data. By varying the parameters of the model it is possible to produce a range of periodic but non-sinusoidal waveforms including asymmetric cycles similar to those found in biological systems. Here we use parameter sets identical to those used in [14] to produce two sets of time series. The first comprised time series with a moderate level of asymmetry and the second comprised time series with a moderate shoulder. Examples of both the mathematical test signals and the DFNL time series are shown in Figures 1A, 1B and full details of the parameters can be found in [14].

Once the basic time series had been generated, noise was added. The noise was additive and was either uniform noise or walking noise. Uniform noise is drawn from a uniform distribution and the amplitude of the noise is defined as a percentage of the amplitude of the original time-series. This would be characteristic of the noise in a measurement system. Walking noise is additive and uniform, but this time the distribution of the noise is restricted so that the current data point lies within a limited range of the previous data point (Figures 1C, 1D). This would be more representative of noisy signals in nature, where noise affects an underlying biological system with a characteristic timescale greater than the sampling interval. The level of noise added is defined as the percentage of the amplitude of the original test signal (typically 30%, 80% or 160%), and is referred to in the text as, for example, 80% walking noise.

To assess applicability of the methods to the analysis of real biological systems, we also tested data sets obtained by *in vivo* imaging of transgenic *Arabidopsis thaliana* plants. Each transgenic line carried a luciferase reporter gene, in which a promoter of a gene of interest (termed the marker) is fused to the luciferase protein sequence. The expression of the luciferase protein was monitored by low-light imaging of seedlings, as described by Gould et al [36]. We analysed data acquired from transgenic plants having: CAB, CAT3, CCA1, CCR2 and TOC1 constructs [12]. During the whole measurement the plants were exposed to 24 h light/dark cycles, however some experienced 6 hours of light (short days: SD) while others received 18 hours (long days: LD). Plants in these conditions are expected to be stably entrained, with a period close to 24 h. Combining the 5 markers and 2 experimental conditions yielded 10 data sets with distinctive waveforms.

We report two metrics: the mean period and the mean absolute error, which is defined as absolute difference between the calculated value and the expected period (the means are calculated over the replicates in the tests data sets). The expected period value is 24.08 h for moderate asymmetry signal and 24.0 h in all the other cases. The mean period is abbreviated as MP in the tables and figures; absolute error (AE) refers to the mean absolute error. When discussed, statistical significance was determined by performing t-tests with alpha  = 0.05.

All the algorithms were implemented in Java using the Apache Math library for matrix operations, least-squares solving and FFT transform. The implementations are wrapped into web services using JAX-WS so they can be deployed on remote servers and linked with BioDare using the SOAP protocol. We introduced small modifications to the original algorithms, described below. All our implementations conduct detrending prior to analysis (cubic polynomial for SR, linear for the other methods).

Our implementation of EPR always uses spline interpolation to transform input data to time series with 0.1 hour data interval. This data step matches the 0.1 h period scanning step size. This approach has advantages over the original method when determining the spectrum power for a fractional period (for example 24.1), see SI (Doc S1) for more details.

For the MESA method, we initially followed Dowse [37] in using the Andersen algorithm [38] but it gave us poor period estimates probably due to its known numerical instability. The Barrodale implementation of MESA [39] addressed those issues, so this approach was selected, with a bi-directional prediction filter. However, that implementation underestimated the length of the internal prediction model. The minimal model lengths that gave good period estimates for our test data were therefore determined empirically and found to be specific for each sampling frequency.

The LSPR was implemented using the algorithm described by Glynn et al. [40].

We introduced two modifications to the SR algorithm. Firstly, when performing kernel smoothing only the close neighbourhood of the current point is taken into account instead of the whole time series. The size of the neighbourhood is determined by the bandwidth under consideration, and it is equal to the distance at which the ‘kernel’ value is smaller than 10^−14^. This reduces the asymptotic computational cost from O(N^2^) to O(N). The second modification concerns the selection of the optimal bandwidth, as we sample a smaller range of candidates than in the original paper. We compared the results obtained with the modified code against the estimates obtained using the original and found that neither modification influenced the period estimates, but both substantially reduced the computation time.

## Results

To evaluate the algorithms supported in BioDare, and to suggest guidelines for their use in circadian research, we compared the performance of Chi-square Periodogram (EPR), mFourfit (MFF), FFT-NLLS (NLLS), Maximum Entropy Spectral Analysis (MESA), Lomb-Scargle periodogram (LSPR) and Spectrum Resampling (SR).

### 1. Impact of increasing levels of noise

**Figure 2:**
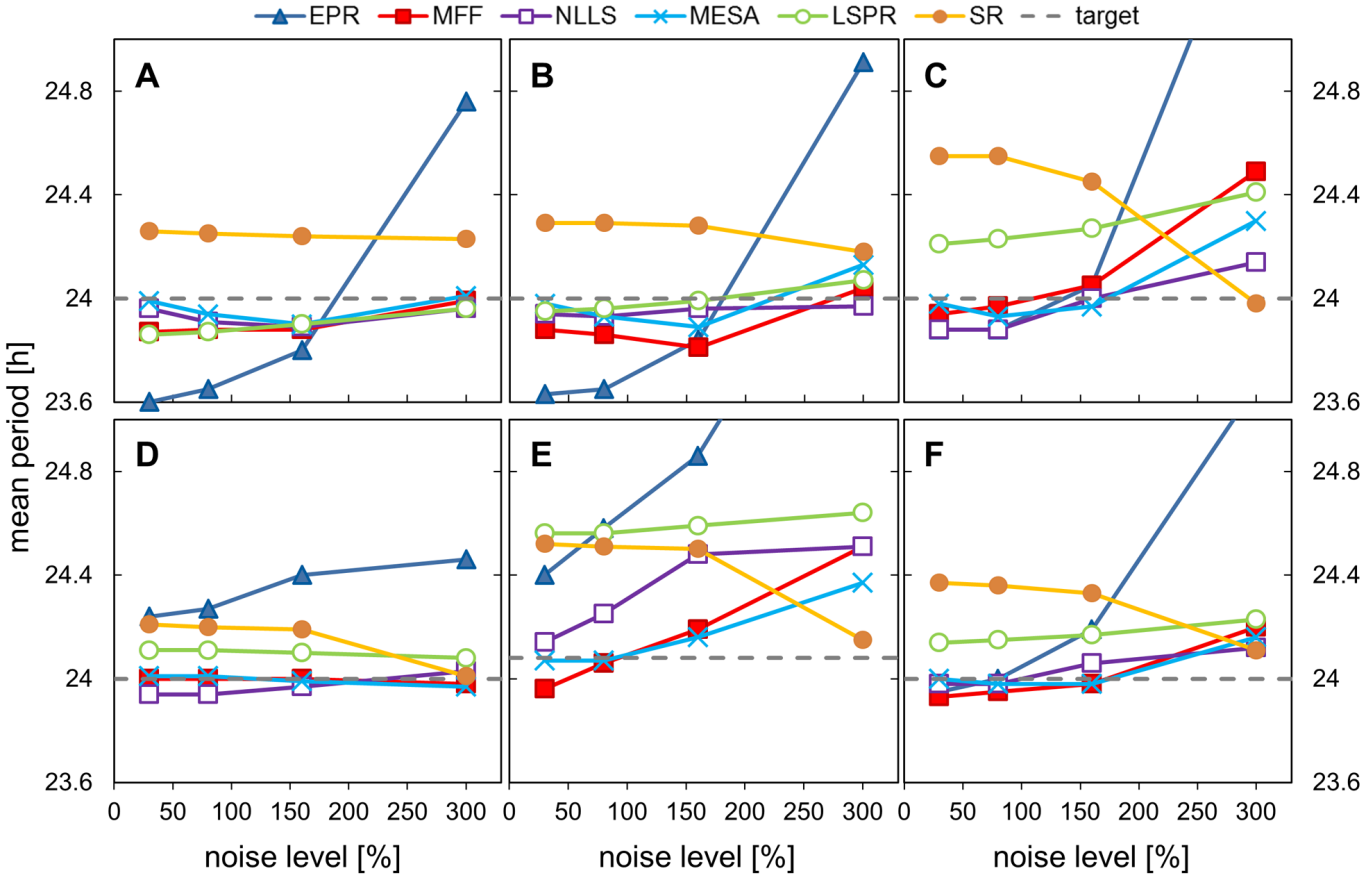
Impact of increasing levels of uniform noise on period estimation. Data sets with different noise levels (30%, 80%, 160%, 300%) were analysed using all the methods and the mean period was plotted. Data sets were created by adding noise at the level indicated to the hourly-sampled template of 3 days duration. The templates were: A) cosine data, B) pulse data, C) double pulse data, D) DNFL shoulder data, E) DNFL asymmetry data (expected period is 24.08 h), F) aggregated results from all the shapes.

**Figure 3:**
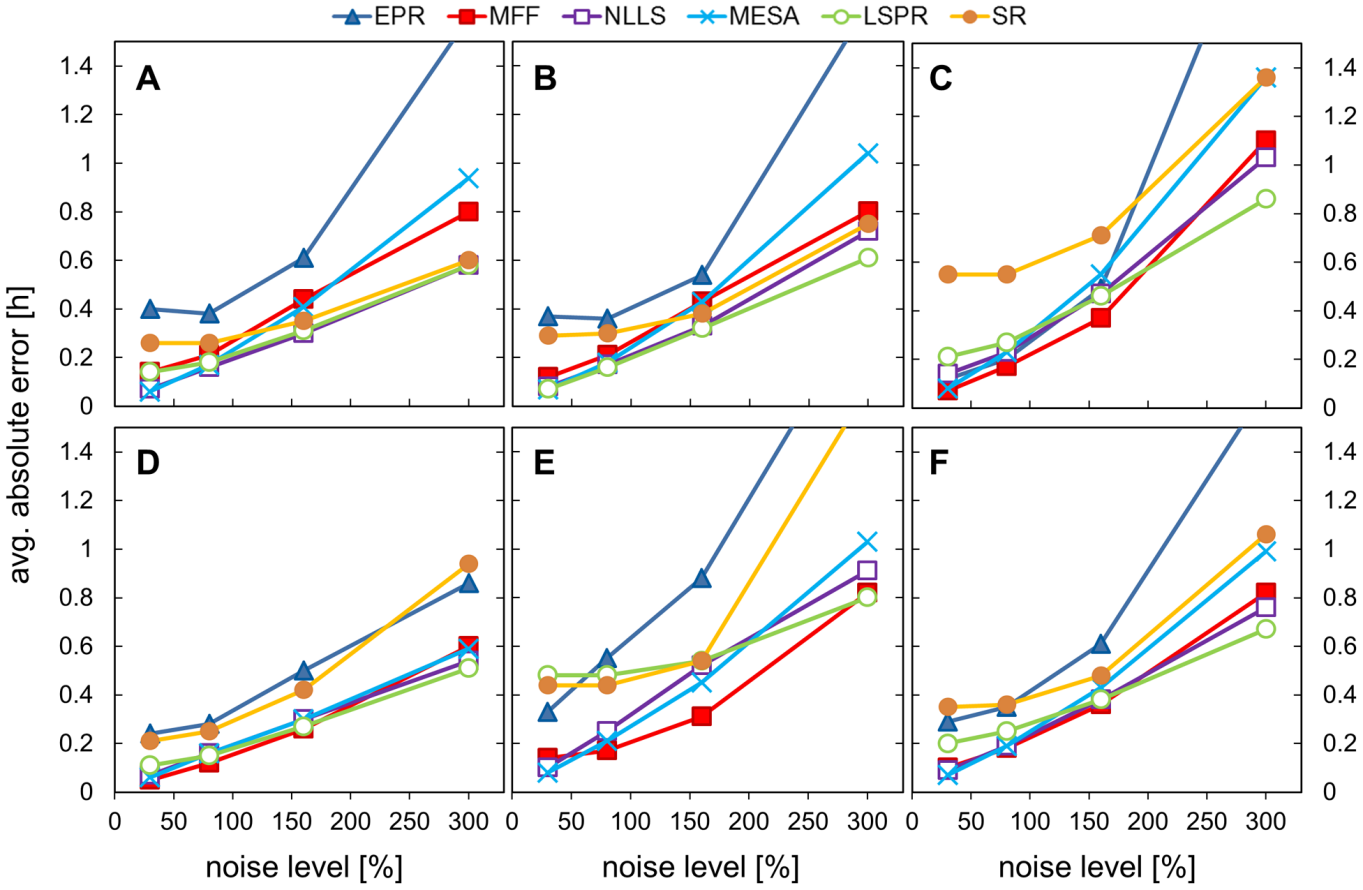
Impact of increasing levels of uniform noise on absolute error. Data sets with different noise levels (30%, 80%, 160%, 300%) were analysed using all the methods and the absolute error is plotted. The absolute error is defined as the absolute value of the difference between calculated period and the expected value (24.08 for asym. signal and 24 h for the others). Data sets were created by adding noise of specific level to the hourly-sampled template of 3 days duration. The templates were: A) cosine data, B) pulse data, C) double pulse data, D) DNFL shoulder data, E) DNFL asymmetry data, F) aggregated results from all the shapes.

We first examined the impact on rhythm analysis of a limited number of data points and increasing noise levels. Period estimation was carried out using only 3 days of data with sampling every hour, i.e. only 72 data points. The six algorithms were compared for different levels of noise (30%, 80%, 160% and 300%) and both uniform and walking noise were used. Figures 2,3 and Tables 1, 2 (full data are in SI Tables S1, S2) show the results for 5 different input signals. 3 mathematical test signals: cosine; pulse and double pulse: and two sets of artificial mRNA data, one with moderate asymmetry and one with a shoulder in the data, accompanied by the aggregated results from all the waveforms. 128 replicates with different noise samples were analysed by each algorithm. We report two metrics: the mean period MP (over the 128 replicates) and the mean absolute error AE, which is defined as absolute difference between the calculated value and the expected period (24.08 h for simulated mRNA data with moderate asymmetry and 24 h for the rest).

**Table 1:** Impact of uniform noise level on period estimates.

Shape^1^	Method	NL 30%^2^	NL 80%^2^	NL 160%^2^	NL 300%^2^
pul	EPR	23.63 (0.07)	23.65 (0.21)	23.84 (0.72)	24.91 (2.21)
pul	MFF	23.88 (0.07)	23.86 (0.22)	23.81 (0.46)	24.04 (1.11)+
pul	NLLS	23.94 (0.08)	23.93 (0.19)	23.96 (0.41)+	23.97 (1.37)+
pul	MESA	23.98 (0.08)	23.93 (0.22)	23.89 (0.55)	24.13 (1.52)+
pul	LSPR	23.95 (0.07)	23.96 (0.19)	23.99 (0.4)+	24.07 (0.81)+
pul	SR	24.29 (0.08)	24.29 (0.2)	24.28 (0.4)	24.18 (1.35)+
dblp	EPR	23.88 (0.07)	23.88 (0.22)	24.05 (0.69)+	25.63 (2.73)
dblp	MFF	23.94 (0.06)	23.97 (0.21)	24.05 (0.45)+	24.49 (1.78)
dblp	NLLS	23.88 (0.13)	23.88 (0.26)	24.0 (0.58)+	24.14 (1.9)+
dblp	MESA	23.98 (0.1)	23.93 (0.27)	23.97 (0.71)+	24.3 (1.95)
dblp	LSPR	24.21 (0.09)	24.23 (0.25)	24.27 (0.51)	24.41 (1.1)
dblp	SR	24.55 (0.1)	24.55 (0.25)	24.45 (1.2)	23.98 (2.73)+
all	EPR	23.95 (0.36)	24.0 (0.49)+	24.19 (0.82)	25.13 (2.16)
all	MFF	23.93 (0.08)	23.95 (0.22)	23.98 (0.45)+	24.2 (1.24)
all	NLLS	23.98 (0.13)	23.98 (0.26)	24.06 (0.5)	24.12 (1.33)
all	MESA	24.0 (0.1)+	23.98 (0.24)	23.98 (0.57)+	24.16 (1.42)
all	LSPR	24.14 (0.25)	24.15 (0.32)	24.17 (0.48)	24.23 (0.89)
all	SR	24.37 (0.17)	24.36 (0.27)	24.33 (0.69)	24.11 (2.39)+

Data sets with different noise level were analysed using all the methods. The mean period value is reported in the table (standard deviation is given in brackets). Data sets were created by adding noise of specific level to the hourly-sampled template of 3 days duration. 1) The base shape of the signal: cosine (cos), pulse (pul); double pulse (dpl); shoulder (shl) and moderate asymmetry (asym), (all) represents aggregated results from all the signals. 2) NL- noise level as the percentage of the original signal amplitude. +) Means which are accurate, not statistically different from the expected period value, are marked with +. The underlying period was 24.08 h for asym data and 24.00 h for the other signals. See SI Table S1 for the full results.

**Table 2:** Impact of uniform noise level on absolute error.

Shape^1^	Method	NL 30%^2^	NL 80%^2^	NL 160%^2^	NL 300%^2^
pul	EPR	0.37	0.36	0.54	1.65
pul	MFF	0.12	0.21	0.43	0.8
pul	NLLS	0.08	0.17	0.33	0.72
pul	MESA	0.07	0.18	0.43	1.04
pul	LSPR	0.07	0.16	0.32	0.61
pul	SR	0.29	0.3	0.38	0.75
dblp	EPR	0.12	0.2	0.49	2.16
dblp	MFF	0.07	0.17	0.37	1.1
dblp	NLLS	0.14	0.23	0.47	1.03
dblp	MESA	0.08	0.23	0.55	1.36
dblp	LSPR	0.21	0.27	0.46	0.86
dblp	SR	0.55	0.55	0.71	1.36
all	EPR	0.29	0.35	0.61	1.66
all	MFF	0.1	0.18	0.36	0.82
all	NLLS	0.09	0.19	0.38	0.76
all	MESA	0.07	0.19	0.43	0.99
all	LSPR	0.2	0.25	0.38	0.67
all	SR	0.35	0.36	0.48	1.06

Data sets with noise level were analysed using all the methods and the average absolute error is reported in the table. The absolute error is defined as the absolute value of the difference between calculated period and the expected value (24.08 for asym signal and 24 h for the others). Data sets were created by adding noise of given level to the hourly-sampled templates of 3 days duration. 1) The base shape of the signal: cosine (cos), pulse (pul); double pulse (dpl); shoulder (shl) and moderate asymmetry (asym), (all) represents aggregated results from all the sets2) NL- noise level as the percentage of the original signal amplitude. See SI Table S2 for the full table.

Period estimates depended upon the shape of the input signals and noise levels, with considerable differences among the algorithms. Analysis of method accuracy (how close the mean period was to 24 h) showed that MESA, MFF, NLLS gave the best period estimates for both low and high noise levels. EPR could either over- or under-estimate the mean period depending on the shape (Fig 2A,D), giving this method the largest estimation errors for high noise (Fig. 2C,E), whereas SR and LSPR (for double pulse, shoulder and moderate asymmetric shapes) tended to overestimate the period values (Fig. 2C,D,E). Double pulse and moderate asymmetry data were the most challenging, with the largest estimation errors (Fig. 2C,E). As expected, the average error increased with the level of noise, as individual input data traces were more severely distorted. However, the mean period reported by each of the methods was quite resilient to the amount of uniform noise added. There was little difference between the mean periods for 30% to 160% noise levels (relatively flat lines for the first 3 points in Fig. 2 with exception of 2E). EPR was the most sensitive to the amount of noise, and SR responded strongly to the highest 1.5 level. The MESA method gave the best results compared to the other algorithms for the highest noise level (MP in range 23.97-24.30), whereas EPR and SR performed poorly in this test (MP for EPR between 24.5 and 25.9, AE >1). Changes in period estimates due to the addition of the walking noise were more pronounced, but similar trends were observed (see SI, Figures S1, S2).

These results highlight the importance of measuring biological replicates when dealing with noisy data, as the mean period over the ‘population’ properly matched the underlying period even for substantially distorted signals. In general, MESA, MFF and NLLS offered comparable accuracy.

### 2. Impact of different signal durations

**Figure 4:**
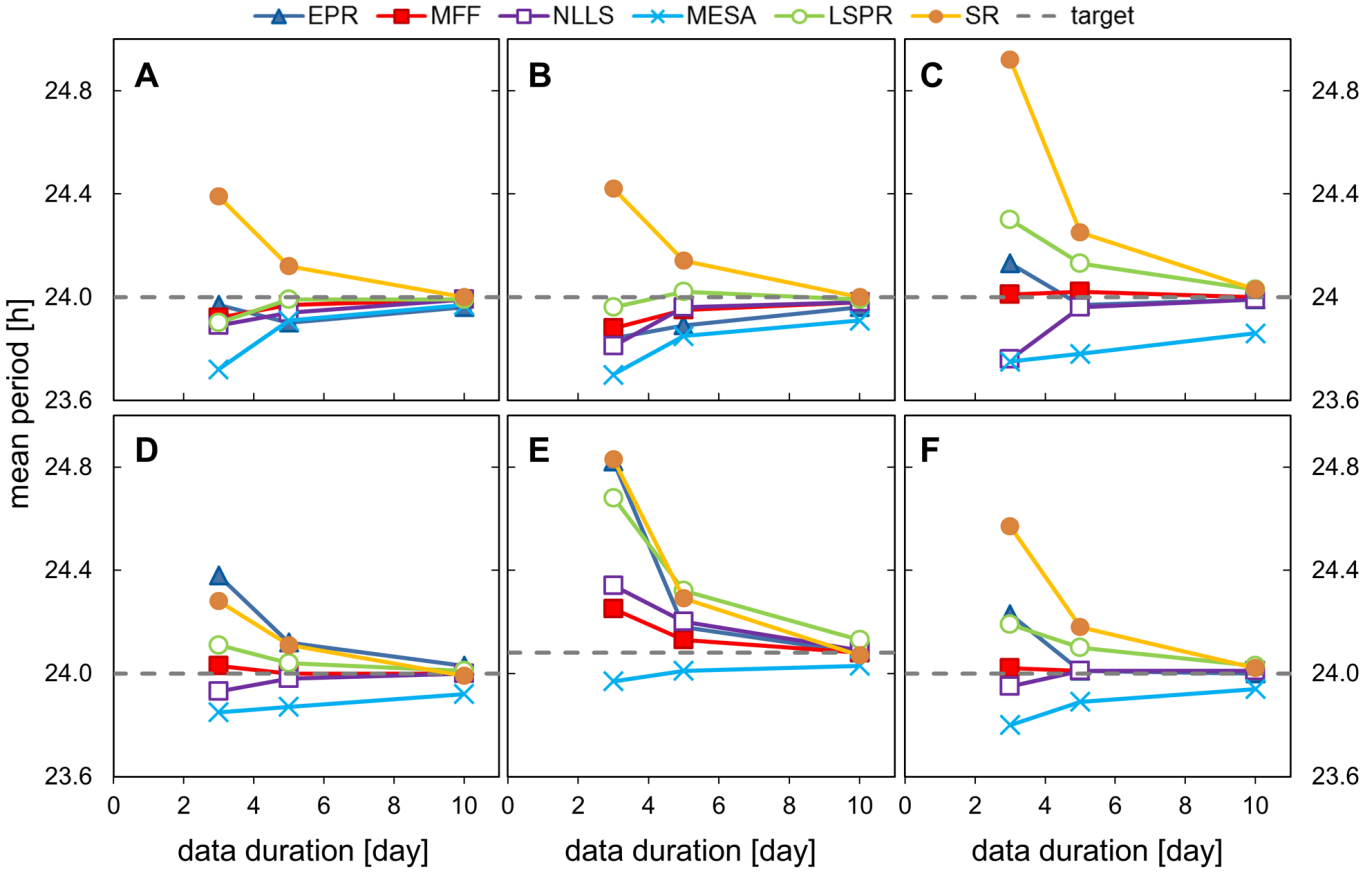
Impact of different signal durations on period estimation. Data sets with different signal duration were analysed using all the methods and the mean period value is presented for each test signal shape. Data sets were created by adding 160% walking noise to the hourly-sampled templates of different duration. A) cosine data, B) pulse data, C) double pulse data, D) DNFL shoulder data, E) DNFL asymmetry data (expected period is 24.08 h), F) aggregated results from all the shapes.

**Figure 5:**
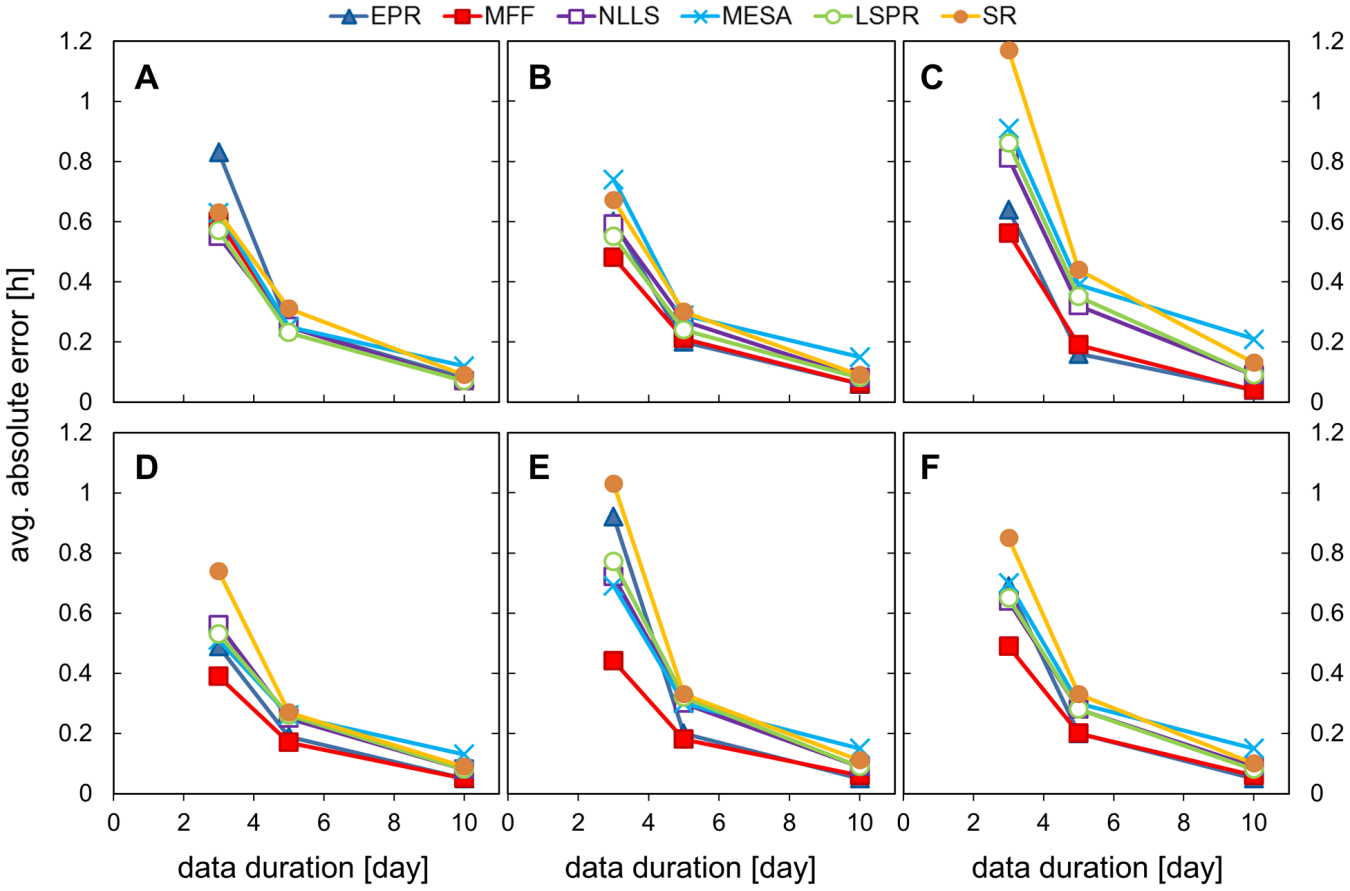
Impact of different signal durations on absolute error. Data sets with different signal durations were analysed using all the methods and the average absolute error is presented for each test signal shapes. Data sets were created by adding 160% walking noise to the hourly-sampled templates of different duration. A) cosine data, B) pulse data, C) double pulse data, D) DNFL shoulder data, E) DNFL asymmetry data, F) aggregated results from all the shapes.

The next analysis examined the effect of different signal durations on period estimation. Here the results of period estimation using 3, 5 and 10 days of data, all sampled every hour and with 160% walking noise added. The same input signal shapes were used, each with 128 replicates with different noise samples. Figures 4, 5 and Tables 3, 4 show the results of the analysis (full results set in SI Tables S3, S4).

**Table 3:** Impact of data duration on period estimates (data sets with walking noise).

Shape^1^	Method	3 days	5 days	10 days
shl	EPR	24.38 (0.52)	24.12 (0.23)	24.03 (0.07)
shl	MFF	24.03 (0.51)+	24.0 (0.21)+	24.0 (0.07)+
shl	NLLS	23.93 (0.7)+	23.98 (0.33)+	24.0 (0.11)+
shl	MESA	23.85 (0.65)	23.87 (0.33)	23.92 (0.15)
shl	LSPR	24.11 (0.65)	24.04 (0.32)+	24.01 (0.1)+
shl	SR	24.28 (0.91)	24.11 (0.33)	23.99 (0.11)+
asym	EPR	24.82 (1.08)	24.18 (0.24)	24.08 (0.06)+
asym	MFF	24.25 (0.57)	24.13 (0.21)	24.08 (0.07)+
asym	NLLS	24.34 (0.85)	24.2 (0.37)	24.09 (0.11)+
asym	MESA	23.97 (0.86)+	24.01 (0.38)	24.03 (0.18)
asym	LSPR	24.68 (0.78)	24.32 (0.34)	24.13 (0.1)
asym	SR	24.83 (1.14)	24.29 (0.39)	24.07 (0.13)+
all	EPR	24.23 (1.08)	24.01 (0.27)+	24.0 (0.09)+
all	MFF	24.02 (0.74)+	24.01 (0.25)+	24.01 (0.08)
all	NLLS	23.95 (0.82)+	24.01 (0.36)+	24.01 (0.12)
all	MESA	23.8 (1.07)	23.89 (0.37)	23.94 (0.19)
all	LSPR	24.19 (0.88)	24.1 (0.35)	24.03 (0.11)
all	SR	24.57 (1.1)	24.18 (0.4)	24.02 (0.17)

Data sets with different signal duration were analysed using all the methods. The mean period value is reported in the table (standard deviation is given in brackets). Data sets were created by adding walking noise of 160% of the original signal amplitude to the hourly-sampled templates of different length. 1) The base shape of the signal: cosine (cos), pulse (pul); double pulse (dpl); shoulder (shl) and moderate asymmetry (asym), (all) represents aggregated results from all the signals. +) Means which are accurate, not statistically different from the expected period value, are marked with +. The underlying period was 24.08 h for asym data and 24.00 h for the other signals. See SI Table S3 for the full table.

**Table 4:** Impact of data duration on absolute error (data sets with walking noise).

Shape^1^	Method	3 days	5 days	10 days
shl	EPR	0.49	0.19	0.05
shl	MFF	0.39	0.17	0.05
shl	NLLS	0.56	0.25	0.08
shl	MESA	0.51	0.26	0.13
shl	LSPR	0.53	0.26	0.08
shl	SR	0.74	0.27	0.09
asym	EPR	0.92	0.2	0.05
asym	MFF	0.44	0.18	0.06
asym	NLLS	0.72	0.3	0.09
asym	MESA	0.69	0.3	0.15
asym	LSPR	0.77	0.32	0.09
asym	SR	1.03	0.33	0.11
all	EPR	0.69	0.2	0.05
all	MFF	0.49	0.2	0.06
all	NLLS	0.64	0.28	0.09
all	MESA	0.7	0.3	0.15
all	LSPR	0.65	0.28	0.08
all	SR	0.85	0.33	0.1

Data sets with different signal duration were analysed using all the methods and the average absolute error is reported in the table. The absolute error is defined as the absolute value of the difference between calculated period and the expected value (24.08 for asym signal and 24 h for the others). Data sets were created by adding walking noise of 160% of the original signal amplitude to the hourly-sampled templates of different duration. 1) The base shape of the signal: cosine (cos), pulse (pul); double pulse (dpl); shoulder (shl) and moderate asymmetry (asym), (all) represents aggregated results from all the sets. See SI Table S4 for the full table.

As expected, accuracy of the period estimate improved and the error decreased as the duration of the time series, and hence the number of samples and cycles, increased. All methods gave almost perfect period estimates for 10 days of data (discrepancies below 0.1 h are insignificant in circadian applications as the typical biological variation is larger than 6 minutes). MESA tended to underestimate periods values (Fig. 4C, F) and its results for long data were statistically different from the other methods for all signal shapes apart from the simple cosine. Detailed examination of 3 days data with walking noise added revealed that MFF was the most accurate method for short timeseries (Fig. 5B–F), followed by NLLS and MESA, and the difference between those methods often was not statistically significant. The SR and EPR were the least suitable for analysis of such short data series, their absolute error values were in the range (0.6–1.2 and 0.5–0.9 respectively). In general SR overestimated period value (MP in the range of 24.3–24.9 h).

The acquired data suggests 5 days as a reasonable duration for circadian experiments aiming to estimate period. Compared to 10 days of data, 5 days data length is more technically feasible and should limit the impact of physiological changes in the studied samples. At the same time, the calculated mean period values lay close enough to the expected 24 hours and individual errors were below 0.2 h, which should be sufficient for typical applications. For 5 days data duration, MFF was generally the most accurate method (MP 24.0±0.05 for all shapes apart from asym., AE≈0.2), followed by NLLS, LSPR and MESA.

### 3. Impact on period estimation of varying the sampling frequency

**Figure 6:**
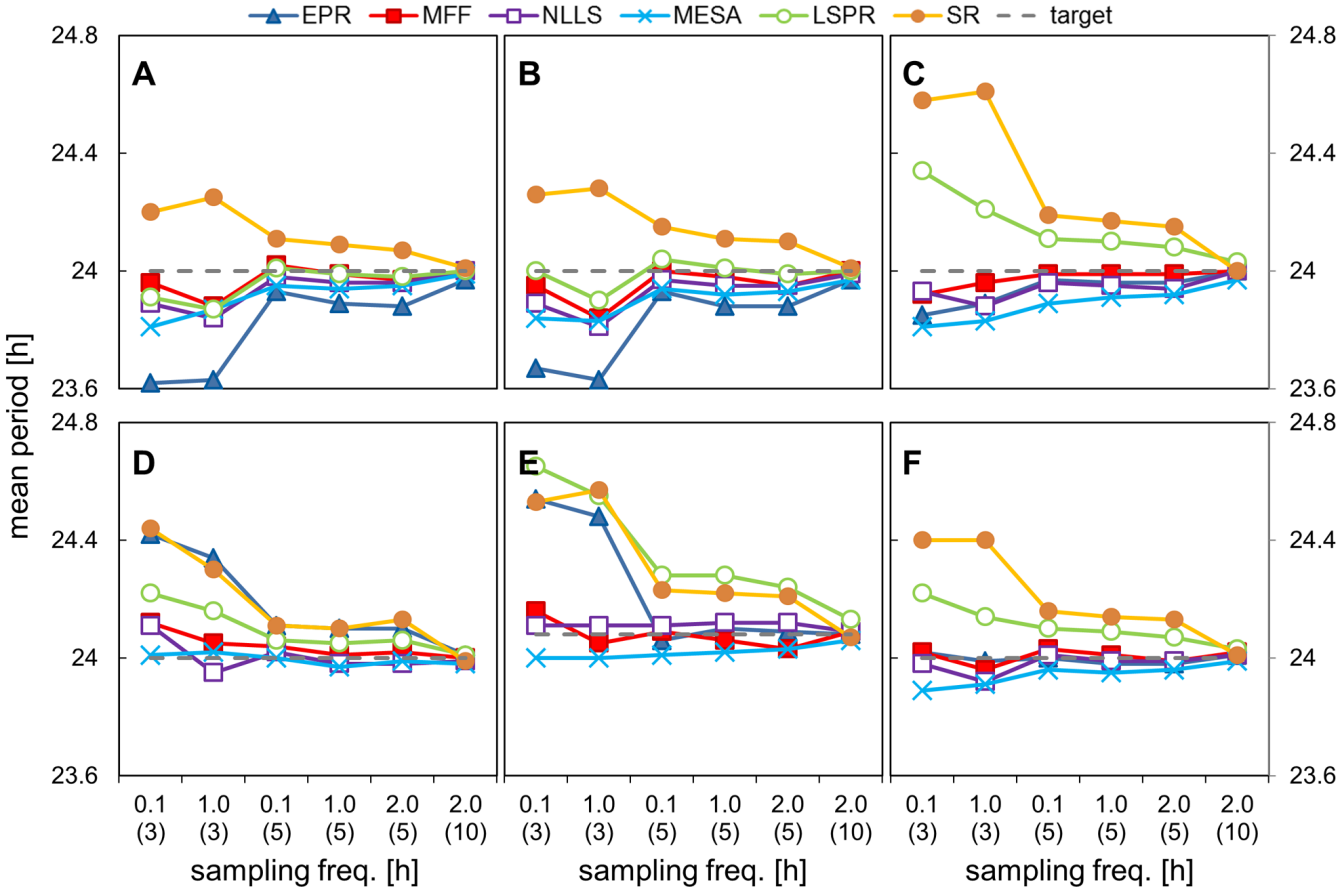
Impact of sampling frequency on period estimation. Data sets with different time intervals and selected durations were analysed using all the methods and the mean period value is plotted. Data sets were created by adding 80% walking noise to the templates of different duration and time interval between points. The X axis represents time intervals with data duration in brackets. The underlying period was 24.08 h for asym. data and 24.00 h for the other signals. A) cosine data, B) pulse data, C) double pulse data, D) DNFL shoulder data, E) DNFL asymmetry data, F) aggregated results from all the shapes.

**Figure 7:**
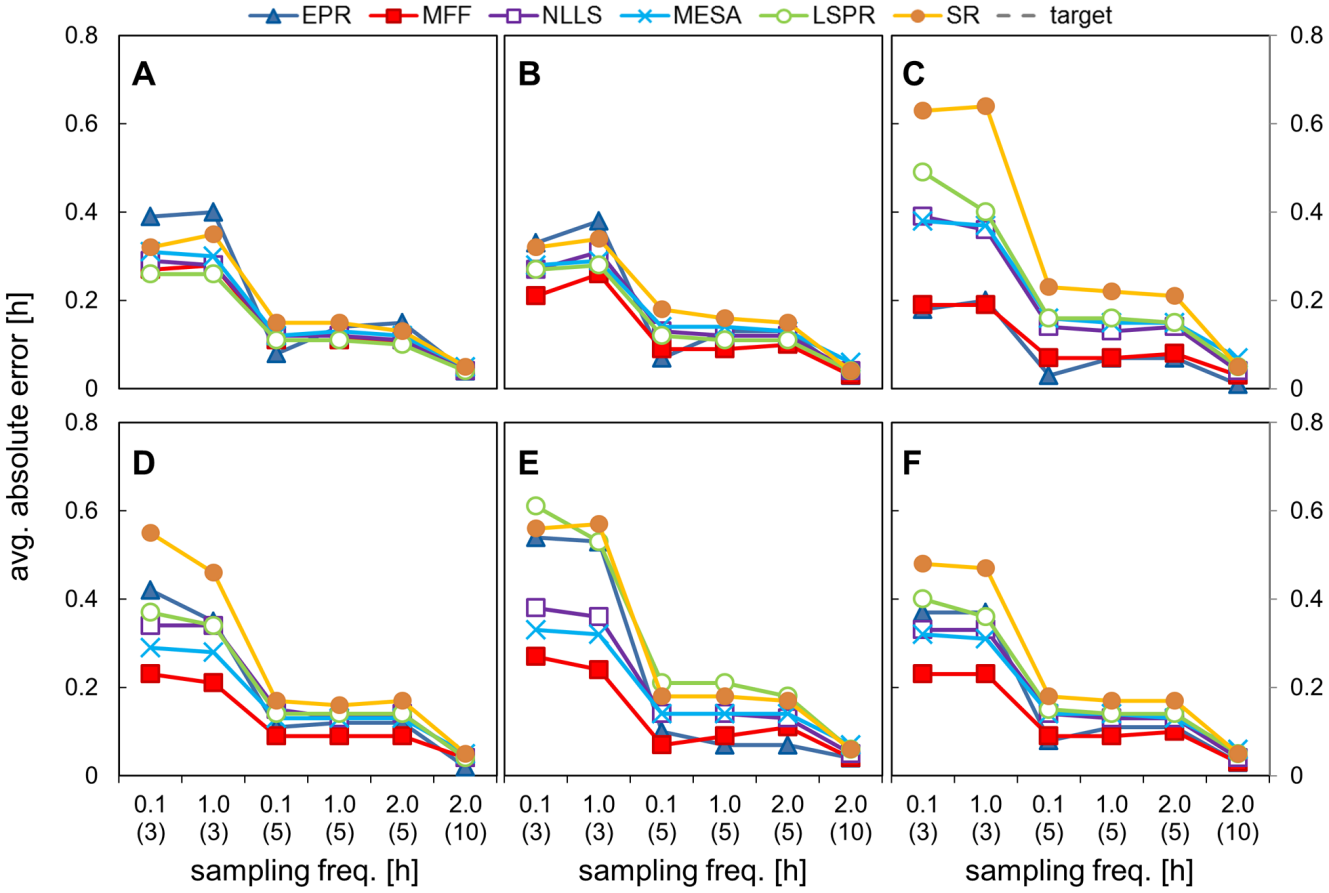
Impact of sampling frequency on absolute error (walking noise data set). Data sets with different time intervals and selected durations were analysed using all the methods and the mean absolute error value is plotted. Data sets were created by adding 80% walking noise to the templates of different duration and time interval between points. The X axis represents time intervals with data duration in brackets. The underlying period was 24.08 h for asym data and 24.00 h for the other signals. A) cosine data, B) pulse data, C) double pulse data, D) DNFL shoulder data, E) DNFL asymmetry data, F) aggregated results from all the shapes.

**Figure 8:**
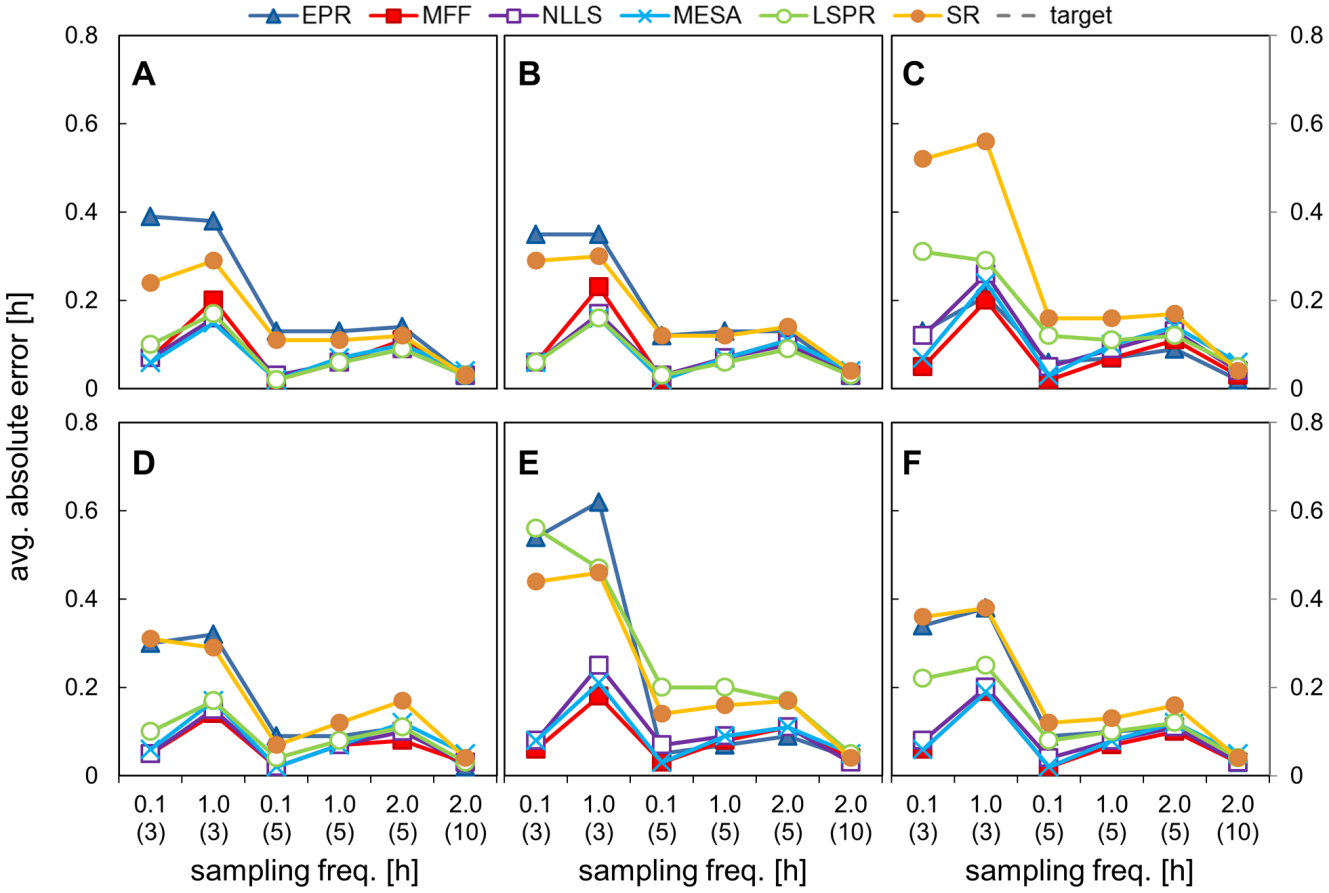
Impact of sampling frequency on absolute error (uniform noise data set). Data sets with different time intervals and selected durations were analysed using all the methods and the mean absolute error value is plotted. Data sets were created by adding 80% uniform noise to the templates of different duration and time interval between points. The X axis represents time intervals with data duration in brackets. The underlying period was 24.08 h for asym. data and 24.00 h for the other signals. A) cosine data, B) pulse data, C) double pulse data, D) DNFL shoulder data, E) DNFL asymmetry data, F) aggregated results from all the shapes.

In the third investigation we tested the influence of sampling frequency on the accuracy of period estimation. We used data with a noise level kept at 80% and we varied the sampling frequency between every 6 minutes, every hour and every 2 hours. The results are shown in Figures 6, 7, 8 and Tables 5, 6 (full results set in SI Tables S5, S6).

**Table 5:** Impact of sampling frequency on period estimates (data sets with walking noise).

Shape^1^	Method	0.1 (3.0)^2^	1.0 (3.0)^2^	0.1 (5.0)^2^	1.0 (5.0)^2^	2.0 (5.0)^2^	2.0 (10)^2^
cos	EPR	23.62	23.63	23.93	23.89	23.88	23.97
cos	MFF	23.96	23.88	24.02	23.99	23.97	24.00
cos	NLLS	23.89	23.84	23.98	23.96	23.96	24.00
cos	MESA	23.81	23.87	23.95	23.94	23.95	23.99
cos	LSPR	23.91	23.87	24.01	23.99	23.98	24.00
cos	SR	24.2	24.25	24.11	24.09	24.07	24.01
asym	EPR	24.54	24.48	24.06	24.1	24.09	24.08
asym	MFF	24.16	24.05	24.09	24.06	24.03	24.09
asym	NLLS	24.11	24.11	24.11	24.12	24.12	24.09
asym	MESA	24.00	24.00	24.01	24.02	24.03	24.06
asym	LSPR	24.65	24.55	24.28	24.28	24.24	24.13
asym	SR	24.53	24.57	24.23	24.22	24.21	24.07
all	EPR	24.02	23.99	24	23.98	23.98	24.01
all	MFF	24.02	23.96	24.03	24.01	23.99	24.02
all	NLLS	23.98	23.92	24.01	23.99	23.99	24.01
all	MESA	23.89	23.91	23.96	23.95	23.96	23.99
all	LSPR	24.22	24.14	24.1	24.09	24.07	24.03
all	SR	24.40	24.40	24.16	24.14	24.13	24.01

Data sets with different time intervals and selected durations were analysed using all the methods and the mean period value is reported in the table (standard deviations are omitted for clarity). Data sets were created by adding walking noise of 80% of the original signal amplitude to the templates of different duration and time interval between points. The underlying period was 24.08 h for asym data and 24.00 h for the other signals. 1) The base shape of the signal: cosine (cos), pulse (pul); double pulse (dpl); shoulder (shl) and moderate asymmetry (asym), (all) represents aggregated results from all the sets. 2) the time interval (sampling frequency) in the data set and in brackets the data duration. See SI Table S5 for the full table.

**Table 6:** Impact of sampling frequency on on absolute error (data sets with walking noise).

Shape^1^	Method	0.1 (3.0)^2^	1.0 (3.0)^2^	0.1 (5.0)^2^	1.0 (5.0)^2^	2.0 (5.0)^2^	2.0 (10)^2^
cos	EPR	0.39	0.4	0.08	0.14	0.15	0.04
cos	MFF	0.27	0.28	0.11	0.11	0.11	0.04
cos	NLLS	0.29	0.28	0.12	0.12	0.11	0.04
cos	MESA	0.31	0.3	0.12	0.13	0.12	0.05
cos	LSPR	0.26	0.26	0.11	0.11	0.1	0.04
cos	SR	0.32	0.35	0.15	0.15	0.13	0.05
asym	EPR	0.54	0.53	0.1	0.07	0.07	0.04
asym	MFF	0.27	0.24	0.07	0.09	0.11	0.04
asym	NLLS	0.38	0.36	0.14	0.14	0.13	0.05
asym	MESA	0.33	0.32	0.14	0.14	0.14	0.07
asym	LSPR	0.61	0.53	0.21	0.21	0.18	0.06
asym	SR	0.56	0.57	0.18	0.18	0.17	0.06
all	EPR	0.37	0.37	0.08	0.11	0.11	0.03
all	MFF	0.23	0.23	0.09	0.09	0.1	0.03
all	NLLS	0.33	0.33	0.14	0.13	0.13	0.04
all	MESA	0.32	0.31	0.14	0.14	0.13	0.06
all	LSPR	0.4	0.36	0.15	0.14	0.14	0.05
all	SR	0.48	0.47	0.18	0.17	0.17	0.05

Data sets with different time interval and selected durations were analysed using all the methods and the average absolute error is reported in the table, the absolute error is defined as the absolute value of the difference between calculated period and the expected value (24.08 for asym signal and 24 h for the others). Data sets were created by adding walking noise of 80% of the original signal amplitude to the templates of different duration and time interval between points. 1) The base shape of the signal: cosine (cos), pulse (pul); double pulse (dpl); shoulder (shl) and moderate asymmetry (asym), (all) represents aggregated results from all the sets. 2) the time interval (sampling frequency) in the data set and in brackets the data duration in days. See SI Table S6 for the full table.

The calculated mean periods and average errors were almost identical for all 3 sampling frequencies, in the case of 5 days of data with walking noise added (Fig. 6,7); the max difference between the MP for each frequency was 0.08 (SR analysis of shl. data). The sampling frequency had considerably less impact on period estimates than data duration or level of noise. The results for the data sampled 2-hourly over 10 days highlighted the importance of the data duration. Their average error was lower than for hourly or 6 minutes sampled 5 day data, although the former consisted of exactly the same number of data points and the latter had 10 times more (Fig. 7). In similar fashion, the estimates for 3-day data with 6 minutes time interval (720 timepoints) were less precise than hourly-sampled, 5-day data with only 120 measurements (Fig 6A–C).

Different conclusions could be drawn from the results obtained for the data with uniform noise. In this case, frequent sampling reduced the value of average error (Fig. 8, first two points in each panel). This is the consequence of different impact of both forms of noise on the waveform shape (see SI, Figure S3). Uniform noise added to densely-sampled data preserves the underlying shape, as there is high probability that the changes to the each of 10 points per hour would cancel each other. In contrast, walking noise ‘propagates’ between the points, and the final waveform for the 0.1 and 1-hourly data were both similarly distorted. Nevertheless, the results for 10 days data with 2 hour sampling interval were generally equal or more accurate than for shorter timeseries sampled every 6 minutes, despite having an order of magnitude fewer data points.

MFF was the best method for the analysis of the most ‘sparse’ data (5 days, 2-hourly samples) with, followed by NLLS and MESA, all 3 having MP in the range of 24.00±0.05 h; the difference between those methods was statistically significant only for dbl. pulse and asymmetric data.

### 4. Impact of non-sinusoidal data

One of the reported advantages of techniques which do not try to fit the data as a series of sines and/or cosines is that such techniques are able to perform better when analysing non-sinusoidal data. Looking back at results presented above, we could not prove such claims. The more demanding time series were: the double pulse mathematical test signal; and both simulated DNFL RNA time series (asymmetric and shoulder data). Those signals were neither constructed using trigonometric functions nor were symmetrical. Nevertheless both MFF and NLLS typically offered the best accuracy, which could be matched only by MESA from the non-sinusoidal methods.

### 5. Impact of non-evenly sampled data

In a typical circadian experiment data are collected at constant time intervals, however, due to technical problems, occasionally some of the data points have to be omitted and the final time series are no longer evenly spaced (for example, measurements of luminescence often contain cosmic-ray-induced spikes which have to be removed prior to period analysis). EPR, MESA and our implementation of SR require evenly spaced input data. LSPR and MFF do not have such restrictions, while NLLS is intermediate, as its core cosine fitting is performed using arbitrary input data times, but the initial parameters are established under the assumption of a regular time interval.

In order to test influence of irregular time intervals on the methods performance, we took hourly-sampled, 5-day-long data sets and randomly removed 2, 12, 24 and 36 data points (1%, 10%, 20% and 30% of the original signals). We then analysed the resulting time series using all the methods; in the case of EPR, MESA and SR, a simple interpolation was performed to provide the algorithms with necessary data regularity.

Unexpectedly, even the removal of the 30% of the time points had no effect on either mean period or average error (Table 7), despite the fact that the altered timeseries typically contained 5-6-hour gaps in their data. In this scenario, simple interpolation was enough to rectify the limitations of EPR, MESA and SR methods, without influencing their period estimates.

**Table 7:** Impact of non-evenly sampled data on period estimates.

Shape^1^	del.^2)^	Metric^3^	EPR	MFF	NLLS	MESA	LSPR	SR
pul	0	MP	23.88	23.98	23.95	23.92	24.01	24.11
pul	30%	MP	23.88	23.96	23.97	23.91	23.99	24.11
all	0	MP	23.99	24.01	23.99	23.95	24.09	24.14
all	30%	MP	23.99	24.00	24.04	23.95	24.07	24.14
pul	0	AE	0.13	0.09	0.12	0.14	0.11	0.16
pul	30%	AE	0.13	0.1	0.13	0.14	0.12	0.16
all	0	AE	0.11	0.09	0.13	0.14	0.14	0.17
all	30%	AE	0.11	0.1	0.15	0.15	0.16	0.17

The reference data sets together with data sets from which 30% data points were randomly removed, were analysed with all the methods. The mean period and the absolute error are reported in the table. The reference sets consisted of 5 days of hourly-sampled data, to which walking noise was added at 80% level of the original amplitude. 1) The type of the base signal, (pul) pulse, (all) aggregated results from all the used shapes (cosine, pulse, double pulse, shoulder and moderate asymmetry). 2) Percentage of randomly removed points (time series with 30% points missing usually contained at least one 5-hour gap in the data). 3) Values of mean period (MP) and absolute error (AE)

### 6. Influence of baseline trends

Period analysis methods usually assume only small variations in signal level and amplitude. To meet this assumption, most implementations have some form of detrending built into the data pre-processing steps. However in our experience, biological data from imaging experiments typically have significant baseline trends as well as changes in amplitude (for example, dampening due to the individual cellular rhythms losing synchrony). Thus, the next investigation considered the influence of baseline and amplitude trends on period estimation.

To examine the influence of baseline trends, 5 envelope shapes were applied to the pulse waveform used in the previous analyses. The envelopes comprised: linear increase; exponential increase; inverse parabola; 2/3 inverse parabola; and 1/3 parabola, see supplementary materials Figure S4 for more details and example time series. The maximum amplitude of each of the envelopes was varied from 0 (no baseline trend) to 100. In all cases the true underlying period was 24 hours and the data were sampled every hour for 5 days. 128 replicates were different noise samples (80% walking noise) were used and the average period for the 128 replicates was calculated. The results are shown in Table 8 and SI Table S7. In each case, the period estimates were accurate for baseline trends of equal amplitude to the 24 h rhythm, but were wildly overestimated when the algorithm confounded the trend with the 24 h rhythmic signal.

**Table 8:** Impact of baseline trend on periods estimates.

Trend^1^	Method	Level 0^2^	Level 1^2^	Level 5^2^	Level 10^2^	Level 20^2^
exp	EPR	23.88 (0.11)	23.87 (0.11)	23.65 (0.26)	23.05 (1.16)	35.1 (0)
exp	MFF	23.98 (0.11)	23.94 (0.13)	23.38 (0.1)	22.95 (0.08)	31.87 (5.31)
exp	NLLS	23.95 (0.15)	23.92 (0.14)	23.66 (0.28)	612.09 (657)	705.89 (227.48)
exp	MESA	23.92 (0.15)	23.92 (0.15)	23.92 (0.15)	23.9 (0.16)	23.88 (0.17)
exp	LSPR	24.01 (0.13)	23.92 (0.13)	23.45 (0.14)	22.88 (0.09)	35.63 (1.66)
exp	SR	24.11 (0.15)	24.11 (0.15)	24.1 (0.15)	24.1 (0.15)	24.09 (0.15)
ipar	EPR	23.88 (0.11)	23.8 (0.17)	34.61 (0.73)	34.62 (0.09)	34.6 (0.05)
ipar	MFF	23.98 (0.11)	23.76 (0.04)	34.62 (1.65)	34.9 (0.03)	34.85 (0.02)
ipar	NLLS	23.95 (0.15)	24.15 (0.25)	269.56 (77.77)	280.77 (23.87)	173.73 (120.04)
ipar	MESA	23.92 (0.15)	23.98 (0.15)	23.98 (0.18)	24.19 (0.22)	24.69 (0.54)
ipar	LSPR	24.01 (0.13)	24.31 (0.2)	34.88 (1.77)	35.3 (0.04)	35.31 (0.02)
ipar	SR	24.11 (0.15)	24.1 (0.15)	24.07 (0.15)	29.57 (6.6)	46.88 (0.5)

Data sets with different levels of baseline trend and different trend forms were analysed using all the methods and the mean period value is reported in the table (standard deviation is given in brackets). Data sets were created by taking a standard pulse signal data set (5 days data, hourly sampled, 80% walking noise level, 24 h underlying period) and adding to it 5 different envelope shapes with increasing amplitude. 1) The trend/envelope shapes: linear increase (lin); exponential increase (exp); inverse parabola (ipar); 2/3 inverse parabola (2/3ipar) and 1/3 parabola (1/3par). 2) The baseline level is defined as ration between trend total amplitude and the original signal amplitude (0 no trend, 20 trend is 20 times higher than signal). See SI Table S7 for the full table.

The inverse parabola was the most challenging form of the baseline trend for all the methods, while linear trend was correctly removed by all the implementations, because they have a linear detrending step built into the data pre-processing. The MESA method was the most resilient to the presence of large-scale baseline trends, with amplitudes up to 100-fold greater than the 24 h rhythmic signal (MP was in the range 24.0±0.2 for all trends apart from parabola). The other spectral-based method, SR, coped well with trends up to 20 times larger than the signal amplitude. However, SR incorporates third-order polynomial detrending in its data pre-processing, which the other methods lack. The results suggested that the other algorithms attempted to fit/report a large period component which corresponded to the envelope of the baseline trend. The implementations of EPR, LSPR, MFF were restricted to search for periods of up to 35 hours; for the large-amplitude trends, this value was reported instead of the underlying 24 h oscillations. NLLS was the most susceptible to the presence of trends. NLLS usually stopped after fitting only one, long-period cosine component that represented the trend, as the contribution of extra components with low amplitude to the fit was rejected as insignificant.

In summary, it would appear that large-amplitude trends in the baseline are challenging to all algorithms. It is worth noting that such time series are representative of real biological data. As a rule of thumb, if the trend in the data obscures the presence of the oscillations to the human eye, it also distorts period estimates. Our recommendation, therefore, would be to examine the input time series and apply an appropriate baseline detrending algorithm before attempting to use any of the period estimation methods.

### 7. Influence of amplitude trends

**Figure 9:**
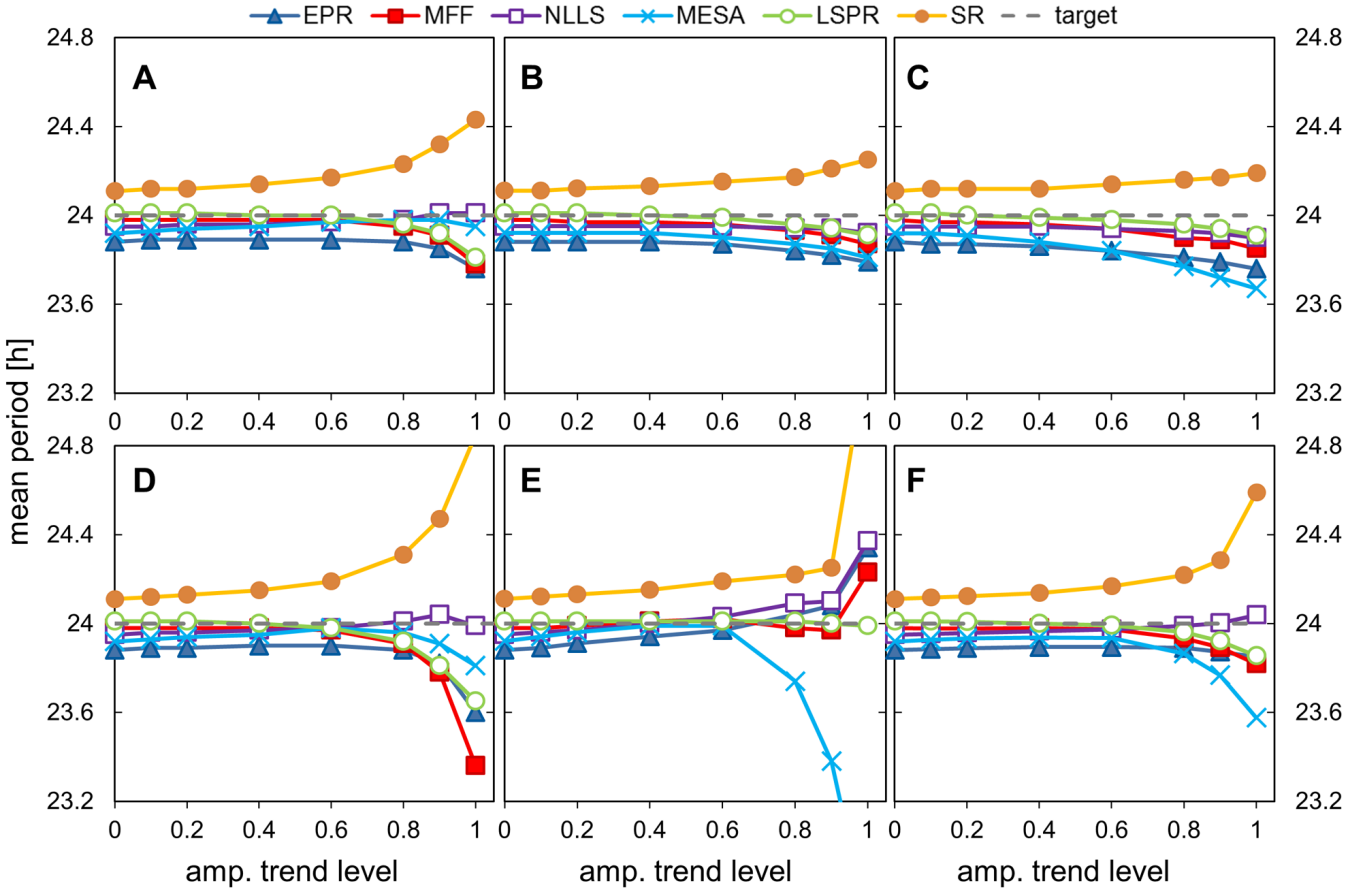
Impact of amplitude trends on period estimation. Data sets with different levels of amplitude trend and different trend forms were analysed using all the methods and the mean period value is plotted. The amplitude trends were obtained by dampening the test data sets to the stated level using different trend shapes/envelopes. Dampening was applied to a standard pulse data set (5 days data, hourly sampled, 80% walking noise level, 24 h underlying period), using 5 different envelope shapes with increasing amplitude. The level of amplitude trend, i.e. the maximal reduction of the original signal, is denoted as: 0, no dampening; and for example 0.6 for lin. trend means that at the end of 5^th^ day, the signal is reduced to 40% of its original value. The envelope shapes: A) exponential, B) linear, C) 1/3 parabola, D) 2/3 parabola, E) parabola, F) aggregated results from all the shapes.

Having examined the performance of the algorithms when subject to baseline trends, the influence of amplitude trends was then examined. Similar to the investigation into baseline trends, 5 envelope shapes were applied to the amplitude of the pulse waveform. The amplitude envelopes comprised: linear decrease; exponential decrease; parabola; 2/3 parabola; and 1/3 inverse parabola. The slope of each amplitude envelope was varied from 0 (no trend) to 1 (the amplitude has decayed to zero after 5 days; see supplementary materials Figure S5 for more details and example time series). In all cases the true underlying period was 24 hours and the data were sampled every hour for 5 days. 128 replicates with different noise samples (all walking noise, 80% amplitude) were used and the averaged period for the 128 replicates was calculated. The results are shown in Figure 9 and Table 9 (SI Table S8).

**Table 9:** Impact of amplitude trend on periods estimates.

Trend^1^	Method	0^2^	0.1^2^	0.2^2^	0.4^2^	0.6^2^	0.8^2^	0.9^2^	1^2^
exp	NLLS	23.95	23.95	23.96	23.96	23.97	23.98	24.01	24.01
exp	LSPR	24.01	24.01	24.01	24.00	24.00	23.96	23.92	23.81
exp	MESA	23.92	23.93	23.94	23.95	23.97	23.98	23.98	23.95
exp	MFF	23.98	23.98	23.98	23.98	23.98	23.95	23.91	23.78
exp	EPR	23.88	23.89	23.89	23.89	23.89	23.88	23.85	23.76
exp	SR	24.11	24.12	24.12	24.14	24.17	24.23	24.32	24.43
lin	NLLS	23.95	23.95	23.95	23.95	23.95	23.94	23.94	23.92
lin	LSPR	24.01	24.01	24.01	24.00	23.99	23.96	23.94	23.91
lin	MESA	23.92	23.92	23.92	23.92	23.9	23.87	23.85	23.81
lin	MFF	23.98	23.98	23.97	23.97	23.96	23.93	23.91	23.87
lin	EPR	23.88	23.88	23.88	23.88	23.87	23.84	23.82	23.79
lin	SR	24.11	24.11	24.12	24.13	24.15	24.17	24.21	24.25
2/3par	NLLS	23.95	23.96	23.96	23.97	23.98	24.01	24.04	23.99
2/3par	LSPR	24.01	24.01	24.01	24.00	23.98	23.92	23.81	23.65
2/3par	MESA	23.92	23.93	23.94	23.95	23.98	23.96	23.91	23.81
2/3par	MFF	23.98	23.98	23.98	23.98	23.97	23.91	23.78	23.36
2/3par	EPR	23.88	23.89	23.89	23.9	23.9	23.88	23.82	23.6
2/3par	SR	24.11	24.12	24.13	24.15	24.19	24.31	24.47	24.85

Data sets with different levels of amplitude trend and different trend forms were analysed using all the methods and the mean period value is reported in the table (standard deviations omitted for clarity). The amplitude trends were obtained by dampening the test data sets to the stated level at the last day using different trend shapes/envelopes. Dampening was applied to a standard pulse data set (5 days data, hourly sampled, 80% walking noise level, 24 h underlying period) and adding to it 5 different envelope shapes with increasing amplitude. 1) The trend/envelope shapes: linear decrease (lin); exponential decrease; parabola (par); 2/3 parabola (2/3par) and 1/parabola (1/3par) 2) The level of amplitude trend, ie, the maximal level of original signal deduction. 0 means no dampening, and for example 0.6 for lin. trend means that at the end of 5^th^ day, the signal is reduced to 40% of its original vale. See SI Table S8 for the full table.

Overall, the most striking result is that even substantial, non-monotonic amplitude trends do not pose a major challenge for these algorithms. Dampening of the signal by 40% of its initial strength (trend 0.4 in our notation) affected period estimates by any of the methods by <0.1 h, and <0.5 h for trend 0.8. Unlike in the case of baseline trends, SR and MESA had the largest sensitivity to the amplitude trends, while MFF and NLLS were usually the least sensitive.

These results suggest that no amplitude detrending is necessary to estimate period, even if the recorded signal loses half of its amplitude during the measurement interval. This is reassuring, because pre-processing for amplitude detrending typically distorts the shape of the data waveforms and is best avoided if possible.

### 8. Performance of the algorithms in classifying arrhythmic signals

Another aspect of period analysis is distinguishing between arrhythmic and rhythmic signals. However, the problem starts even with defining the arrhythmic signal. Even randomly created time series, can have some ‘structure’ due to its finite length, furthermore any noise will demonstrate itself as a high frequency component while a trend in the data will manifest itself in the low frequencies. For that reason, rather than expecting a positive identification of arrhythmic data, it is more reasonable to expect the absence of a period in the range of interest: we term this the weak arrhythmicity criterion. In this study, we define this range as 16–32 h; BioDare includes a similar, user-specified period selection range (18–30 h by default), and period values outside this range are ignored in summary statistics.

In the first experiment, we generated 100 time series with uniform noise and analysed them with the six methods (Table S9 in SI). NLLS reported 21 series as arrhythmic, LSPR dismissed 99 results for being below the significance threshold, while EPR dismissed only 6 of them (see SI). The reported period values for NLLS and SR were all (SR) or almost all (77/79, NLLS) outside the circadian range, so both methods passed our weak arrhythmicity criterion. EPR identified 32% circadian periods, failing to identify arrhythmia by the weak criterion. As MESA and MFF do not have any significance test, we used them over a wider period range (15–35 h), in case spurious periods were returned at one of the boundaries. However, most of the periods found by MESA (62%) and MFF (78%) were inside our range of interest (16–32 h), so those methods also returned false positives by our weak criterion.

There are two problems with this test. Firstly it is not realistic, as biologists would not usually analyse obviously arrhythmic data but signals with at least some oscillating pattern. Secondly, we cannot objectively specify how many of the data series should fail, as we cannot estimate their arrhythmicity independently: the reported period values might correctly match structure in the data that was introduced by the noise.

We addressed those problems by examining the performance of all algorithms in classifying a class of arrhythmic signals that is common in circadian experiments, where rhythmic amplitude collapses within the time series. Clock-regulated molecular rhythms often also respond to environmental light and/or temperature signals. These responses are usually retained even if the clock is otherwise disabled. Such molecular components exhibit driven rhythms in a rhythmic environment, but become arrhythmic in a constant environment. A transition from rhythmic to constant conditions is therefore a common part of circadian protocols, and a relevant case for analysis.

**Figure 10:**
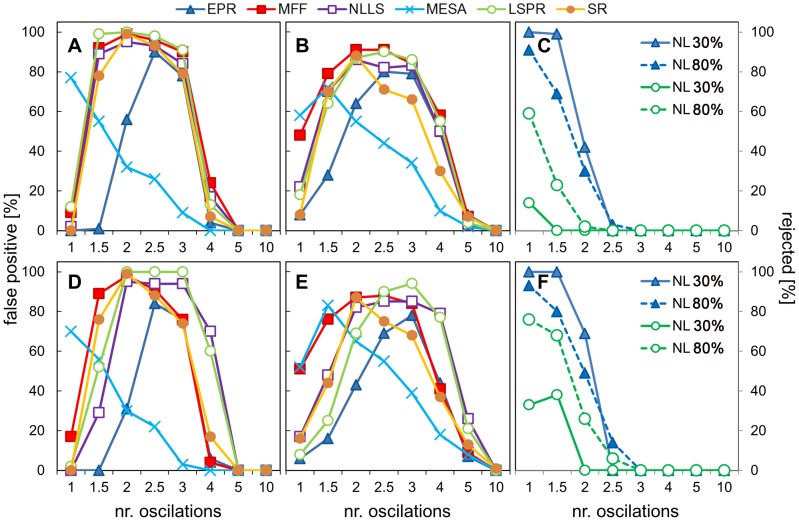
Performance of the algorithms in classifying arrhythmic signals. Data sets in which the rhythmic signal was reduced to 0 after a given number of cycles were analysed using all the methods and the percentage of false positives is plotted. A false positive was defined as a period value in the circadian range of interest (16–32 h) but not in the range of the true period (24.0±0.5 h). The test data were constructed by applying linear dampening to standard pulse or double pulse signals (5 days data, hourly sampled) in such a way that the signal amplitude was reduced to zero at 1, 1.5, 2, 2.5, 3, 4 or 5 days, thus preserving only the given number of original oscillations. 30% or 80% walking noise was then added. A), B) pulse signal with 30% and 80% walking noise respectively, D), E) double pulse signal with 30% and 80% respectively, C), F) percentage of results rejected by EPR and LSPR as being not significant for pulse and double pulse signal respectively.

To simulate this we took two mathematical test signals of known, 24 hour period, and we applied a linear dampening filter to them, such that the amplitude of the signal was reduced to zero after 1 day, 1.5 days, 2 days, 2.5 days, 3, 4, 5, or 10 days. Noise was then added at 30% or 80% amplitude of the original time series (see supplementary material Figure S6 for example time series). The rationale for this was that there need to be at least 2 cycles (so in this case 2 days) of data for the data to be identifiably periodic. Hence those time series which are reduced to zero after 1 day or 1.5 days should be classified as arrhythmic. For the data with slower dampening, if a period is identified then this period should be close to the known 24 hour period of the underlying signal within the time series as it should dominate over the noise factor. We define such a period as being “accurate” (defined as ±0.5 hours from the expected 24 hour period). Any period values which are in the circadian range but not ‘accurate’ can be treated as false positives, because unlike in the previous experiment, we know the underlying ‘structure’ of our signal. In this test we assumed circadian range to be (18–30 h), while the methods were used over the wider range of (15–35), again the reason for that was the expectation of spurious periods returned at one of the boundaries. The results are shown in Figure 10. When discussing these results, we refer to data with the rhythmic signal reduced to 0 after the 2^nd^ day as ‘2 days of data’, for example, though the full time series spanned 5 days.

The EPR correctly rejected as insignificant data with only 1 or 1.5 cycles of oscillation and the lower 30% noise level (Figure 10C,F). The rejection percentage was lower for the higher noise and only 70% of periods for 1.5 days of pulse data were marked as not significant (see SI Table S10). On the contrary, LSPR dismissed more results when higher noise was applied, reaching 70% for the double pulse, but only 60% for the single pulse signal (Figure 10C,F). NLLS could not identify any of the signals as arrhythmic. Hence, the significance threshold of the EPR could act as a test of arrhythmicity.

Analysing period values and using our weak arrhythmicity criterion, only MFF and MESA could not identify 1-day data as arrhythmic (Figure 10B,E). However, for 1.5 days data all the methods except EPR reported a majority of false-positive rhythms. MESA correctly assigned period values even for the signal which disappeared completely after the3^rd^ day, with less than 10% of false positives for the lower noise case and 30% for the higher noise (Figure 10A,B). Results from the other methods showed a majority of false-positive rhythms detected when data were dampened between 2 and 4 day.

The above results may seem to be in contradiction with the study of amplitude trends, during which MFF and NLLS performed better than MESA. However, in the former tests, the signal oscillates during the whole 5 days, while in the current test, the signal becomes a flat line after reaching the dampening threshold (once the noise is discarded). MFF and NLLS try to fit their model into this ‘flat’ section, which illustrates the main weakness of the curve fitting methods. Indeed, when we reanalysed 4-day data (80% noise) after truncating them to 72 hours, the rate of false positives for pulse and double pulse signals dropped to 13% and 35% for FFT, and to 30% and 12% for MFF. We also examined the estimation errors generated by the MFF, NLLS and SR algorithms in an attempt to find a threshold or metric which could be used to classify false positives but there was no pattern which could be used (see below).

Although it was not primary goal of these tests, their results revealed that MESA can give good periods estimates even for data with less than 3 full cycles of oscillation and is resilient to discontinuity of the signal. To some degree the EPR can act as an arrhythmicity test. We would therefore recommend discarding any signals that do not pass the EPR significance test.

### 9. Error measures

MFF, NLLS and SR provide various error measures in their output, which could be used in further reasoning about analysis results. MFF reports an Akaike Information Criterion value based on the goodness of fit to the data, modified by the number of cosine components used to model the data. We found it of limited use, as it is confounded by how sinusoidal the timeseries is, rather than giving a direct indication of how reliable the period estimate is. Both NLLS and SR calculate confidence intervals for their period estimates.

We investigated whether these error values could help to identify the false positives in the arrhythmicity tests described above. We calculated separate statistics on the error values reported for the false positive results and the accurate results (defined as being 24±0.5 h) Table 10. Unfortunately, no difference could be observed between those groups. For example SR reported about 30% false positives for pulse data dampened at the 4^th^ day. The average period confidence interval was about 1.3 for false positives and 1.2 for the accurate periods; similarly, the values for NLLS were 0.9 and 0.8 respectively.

**Table 10:** Period and confidence intervals for strongly dampened data.

Shape^1^	Method	Metric^2^	Dmp. 3^3^	Dmp. 4^3^	Dmp. 5^3^	Dmp. 10^3^
pul	NLLS	MP	24.71 (1.94)	24.02 (1.03)	23.89 (0.31)	23.96 (0.13)
pul	NLLS	CI (acc)	1.11 (0.27)	0.83 (0.14)	0.58 (0.08)	0.34 (0.04)
pul	NLLS	CI (false)	1.27 (0.5)	0.89 (0.17)	0.64 (0.18)	
dblp	NLLS	MP	25.04 (1.99)	24.48 (1.36)	23.87 (0.51)	23.95 (0.18)
dblp	NLLS	CI (acc)	1.49 (0.31)	1.16 (0.28)	0.79 (0.13)	0.46 (0.05)
dblp	NLLS	CI (false)	1.52 (0.47)	1.21 (0.28)	0.85 (0.15)	
pul	SR	MP	24.77 (0.74)	24.39 (0.4)	24.24 (0.26)	24.14 (0.14)
pul	SR	CI (acc)	1.67 (0.35)	1.2 (0.2)	0.95 (0.18)	0.78 (0.13)
pul	SR	CI (false)	1.74 (0.35)	1.3 (0.2)	1.03 (0.24)	
dblp	SR	MP	24.83 (1.01)	24.42 (0.51)	24.25 (0.34)	24.15 (0.19)
dblp	SR	CI (acc)	1.61 (0.49)	1.19 (0.21)	0.96 (0.17)	0.81 (0.11)
dblp	SR	CI (false)	1.75 (0.49)	1.2 (0.21)	0.97 (0.18)	

Data sets of strongly dampened data were analysed using SR and NLLS; the mean period and the mean confidence intervals are reported in the table (standard deviations are in brackets). The data sets were created by linear dampening the standard test data (5 days duration, hourly sampled, 24 h underlying period) in such a way that the signal reached 0 at the selected day, then uniform noise was added at 40% of the original amplitude. The results were classified to be accurate (acc) or false positives (false) depending on their period value: accurate periods were 24±0.5 h, while false positives were periods in the range (18–30 h) that were not accurate. 1) Shape of the base signal before dampening: pulse (pul) and double pulse (dblp). 2) The reported values are mean period (MP), and mean confidence intervals for accurate results (CI (acc)) and false positives (CI (false)). 3) Dampening level, represented as the day at which the initial rhythmic signal was reduced to 0, for example Dmp. 4 means that at the end of the 4^th^ day the signal was 0 and followed by a flat line (before adding the noise).

However, the values of confidence intervals can be used to reject individual results above some threshold, though the appropriate threshold is likely to vary among data sets. The mean periods obtained for the data dampened at 5th, 4th and 3rd day, could be classified as accurate, boundary and not-accurate. Hence, we chose the average confidence interval for the 4-day data as the guideline for the rejection threshold. We then reanalysed the results, rejecting periods with confidence level above 1.2. As can be seen in the Table 11 this procedure improved the accuracy of the mean period. For example, applying this classifier to analysis of 3-day data for the double pulse signal gave a mean period of 24 h from NLLS analysis instead of 25 h. The mean period from SR analysis was reduced to 24.42 from 24.83.

**Table 11:** Re-analysed results from the Table 10, using confidence interval threshold.

Shape^1^	Method	Metric^2^	Dmp. 3^3^	Dmp. 4^3^	Dmp. 5^3^	Dmp. 10^3^
pul	NLLS	MP	24.22 (1.75)	24.01 (1.03)	23.89 (0.31)	23.96 (0.13)
dblp	NLLS	MP	23.96 (2)	24.46 (1.3)	23.85 (0.49)	23.95 (0.18)
pul	SR	MP	24.03 (0.42)	24.3 (0.34)	24.23 (0.25)	24.14 (0.14)
dblp	SR	MP	24.42 (0.75)	24.27 (0.42)	24.23 (0.32)	24.15 (0.19)

The same data sets as in the Table 10 were analysed by SR and NLLS. Traces for which predicted confidence intervals were higher than 1.2 were rejected, before calculating the mean period of the remaining traces. 1), 2) and 3) as in the legend of Table 10.

A final application of the error measures is for visualising results. For example, NLLS calculates the relative amplitude error (amplitude error divided by the amplitude value), which increases from 0 to 1 as the amplitude nears statistical insignificance. BioDare users regularly utilize RAE scatter plots after NLLS analysis, which plot the RAE against the period value of individual traces (see SI Figure S7 for sample graphic). Such plots quickly highlight differences in the circadian system, for example between wild-type and mutant samples.

### 10. Analysis of biological data

**Figure 11:**
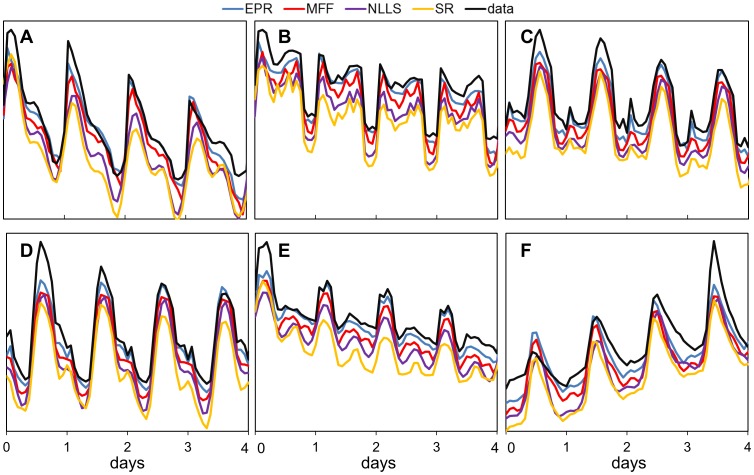
Examples of biological data. Selected traces of luciferase luminescence from transgenic Arabidopsis plants exposed to long day (LD) and short days (SD) light conditions. The original data are accompanied by the fits generated by the EPR, MFF, NLLS, and SR methods. For clarity, each time series was normalized to the maximum and then offset before plotting. The conditions and marker genes were: A) LD CAB, B) LD CAT3, C) LD CCR2, D) LD TOC1, E) SD CAT3, F) SD CCR2.

We analysed biological data obtained in luciferase imaging experiments, which is a common assay in the circadian field. The time series were obtained by measuring expression profiles of 5 different output genes in two light conditions (6 h light: 18 h dark cycles, SD; 18 h light: 6 h dark, LD), yielding 10 data sets, each with around 20 biological replicates. The selected data sets have three important features for this study: firstly each output gene has its own distinctive waveform (Figure 11) which is further altered by the light conditions (compare Figure 11B,C with 11E,F); secondly despite their different shapes, each timeseries is generated by the same underlying biological clock; and finally all the signals should have 24 h period as the system is being driven by the 24 h light:dark cycle (Note 2). Figure 11 presents examples of data traces for each output gene, together with waveforms fitted by four analysis method. Table 12 contains calculated period values, averaged over biological replicates.

**Table 12:** Analysis of biological data.

Data^1^	NLLS	LSPR	MESA	MFF	EPR	SR
All^2^	24.05 (0.9)+	24.27 (0.82)	24.06 (0.32)	24.15 (0.24)	24.11 (0.23)	23.13 (3.39)
LD CAB	24.37 (0.24)	24.63 (0.29)	24.35 (0.27)	24.36 (0.25)	24.35 (0.24)	24.51 (0.18)
LD CAT3	24.2 (0.27)	24.61 (0.32)	23.94 (0.3)+	24.05 (0.04)	24.1 (0)	14.87 (4.56)
LD CCA1	24.08 (0.21)+	24.31 (0.15)	24.05 (0.19)+	24.1 (0.12)	24.09 (0.1)	24.26 (0.18)
LD CCR2	23.92 (0.34)+	23.78 (0.3)	23.72 (0.22)	23.94 (0.21)+	23.95 (0.2)+	23.86 (0.27)
LD TOC1	24.11 (0.15)	23.89 (0.14)	23.93 (0.2)+	24.13 (0.13)	24.06 (0.07)	24.15 (0.16)
SD CAB	24.05 (0.21)+	24.13 (0.21)	24.05 (0.21)+	24.07 (0.14)	24.11 (0.27)+	24.23 (0.19)
SD CAT3	23.23 (2.79)+	24.6 (2.48)+	24.09 (0.36)+	24.11 (0.07)	24.04 (0.06)	20.95 (5.3)
SD CCA1	24.11 (0.13)	24.23 (0.11)	24.23 (0.18)	24.2 (0.08)	24.08 (0.08)	24.39 (0.14)
SD CCR2	24.16 (0.44)+	24.23 (0.49)	24.08 (0.4)+	24.17 (0.44)+	24.15 (0.46)+	24.22 (0.43)
SD TOC1	24.23 (0.25)	24.37 (0.25)	24.12 (0.33)+	24.32 (0.2)	24.17 (0.07)	24.23 (0.29)
NoCAT3^3^	24.13 (0.29)	24.19 (0.37)	24.06 (0.31)	24.16 (0.26)	24.12 (0.25)	24.23 (0.3)

Biological data were analysed with all 6 methods, the mean period value is reported in the table (standard deviation in brackets). The expected period is 24 h as the clock is entrained by a 24 h light:dark cycle. 1) The data were collected in two different conditions: LD and SD, monitoring 5 output genes in each of them. 2) (All) represents aggregated results from all data sets. 3) NoCAT3 represents aggregated results from all data sets except the CAT3 marker. +) The cases for which mean period is not statistically different from the 24 h are marked with +.

The biological data exposed another weakness of SR method. In the case of both *CAT3* data sets, the average period estimated by SR was considerably lower than 24 h. Inspection of the individual results revealed that for many data traces SR reported periods in the range of 12 h, reflecting the double peaks in *CAT3* data (Figure 11B,E). The key advantage of SR, which lies in finding the main frequency component, can also be a weakness if this main component is not in the circadian range. In such situations, NLLS found more than one frequency component but gave priority to the circadian one, while MFF and EPR only scanned for periods in the user defined range (here 15–35 h). Interestingly, both MFF and EPR continued to correctly find 24 h periods for *CAT3* data even after changing the scanning range to 10–35 h, to allow 12 h periods to be returned.

Based on the average deviation from 24 h, EPR seemed to be the best method (avg. deviation for all sets 0.12) and SR the worst (1.43 for all sets, or 0.27 when CAT3 data were excluded). However, half of the results of NLLS and MESA are statistically indistinguishable from the 24 h mean, and the average deviation is about 0.16 h. Also the results from NLLS, MFF and MESA were generally not statistically different from each other, which means that variation introduced by the biological replicates was larger than differences between those methods. Similarly to the artificial data, SR generally produced results which were statistically different from the other methods.

### 11. Computational complexity

Although not as scientifically important as the accuracy, the computation time of each algorithm is also an important factor when comparing the period analysis methods. Large computation costs may limit the utility of a method for large data sets or in construction of processing workflows, such as data clustering.

An analysis of the computational complexity of the algorithms was carried out by determining the number of operations required for each algorithm. EPR, LSPR and MFF all have an asymptotic cost of O(m*N), where N is number of data points and m the number of period values tested (typically m is approximately 100 for EPR and 500 for LSPR and MFF). They differ though by their scaling constants: EPR should have the lowest and MFF the highest due to performing more costly matrix operations and trigonometric calculations. MESA asymptotic cost is O(M^3^*N), where M is the length of the prediction model (in our tests for hourly sampled data M was around 40). The published version of Spectrum Resampling algorithm has cost of O(N^2^) due to the costly kernel smoothing. However, our implementation approximates the smoothing by taking into account only a limited neighbourhood and this reduces the cost to O(N). As the result the cost of the present Spectrum Resampling implementation is O(NlogN) as for longer data it becomes dominated by the computation of the Fourier transform. In contrast to the other methods, the computation cost of NLLS depends not only on the length of data but also on the waveform (and hence the number of parameters to be estimated) as well as the number of iterations taken to achieve convergence of the parameters. Thus the basic algorithm is linear in N, but the scaling constant varies enormously across the range 17 up to 10^9^ in the, highly unlikely, case where 25 cosines (and hence 76 parameters) are fitted to the data and it takes 500 (the maximum allowable) iterations for the parameters to converge.

It should be noted that biological time series tend to have limited duration and sampling resolution (typically less than 10 days of data, sampled no more than every 5 minutes), hence consideration of the asymptotic behaviour of the algorithms may be misleading as the ‘initial’ calculation steps or scaling constant may dominate the computation time. Having all the methods implemented in Java with the same setup, we had the unique opportunity to compare directly the running times of the different algorithms.

**Figure 12:**
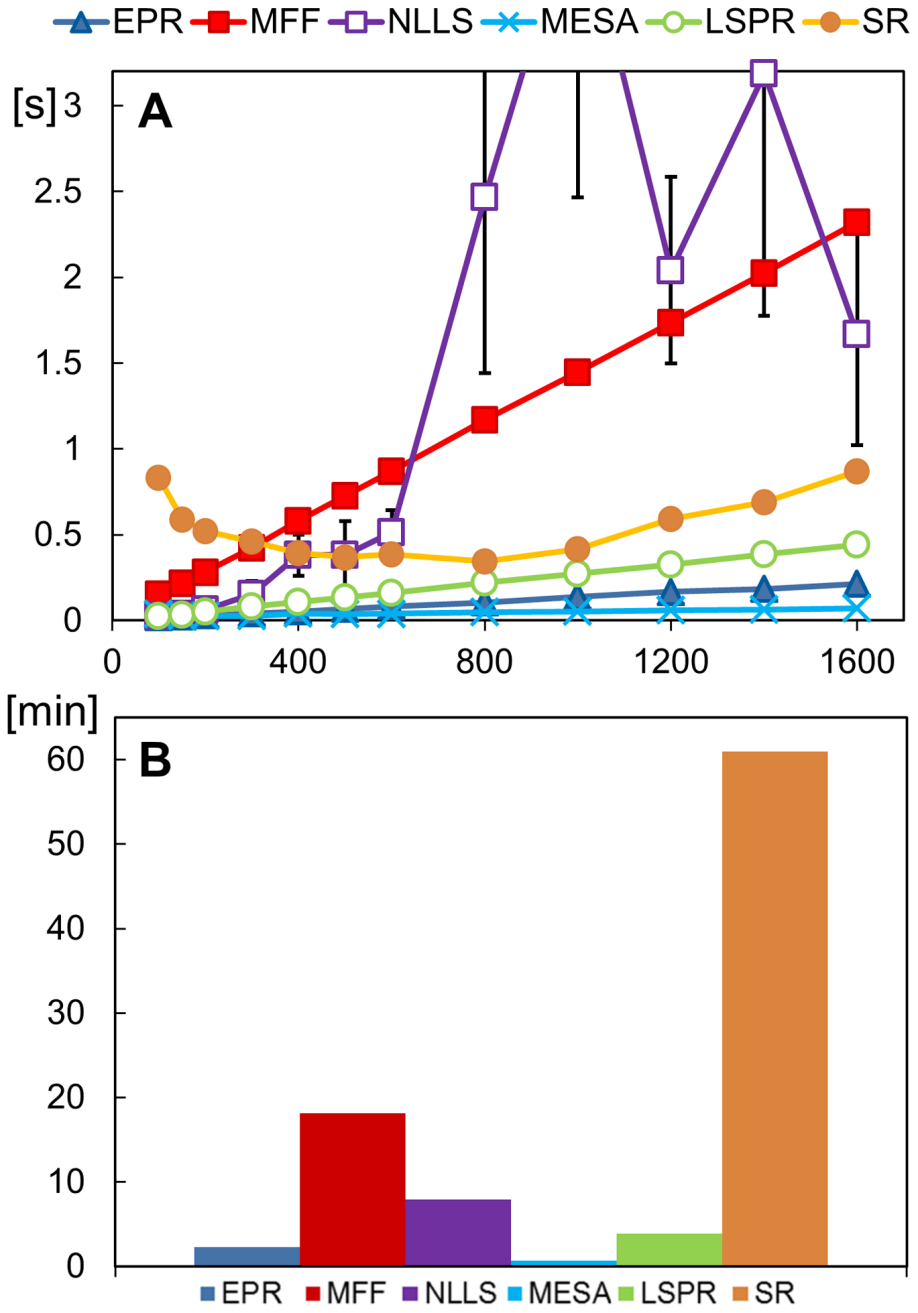
Methods computation time. A) Relationship between the number of data points and computational time for the different period estimation algorithms. The time series comprised artificial waveforms of different shapes including non-stationary periods or large noise levels were trimmed to the selected lengths and analysed with all the methods. Error bars for NLLS results show half of the standard deviation caused by analysis on different test data (there is no variance for other methods). B) Total computation time (minutes) for 2% of all data currently stored in the BioDare repository. Three samples of 2% of BioDare data (corresponding to 3000 time series, or 500,000 time points) were randomly selected and analysed using each method. The analysis time for each time series was recorded and these times summed to give total analysis time for that method; this was repeated 3 times for each data set. Averaged total time is presented, error bars are ignored as there was no significant difference between the test runs.

We first analysed synthetic data of different lengths to obtain the relationship between the number of data points and computation time. We generated a set of 7 time series each of 4000 hours and sampled every hour. The set contained artificial waveforms of different shapes including non-stationary period or large noise level (see supplementary materials for more details on the waveforms). The time series were trimmed to the selected lengths and analysed with all the period estimation algorithms. The processing time was recorded for the different lengths of time series and the results were averaged over 3 independent runs. The results are shown in Figure 12A.

All the methods with the exception of NLLS and SR show the predicted linear dependence on the number of data points. MESA is the quickest method, faster even than simple EPR most likely because our implementation of EPR operates on 10 times more data points and uses data structures instead of simple arrays as MESA. As predicted, MFF has the most steep profile from the methods of linear cost. As shown in Figure 12A, SR has a large overhead for short data. This is because it requires a minimum of 1000 data points for the analysis and the shorter data are always padded with zeroes up to this number. NLLS is fast for short data series but it shows high variability in computational time when longer data is used. For example, adding only one data point to a time series can change the computation time 10-fold (see SI Doc S2).

In order to test algorithm performance with typical biological data, we randomly selected 2% of time series (corresponding to about 3000 time series) stored in BioDare and analysed this subset using all the different algorithms. The analysis time for each time series was recorded and these times were summed to give total analysis time. This was repeated 3 times on different randomly selected data and the results are shown in Figure 12B. As expected, MESA processed all the data the quickest, the rest of the ranking was EPR, LSPR, NLLS, MFF and finally SR. SR was about 80 times slower than MESA. Even using the slowest algorithm, SR, the whole content of BioDare repository can be analysed in about 15 hours (in our current setup BioDare analyses 4 time series in parallel), which demonstrates that all the methods are suitable for real-world applications.

## Discussion

We have selected 6 methods which we believe represent popular approaches to period estimation for general time series data. We evaluated these algorithms under a range of conditions and for a wide variety of input signals of known period. Whilst some of the input signals were mathematical test signals, others were representative of real biological data.

Overall, it was found that MFF, NLLS and MESA gave the most accurate period estimates in almost all circumstances including so-called difficult scenarios, comprising short data sets and/or noisy data and/or low sampling rates, and non-sinusoidal signals. The difference between the accuracy of period estimates obtained from these three algorithms and the EPR, LSPR and SR is usually statistically significant in the less challenging conditions. It would also appear that SR gives the least accurate period estimate in the majority of cases.

In cases where there is a statistically significant difference in the accuracy of the period estimate obtained from the three best-performing algorithms, MFF tends to provide more accurate period estimates than NLLS. This is probably because FFT-NLLS algorithm attempts to “over-fit” by trying to fit a curve perfectly to the signal shape, including noise components that vary among cycles. MFF is constrained to fit a repeated pattern and so may be less influenced by local signal variations caused by noise. All of our test signals included a perfectly-repeating pattern of the type that MFF assumes, whereas this cannot be always guaranteed in biological data, for example if the physiological or developmental state of the sample changes within the experiment.

Previous work [14] has suggested that SR is more robust to non-sinusoidal patterns and observed noise than NLLS, which was also the main reason for which SR was incorporated into BioDare. However, we were unable to confirm such observations. After investigation we noticed, that there is a crucial difference between the way in which we and the other implementation handle the results of the NLLS method. The NLLS algorithm can identify multiple periodic components in the data, but in our approach it first reports the periods which lie within the user-defined range of interest (in the simulations reported here it was set to be between 15 and 35 hours). Costa et al. [14] did not apply such selection mechanism, but instead selected the period of the cosine component of the highest amplitude. We believe that it is usually reasonable to pre-select the period to lie within a certain range, in order to prevent the algorithm from selecting longer or shorter term trends which could mask the underlying circadian period. Typically the NLLS results contain a long period component that describes the trend in the data. The current users of NLLS are familiar this approach, as both BRASS and BioDare software automatically select the periods in a user-defined range.

We considered the impact of both amplitude and baseline trends on period estimation. Our results showed that even substantial amplitude trends did not pose a major challenge to the period estimation algorithm as long as about 3 full oscillations were present. In contrast, apart from MESA, none of the other algorithms was able to produce reliable period estimates in the presence of large-magnitude baseline trends. NLLS was especially susceptible to baseline trends as it tended to stop calculations after fitting to the data trend.

Finally, we have shown that the algorithms examined here are susceptible to false positives and will attempt to assign a period to a data series even when the underlying data are arrhythmic. Further, even examining the estimation errors associated with MFF, NLLS and SR algorithms, there does not seem to be a straightforward way of identifying these false positives. Only in limited circumstances EPR and LSPR rejected results for arrhythmic data based on the significance test.

Based on our results and analyses we would make the following recommendations:

If possible, data should span at least 5 cycles to obtain an accurate estimate of the period (where accurate is defined as ±0.5 hours for a 24 hour period) in all conditions tested here. If the objective is simply to classify the data as circadian or not, then 2 ½ cycles are sufficient. Accurate period estimates are possible from 3 cycles of data in favourable conditions.

Increasing the sampling rate, where experimental assays permit, does not offer substantial benefits in terms of improved period accuracy. For circadian data, sampling every hour gives accurate results. Analysis of transcriptome data suggested sampling every 2 h was sufficient in the different case of JTKcycle analysis [41].

It is important that baseline trends are removed prior to period estimation. We recommend the routine use of pre-processing to perform detrending and subsequent inspection of the resulting time series prior to period analysis. BioDare currently provides linear, cubic and local regression detrending.

Given the comparatively short processing time required for typical biological time series, we recommend using, as a minimum, both MFF and MESA to obtain a reliable estimate for the period. Both methods demonstrated good accuracy but they are based on completely different principles: MFF fits cosine-base curves, while MESA constructs a prediction model to perform spectral analysis. Consensus between those methods is a good indication of accurate period estimation. We favour NLLS over MFF, even though it gave slightly less precise results. NLLS provides error measures for the period, phase and amplitude. We routinely use those values to: a) weight the individual estimates when calculating population-wide summary statistics, b) reject individual results with high error levels, c) provide a second dimension when visualising analysis results (see SI). For more complete analysis, we recommend initial analysis using EPR, in order to decide which signals are arrhythmic and should be excluded from the processing by more accurate methods. Pre-selection of rhythmic traces is already routine for some model systems, for example in rhythmic locomotion assays of adult *Drosophila melanogaster*. A repository such as BioDare is extremely helpful in coordinating the results of multiple analyses, and this benefit grows as the number of different analyses increases. Further analytical methods will doubtless be required in future. The flexible software architecture of BioDare is designed to integrate further analytical methods, for example methods hosted as web services by the international chronobiology community and their collaborators.

## Supporting Information

Figure S1 **Impact of walking noise on mean period.** Data sets with different noise levels (30%, 80%, 160%, 300%) were analysed using all the methods and the mean period was plotted. Data sets were created by adding noise at the level indicated to the hourly-sampled template of 3 days duration. The templates were: A) cosine data, B) pulse data, C) double pulse data, D) DNFL shoulder data, E) DNFL asymmetry data (expected period is 24.08 h), F) aggregated results from all the shapes.
(TIF)

Figure S2 **Impact of walking noise on absolute error.** Data sets with different noise levels (30%, 80%, 160%, 300%) were analysed using all the methods and the absolute error is plotted. The absolute error is defined as the absolute value of the difference between calculated period and the expected value (24.08 for asym. signal and 24 h for the others). Data sets were created by adding noise of specific level to the hourly-sampled template of 3 days duration. The templates were: A) cosine data, B) pulse data, C) double pulse data, D) DNFL shoulder data, E) DNFL asymmetry data, F) aggregated results from all the shapes.
(TIF)

Figure S3 **Difference between frequently sampled data with uniform and walking noise added.**
(TIF)

Figure S4 **Shapes of baseline trends and examples of data with baseline trends applied.** A) Shapes of trend envelopes, B - C) data with trends applied. The trend shapes: exp: exponential; linear; inv. par: inverse parabola; 2/3 inv. par: 2/3 inverse parabola; and 1/3 par: 1/3 parabola.
(TIF)

Figure S5 **Shapes of amplitude trends envelopes and examples of data modified by them.** A-E) Data with trends applied, the trend shape and its levels are indicated on the graph. F) Shapes of trend envelopes.
(TIF)

Figure S6 **Data used for arhythmicity test.**
(TIF)

Figure S7 **Example of RAE plot for period analysis of WT and 3 mutants.**
(TIF)

Table S1 **Impact of noise level on mean period.**
(DOCX)

Table S2 **Impact of noise level on absolute error.**
(DOCX)

Table S3 **Impact of data duration on mean period.**
(DOCX)

Table S4 **Impact of data duration on absolute error.**
(DOCX)

Table S5 **Impact of sampling frequency on mean period.**
(DOCX)

Table S6 **Impact of sampling frequency on absolute error.**
(DOCX)

Table S7 **Impact of baseline trends on mean period.**
(DOCX)

Table S8 **Impact of amplitude trends on mean period.**
(DOCX)

Table S9 **Analysis of white noise signal.**
(DOCX)

Table S10 **Analysis of strongly dampened signals for arhythmicity test.**
(DOCX)

Doc S1 **Modification of EPR algorithm.**
(DOCX)

Doc S2 **NLLS computation time.**
(DOCX)

## References

[1] Mackey SR, Golden SS (2007) Winding up the cyanobacterial circadian clock. Trends in Microbiology 15 (9): 381–388. 17804240 10.1016/j.tim.2007.08.005

[2] Dong G, Golden SS (2008) How a cyanobacterium tells time. Current Opinion in Microbiology 11 (6): 541–546. 18983934 10.1016/j.mib.2008.10.003 PMC2692899

[3] Ukai H, Ueda HR (2010) Systems Biology of Mammalian Circadian Clocks. Annual Review of Physiology 72: 579–603. 10.1146/annurev-physiol-073109-130051 20148689

[4] Lowrey PL, Takahashi JS (2011) Genetics of Circadian Rhythms in Mammaliam Model Organisms. Advances in Genetics 74: 175–229. 21924978 10.1016/B978-0-12-387690-4.00006-4 PMC3709251

[5] Ishiura M, Kutsuna S, Aoki S, Iwasaki H, Andersson CR, et al (1998) Expression of a Gene Cluster kaiABC as a Circadian Feedback Process. Cyanobacteria Science 281: 1519–1523. 9727980 10.1126/science.281.5382.1519

[6] Millar AJ, Carre IA, Strayer CA, Chua N, Kay SA (1995) Circadian clock mutants in Arabidopsis identified by luciferase imaging. Science 267 (5201): 1161–1163. 7855595 10.1126/science.7855595

[7] Zhang EE, Liu AC, Hirota T, Miraglia LJ, Welch G, et al (2009) A genome-wide RNAi screen for modifiers of the circadian clock in human cells. Cell 139: 199–210. 19765810 10.1016/j.cell.2009.08.031 PMC2777987

[8] Maier B, Wendt S, Vanselow JT, Wallach T, Reischl S, et al (2009) A large-scale functional RNAi screen reveals a role for CK2 in the mammalian circadian clock. Genes Dev. 23: 708–718. 19299560 10.1101/gad.512209 PMC2661607

[9] Straume M, Frasier-Cadoret SG, Johnson ML (1991) Least Squares Analysis of Fluorescence Data. In: Lakowicz JR editor. Topics in Fluorescence Spectroscopy, Volume 2: Plenum. pp 117–240.

[10] Enright JT (1965) The search for rhythmicity in Biological Time-series. J. Theoret Biol. 8: 426–268. 5875312 10.1016/0022-5193(65)90021-4

[11] Lomb NR (1976) Least-squares frequency analysis of unequally spaced data. Astrophys. Space Sci. 39: 447–462.

[12] Edwards KD, Akman OE, Knox K, Lumsden PJ, Thomson AW, et al (2010) Quantitative analysis of regulatory flexibility under changing environmental conditions. Molecular Systems Biology 6: 424. 21045818 10.1038/msb.2010.81 PMC3010117

[13] Burg JP (1972) The relationship between maximum entropy spectra and maximum likelihood spectra. Geophysics 37: 375–376.

[14] Costa MJ, Finkenstädt B, Roche V, Lévi F, Gould PD, et al.. (2013) Inference on periodicity of circadian time series. Biostatistics: 1–15. 10.1093/biostatistics/kxt020 PMC3988453 23743206

[15] Moore A, Zielinski T, Millar AJ (2014) Online Period Estimation and Determination of Rhythmicity in Circadian Data Using the BioDare Data Infrastructure, In: Staiger D. Plant Circadian Networks: Methods and Protocols. Methods in Molecular Biology Series: Humana Press. in press. 10.1007/978-1-4939-0700-7_2 24792042

[16] Refinetti R (2004) Non-stationary time series and the robustness of circadian rhythms. Journal Theoretical Biology 227: 571–581. 10.1016/j.jtbi.2003.11.032 15038991

[17] Refinetti R (1992) Laboratory instrumentation and computing: comparison of six methods for the determination of the period of circadian rhythms. Physiology & Behaviour 54: 869–875. 10.1016/0031-9384(93)90294-p 8248375

[18] Lichtenberg U, Jensen LJ, Fausbøll A, Jensen TS, Bork P, et al (2005) Comparison of computational methods for the identification of cell cycle-regulated genes. Bioinformatics 21(7): 1191–1201. 10.1093/bioinformatics/bti093 15513999

[19] Sokolove PG, Bushell WN (1978) The Chi Square Periodogram: Its Utility for Analysis of Circadian Rhythms. J. Theor. Biol. 72: 131–160. 566361 10.1016/0022-5193(78)90022-x

[20] Johnson M (2010) Essential Numerical Computer Methods. Elsevier.

[21] Johnson ML, Frasier SG (1985) Nonlinear Least Squares Analysis. Methods Enzymol 117: 301–342.

[22] Bloomfield P (2000) Fourier Analysis of Time Series: An Introduction. Oxford: Wiley-Blackwell.

[23] Dowse HB, Ringo JM (1989) The Search for Hidden Periodicities in Biological Time Series Revisited. J. Theor. Biol. 139: 487–515.

[24] Trancart T, Lambert P, Rochard E, Daverat F, Coustillas J, et al (2012) Alternative flood tide transport tact in caradromous speciese: Anguilla anguilla, Liza ramada and Platichthys flesus. Estuarine Coastal and Shelf Science 99: 191–198.

[25] Darnell MZ, Rittschof D, Forward, Richard B (2010) Endogenous swimming rhythms underlying the spawning migration of the blue crab, Callinectes sapidus: ontogeny and variation with ambient tidal regime. Marine Biology 157(11): 2415–2425.

[26] Halberg F, Tong YL, Johnson EA (1967) Circadian system phase, an aspect of temporal morphology: procedures and illustrative examples. In: Mayersbach H, editor. The Cellular Aspects of Biorhythms. Berlin: Springer. pp 20–48.

[27] Leise TL, Harrington ME (2011) Wavelet-Based Time Series Analysis of Circadian Rhythms. J. Biol Rhythms 26: 454–463. 21921299 10.1177/0748730411416330

[28] Price TS, Baggs JE, Curtis AM, FitzGerald GA, Hogenesch JB (2008) WAVECLOCK: Wavelet analysis of circadian oscillation. Bioinformatics 24(23): 2794–2795. 18931366 10.1093/bioinformatics/btn521 PMC2639275

[29] Bretthorst GL (1988) Bayesian Spectrum Analysis and Parameter Estimation, Lecture Notes in Statistics, 48: Springer-Verlag.

[30] Cohen AL, Leise TL, Welsh DK (2012) Bayesian statistical analysis of circadian oscillations in fibroblasts. J. Theor. Biol. 314: 182–191. 22982138 10.1016/j.jtbi.2012.08.038 PMC3478438

[31] Locke JCW, Southern MM, Kozma-Bognar L, Hibberd V, Brown PE, et al (2005) Extension of a genetic network model by iterative experimentation and mathematical analysis. Molecular Systems Biology 1: 13. 10.1038/msb4100018 PMC1681447 16729048

[32] Southern MM, Millar AJ (2005) Circadian genetics in the model higher plant, Arabidopsis thaliana. Methods Enzymol 393: 23–35. 15817285 10.1016/S0076-6879(05)93002-4

[33] Goldbeter A (1991) A minimal cascade model for the mitotic oscillator involving cyclin and cdc2 kinase, PNAS. 88: 9107–9111. 10.1073/pnas.88.20.9107 PMC52661 1833774

[34] Monk NAM (2003) Oscillatory expression of Hes1, p53 and NF-κB driven by transcriptional time delays. Current Biology 13: 1409–1413. 12932324 10.1016/s0960-9822(03)00494-9

[35] Heron EA, Finkenstädt B, Rand DA (2007) Bayesian inference for dynamic transcriptional regulation: the hes1 system as a case study. Bioinformatics 23: 2596–2603. 17660527 10.1093/bioinformatics/btm367

[36] Gould PD, Locke JC, Larue C, Southern MM, Davis SJ, et al (2006) The molecular basis of temperature compensation in the Arabidopsis circadian clock. Plant Cell 18: 1177–1187. 16617099 10.1105/tpc.105.039990 PMC1456873

[37] Dowse HB (2013) Maximum entropy spectral analysis for circadian rhythms: theory, history and practice. J. Circ. Rhythms 11: 6. 10.1186/1740-3391-11-6 PMC3723481 23844660

[38] Andersen N (1974) On the calculation of filter coefficients for maximum entropy spectral analysis. Geophys 39: 69–72.

[39] Barrodale I, Erickson RE (1980) Algorithms for least-squares linear prediction and maximum entropy spectral analysis. Geophysics 45: 420–432.

[40] Glynn EF, Chen J, Mushegian AR (2006) Detecting periodic patterns in unevenly spaced gene expression time series using Lomb-Scargle periodogram. Bioinformatics 22: 310–316. 16303799 10.1093/bioinformatics/bti789

[41] Hughes ME, DiTacchio L, Hayes KR, Vollmers C, Pulivarthy S, et al (2009) Harmonics of Circadian Gene Transcription in Mammals. PLoS Genet 5(4): e1000442. 19343201 10.1371/journal.pgen.1000442 PMC2654964

