# Supplementary figures and images for: Strengths and Limitations of Period Estimation Methods for Circadian Data
Source: PLoS One. 2014 May 8;9(5):e96462. doi: 10.1371/journal.pone.0096462 (PMC4014635; doi:10.1371/journal.pone.0096462)

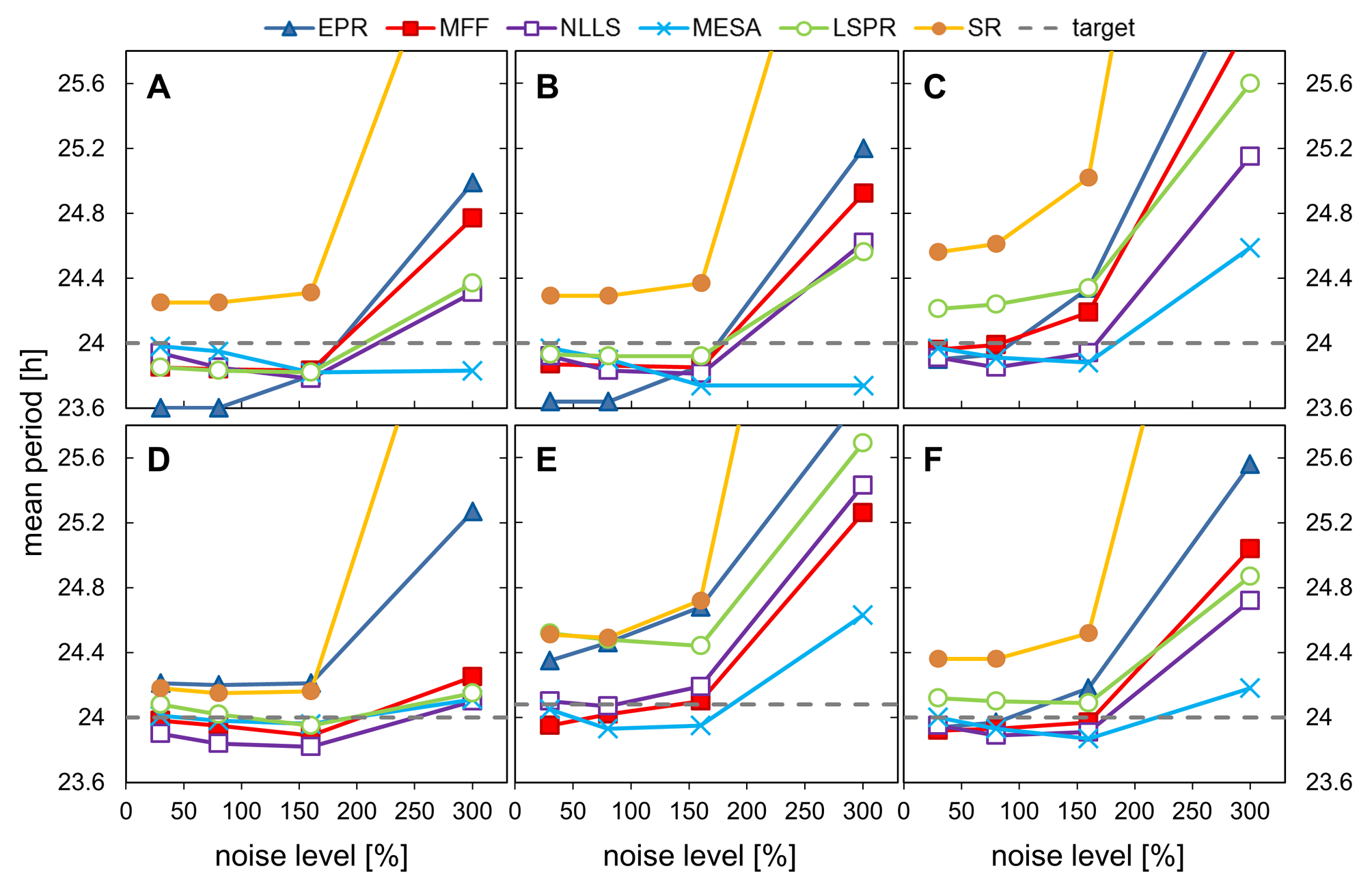

Supplement: Figure S1 — Impact of walking noise on mean period. Data sets with different noise levels (30%, 80%, 160%, 300%) were analysed using all the methods and the mean period was plotted. Data sets were created by adding noise at the level indicated to the hourly-sampled template of 3 days duration. The templates were: A) cosine data, B) pulse data, C) double pulse data, D) DNFL shoulder data, E) DNFL asymmetry data (expected period is 24.08 h), F) aggregated results from all the shapes. (TIF) [file pone.0096462.s001.tif]

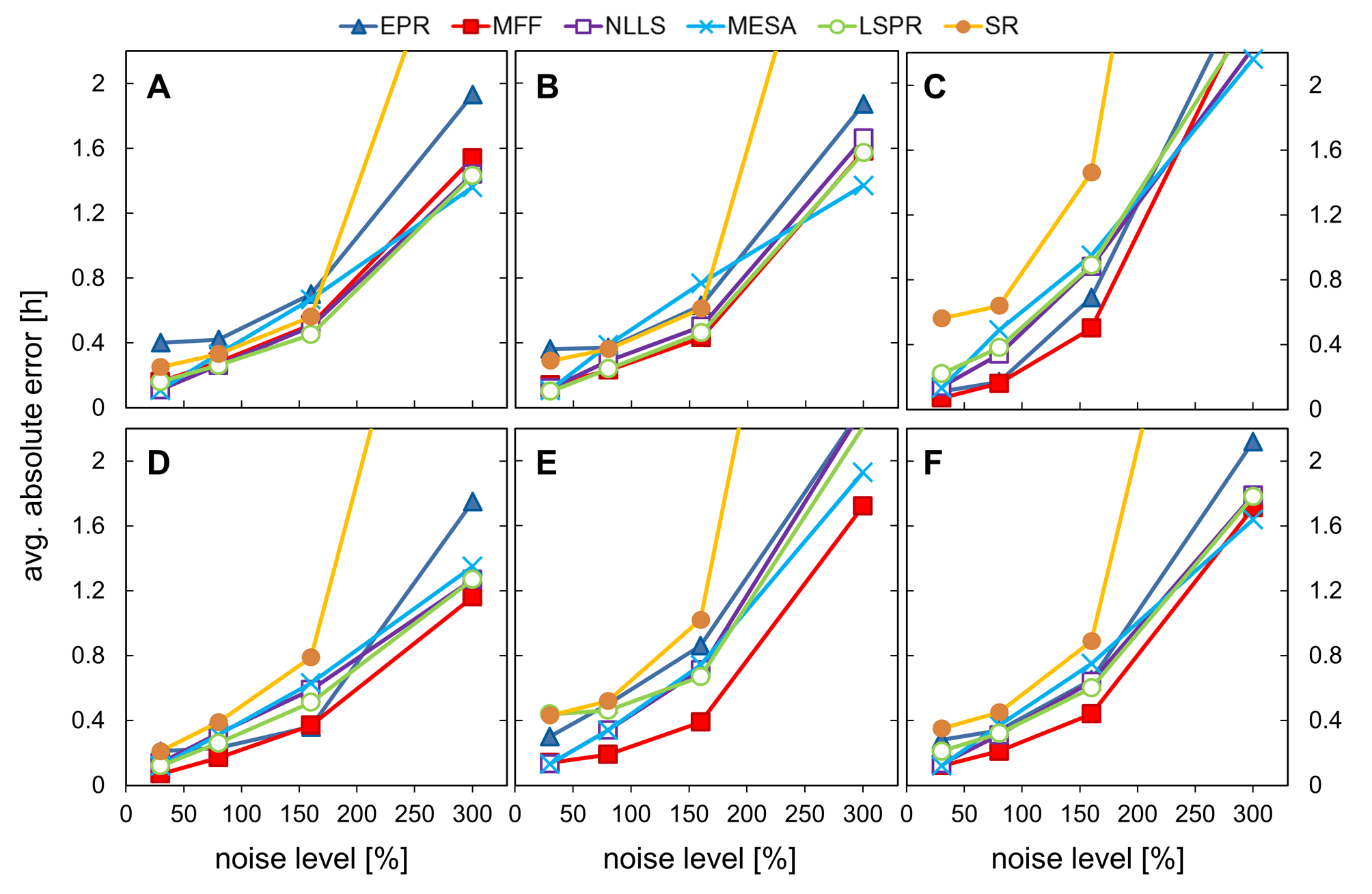

Supplement: Figure S2 — Impact of walking noise on absolute error. Data sets with different noise levels (30%, 80%, 160%, 300%) were analysed using all the methods and the absolute error is plotted. The absolute error is defined as the absolute value of the difference between calculated period and the expected value (24.08 for asym. signal and 24 h for the others). Data sets were created by adding noise of specific level to the hourly-sampled template of 3 days duration. The templates were: A) cosine data, B) pulse data, C) double pulse data, D) DNFL shoulder data, E) DNFL asymmetry data, F) aggregated results from all the shapes. (TIF) [file pone.0096462.s002.tif]

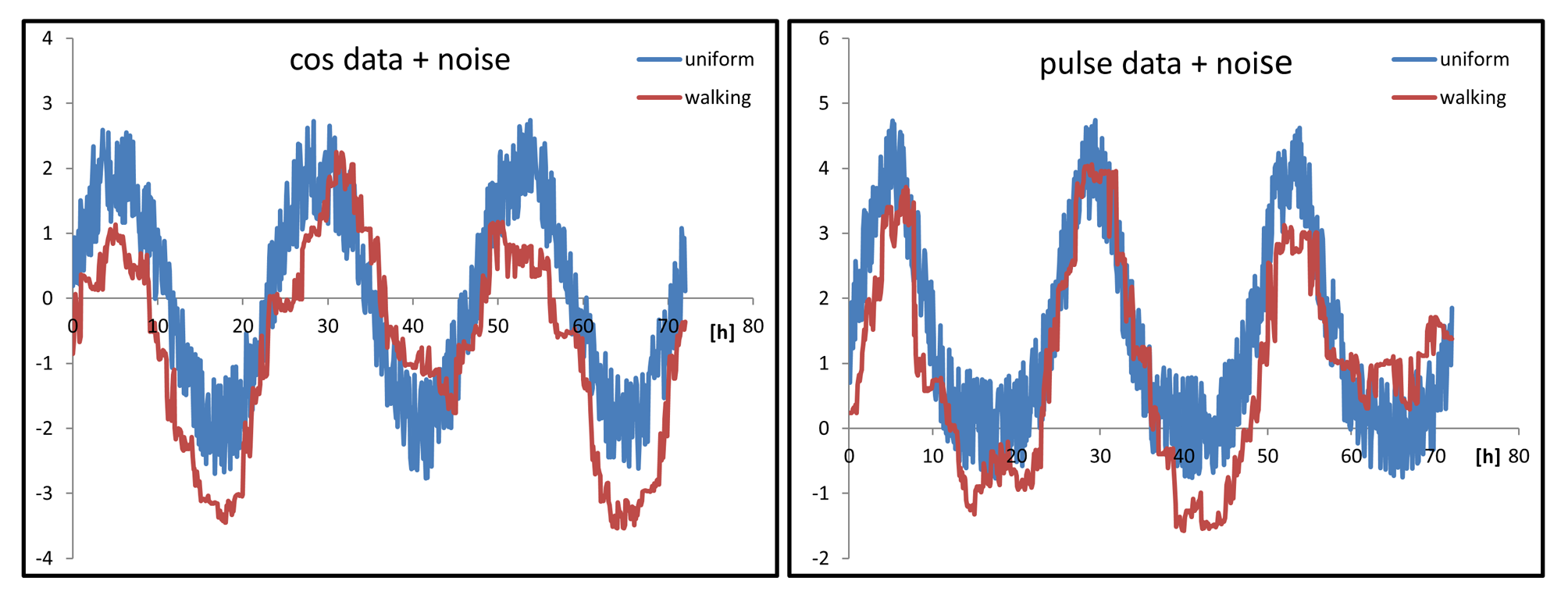

Supplement: Figure S3 — Difference between frequently sampled data with uniform and walking noise added. (TIF) [file pone.0096462.s003.tif]

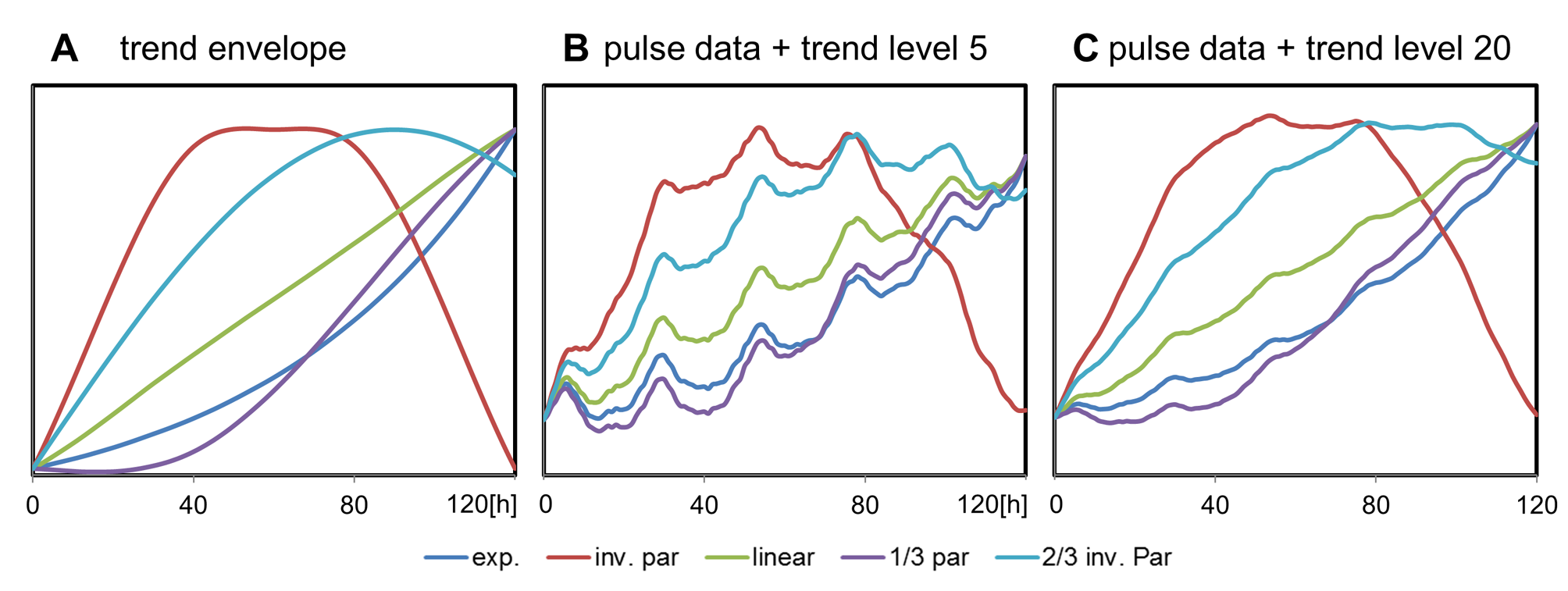

Supplement: Figure S4 — Shapes of baseline trends and examples of data with baseline trends applied. A) Shapes of trend envelopes, B - C) data with trends applied. The trend shapes: exp: exponential; linear; inv. par: inverse parabola; 2/3 inv. par: 2/3 inverse parabola; and 1/3 par: 1/3 parabola. (TIF) [file pone.0096462.s004.tif]

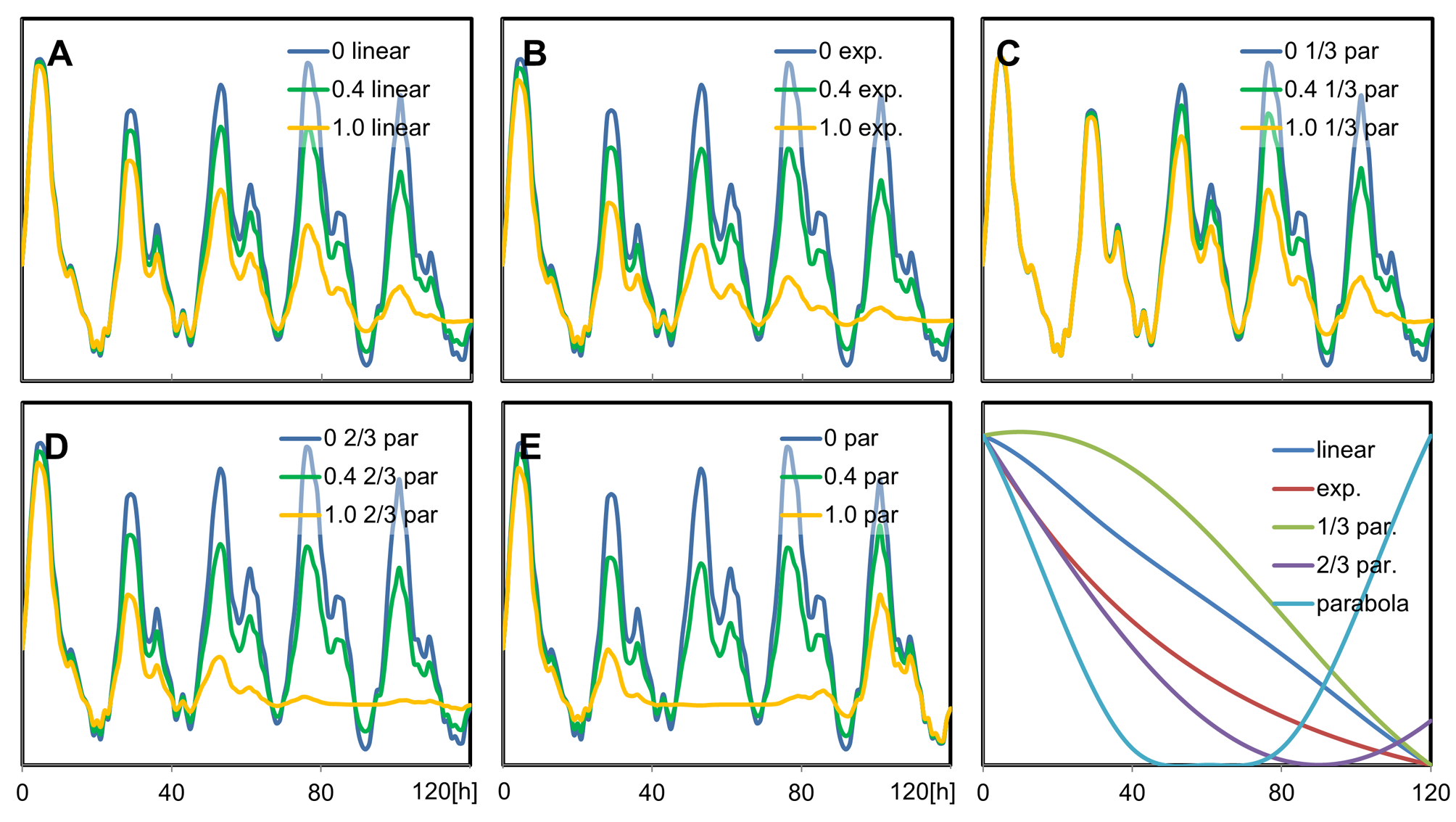

Supplement: Figure S5 — Shapes of amplitude trends envelopes and examples of data modified by them. A-E) Data with trends applied, the trend shape and its levels are indicated on the graph. F) Shapes of trend envelopes. (TIF) [file pone.0096462.s005.tif]

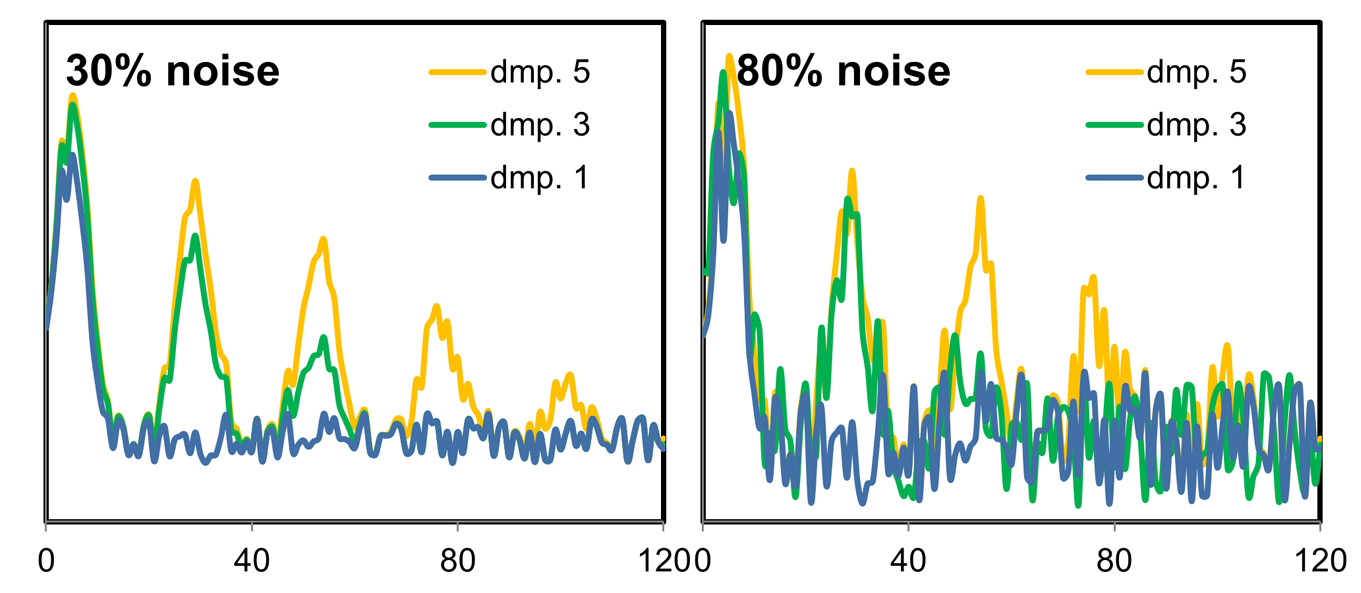

Supplement: Figure S6 — Data used for arhythmicity test. (TIF) [file pone.0096462.s006.tif]

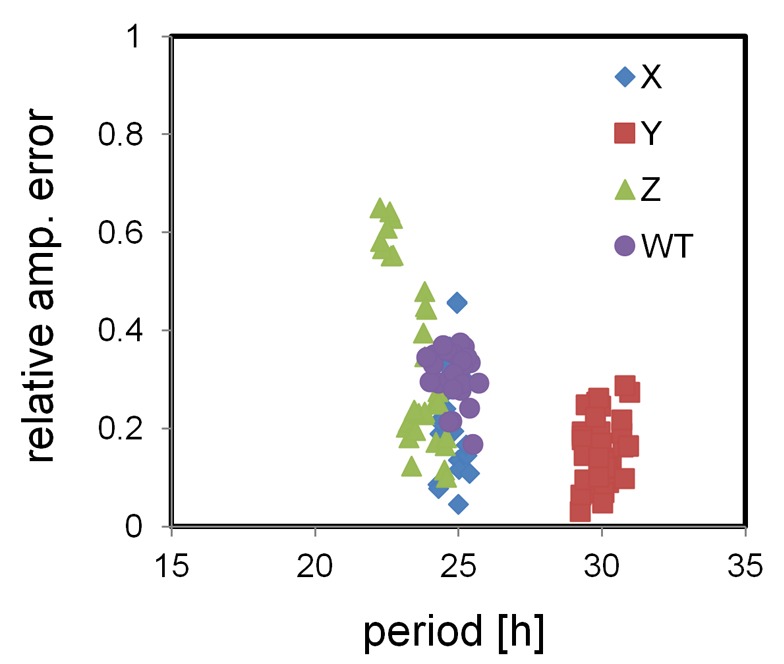

Supplement: Figure S7 — Example of RAE plot for period analysis of WT and 3 mutants. (TIF) [file pone.0096462.s007.tif]
