# Supplementary material for: Strengths and Limitations of Period Estimation Methods for Circadian Data
Source: PLoS One. 2014 May 8;9(5):e96462. doi: 10.1371/journal.pone.0096462 (PMC4014635; doi:10.1371/journal.pone.0096462)
Supplement: Table S1 — Impact of noise level on mean period. (DOCX) [file pone.0096462.s008.docx]

Table S1a. Impact of uniform noise level on period estimates.

| Shape^1^ | Method | NL 30%^2^ | NL 80%^2^ | NL 160%^2^ | NL 300%^2^ |
| --- | --- | --- | --- | --- | --- |
| cos | EPR | 23.6 (0.08) | 23.65 (0.26) | 23.8 (0.8) | 24.76 (2.05) |
| cos | MFF | 23.87 (0.07) | 23.88 (0.24) | 23.88 (0.49) | 23.99 (1.08)+ |
| cos | NLLS | 23.96 (0.08) | 23.91 (0.18) | 23.89 (0.36) | 23.96 (0.75)+ |
| cos | MESA | 23.99 (0.08) | 23.94 (0.22) | 23.9 (0.55) | 24.01 (1.26)+ |
| cos | LSPR | 23.86 (0.07) | 23.87 (0.18) | 23.9 (0.37) | 23.96 (0.74)+ |
| cos | SR | 24.26 (0.07) | 24.25 (0.19) | 24.24 (0.38) | 24.23 (0.74) |
| pul | EPR | 23.63 (0.07) | 23.65 (0.21) | 23.84 (0.72) | 24.91 (2.21) |
| pul | MFF | 23.88 (0.07) | 23.86 (0.22) | 23.81 (0.46) | 24.04 (1.11)+ |
| pul | NLLS | 23.94 (0.08) | 23.93 (0.19) | 23.96 (0.41)+ | 23.97 (1.37)+ |
| pul | MESA | 23.98 (0.08) | 23.93 (0.22) | 23.89 (0.55) | 24.13 (1.52)+ |
| pul | LSPR | 23.95 (0.07) | 23.96 (0.19) | 23.99 (0.4)+ | 24.07 (0.81)+ |
| pul | SR | 24.29 (0.08) | 24.29 (0.2) | 24.28 (0.4) | 24.18 (1.35)+ |
| dblp | EPR | 23.88 (0.07) | 23.88 (0.22) | 24.05 (0.69)+ | 25.63 (2.73) |
| dblp | MFF | 23.94 (0.06) | 23.97 (0.21) | 24.05 (0.45)+ | 24.49 (1.78) |
| dblp | NLLS | 23.88 (0.13) | 23.88 (0.26) | 24.0 (0.58)+ | 24.14 (1.9)+ |
| dblp | MESA | 23.98 (0.1) | 23.93 (0.27) | 23.97 (0.71)+ | 24.3 (1.95) |
| dblp | LSPR | 24.21 (0.09) | 24.23 (0.25) | 24.27 (0.51) | 24.41 (1.1) |
| dblp | SR | 24.55 (0.1) | 24.55 (0.25) | 24.45 (1.2) | 23.98 (2.73)+ |
| shl | EPR | 24.24 (0.09) | 24.27 (0.23) | 24.4 (0.51) | 24.46 (0.95) |
| shl | MFF | 24.0 (0.06)+ | 24.0 (0.14)+ | 24.0 (0.35)+ | 23.98 (0.76)+ |
| shl | NLLS | 23.94 (0.07) | 23.94 (0.18) | 23.97 (0.37)+ | 24.03 (0.67)+ |
| shl | MESA | 24.01 (0.08)+ | 24.01 (0.19)+ | 23.99 (0.37)+ | 23.97 (0.75)+ |
| shl | LSPR | 24.11 (0.06) | 24.11 (0.16) | 24.1 (0.33) | 24.08 (0.62)+ |
| shl | SR | 24.21 (0.09) | 24.2 (0.24) | 24.19 (0.49) | 24.01 (1.59)+ |
| asym | EPR | 24.4 (0.35) | 24.58 (0.55) | 24.86 (0.82) | 25.89 (2.12) |
| asym | MFF | 23.96 (0.08) | 24.06 (0.19)+ | 24.19 (0.36) | 24.51 (1.14) |
| asym | NLLS | 24.14 (0.11) | 24.25 (0.27) | 24.48 (0.54) | 24.51 (1.48) |
| asym | MESA | 24.07 (0.1)+ | 24.07 (0.26)+ | 24.16 (0.57)+ | 24.37 (1.33) |
| asym | LSPR | 24.56 (0.09) | 24.56 (0.23) | 24.59 (0.47) | 24.64 (0.91) |
| asym | SR | 24.52 (0.1) | 24.51 (0.27) | 24.5 (0.55) | 24.15 (4.03)+ |
| all | EPR | 23.95 (0.36) | 24.0 (0.49)+ | 24.19 (0.82) | 25.13 (2.16) |
| all | MFF | 23.93 (0.08) | 23.95 (0.22) | 23.98 (0.45)+ | 24.2 (1.24) |
| all | NLLS | 23.98 (0.13) | 23.98 (0.26) | 24.06 (0.5) | 24.12 (1.33) |
| all | MESA | 24.0 (0.1)+ | 23.98 (0.24) | 23.98 (0.57)+ | 24.16 (1.42) |
| all | LSPR | 24.14 (0.25) | 24.15 (0.32) | 24.17 (0.48) | 24.23 (0.89) |
| all | SR | 24.37 (0.17) | 24.36 (0.27) | 24.33 (0.69) | 24.11 (2.39)+ |

Data sets with different noise level were analysed using all the methods. The mean period value is reported in the table (standard deviation is given in brackets). Data sets were created by adding noise of specific level to the hourly-sampled template of 3 days duration. 1) The base shape of the signal: cosine (cos), pulse (pul); double pulse (dpl); shoulder (shl) and moderate asymmetry (asym), (all) represents aggregated results from all the signals. 2) NL- noise level as the percentage of the original signal amplitude. +) Means which are accurate, not statistically different from the expected period value, are marked with +. The underlying period was 24.08h for asym data and 24.00h for the other signals.

Table S1b. Impact of walking noise level on period estimates.

| Shape^1^ | Method | NL 30%^2^ | NL 80%^2^ | NL 160%^2^ | NL 300%^2^ |
| --- | --- | --- | --- | --- | --- |
| cos | EPR | 23.6 (0.11) | 23.6 (0.27) | 23.8 (1.14) | 24.99 (2.86) |
| cos | MFF | 23.85 (0.11) | 23.84 (0.32) | 23.83 (0.61) | 24.77 (2.72) |
| cos | NLLS | 23.94 (0.12) | 23.85 (0.3) | 23.78 (0.6) | 24.31 (3.51)+ |
| cos | MESA | 23.98 (0.14)+ | 23.95 (0.73)+ | 23.82 (1.05) | 23.83 (2.22)+ |
| cos | LSPR | 23.85 (0.11) | 23.83 (0.28) | 23.82 (0.56) | 24.37 (2.82)+ |
| cos | SR | 24.25 (0.12) | 24.25 (0.32) | 24.31 (0.7) | 26.98 (6.94) |
| pul | EPR | 23.64 (0.08) | 23.64 (0.21) | 23.86 (1.09)+ | 25.2 (2.8) |
| pul | MFF | 23.87 (0.09) | 23.86 (0.27) | 23.85 (0.54) | 24.92 (2.7) |
| pul | NLLS | 23.92 (0.13) | 23.83 (0.33) | 23.81 (0.63) | 24.62 (3.62) |
| pul | MESA | 23.97 (0.13) | 23.9 (0.96)+ | 23.74 (1.16) | 23.74 (2.08)+ |
| pul | LSPR | 23.93 (0.12) | 23.92 (0.3) | 23.92 (0.6)+ | 24.56 (2.93) |
| pul | SR | 24.29 (0.13) | 24.29 (0.34) | 24.37 (0.74) | 27.68 (7.79) |
| dblp | EPR | 23.9 (0.08) | 23.93 (0.2) | 24.34 (1.37) | 26.36 (3.49) |
| dblp | MFF | 23.96 (0.08) | 23.99 (0.2)+ | 24.19 (0.99) | 25.99 (3.66) |
| dblp | NLLS | 23.91 (0.15) | 23.85 (0.41) | 23.94 (1.47)+ | 25.15 (4.74) |
| dblp | MESA | 23.97 (0.17) | 23.91 (1.05)+ | 23.88 (1.46)+ | 24.59 (3.34) |
| dblp | LSPR | 24.21 (0.15) | 24.24 (0.43) | 24.34 (1.46) | 25.6 (3.72) |
| dblp | SR | 24.56 (0.17) | 24.61 (0.45) | 25.02 (2.99) | 30.47 (10.12) |
| shl | EPR | 24.21 (0.11) | 24.2 (0.26) | 24.21 (0.48) | 25.27 (3.21) |
| shl | MFF | 23.98 (0.08) | 23.95 (0.23) | 23.89 (0.46) | 24.25 (2.19)+ |
| shl | NLLS | 23.9 (0.14) | 23.84 (0.36) | 23.82 (0.71) | 24.1 (2.12)+ |
| shl | MESA | 24.01 (0.14)+ | 23.98 (0.39)+ | 23.96 (0.8)+ | 24.11 (2.1)+ |
| shl | LSPR | 24.08 (0.13) | 24.02 (0.33)+ | 23.95 (0.63)+ | 24.15 (2.23)+ |
| shl | SR | 24.18 (0.17) | 24.15 (0.46) | 24.16 (0.95) | 27.24 (8.12) |
| asym | EPR | 24.35 (0.37) | 24.46 (0.58) | 24.68 (1.17) | 25.98 (3.19) |
| asym | MFF | 23.95 (0.05) | 24.02 (0.2) | 24.1 (0.53)+ | 25.26 (2.97) |
| asym | NLLS | 24.1 (0.17)+ | 24.07 (0.44)+ | 24.19 (0.88)+ | 25.43 (4.6) |
| asym | MESA | 24.05 (0.17) | 23.93 (0.47) | 23.95 (1.06)+ | 24.63 (3.16) |
| asym | LSPR | 24.52 (0.15) | 24.48 (0.37) | 24.44 (0.73) | 25.69 (3.48) |
| asym | SR | 24.51 (0.18) | 24.49 (0.48) | 24.72 (1.33) | 29.32 (9.55) |
| all | EPR | 23.94 (0.35) | 23.97 (0.47) | 24.18 (1.14) | 25.56 (3.15) |
| all | MFF | 23.92 (0.1) | 23.93 (0.26) | 23.97 (0.67)+ | 25.04 (2.94) |
| all | NLLS | 23.95 (0.16) | 23.89 (0.38) | 23.91 (0.93) | 24.72 (3.86) |
| all | MESA | 24.0 (0.15)+ | 23.93 (0.76) | 23.87 (1.13) | 24.18 (2.66) |
| all | LSPR | 24.12 (0.27) | 24.1 (0.42) | 24.09 (0.9) | 24.87 (3.14) |
| all | SR | 24.36 (0.21) | 24.36 (0.45) | 24.52 (1.62) | 28.34 (8.66) |

Data sets with different noise level were analysed using all the methods. The mean period value is reported in the table (standard deviation is given in brackets). Data sets were created by adding noise of specific level to the hourly-sampled template of 3 days duration. 1) The base shape of the signal: cosine (cos), pulse (pul); double pulse (dpl); shoulder (shl) and moderate asymmetry (asym), (all) represents aggregated results from all the signals. 2) NL- noise level as the percentage of the original signal amplitude. +) Means which are accurate, not statistically different from the expected period value, are marked with +. The underlying period was 24.08h for asym data and 24.00h for the other signals.
