# Supplementary material for: Strengths and Limitations of Period Estimation Methods for Circadian Data
Source: PLoS One. 2014 May 8;9(5):e96462. doi: 10.1371/journal.pone.0096462 (PMC4014635; doi:10.1371/journal.pone.0096462)
Supplement: Table S2 — Impact of noise level on absolute error. (DOCX) [file pone.0096462.s009.docx]

Table S2a. Impact of uniform noise level on absolute error.

| Shape^1^ | Method | NL 30%^2^ | NL 80%^2^ | NL 160%^2^ | NL 300%^2^ |
| --- | --- | --- | --- | --- | --- |
| cos | EPR | 0.4 | 0.38 | 0.61 | 1.6 |
| cos | MFF | 0.14 | 0.21 | 0.44 | 0.8 |
| cos | NLLS | 0.07 | 0.16 | 0.3 | 0.58 |
| cos | MESA | 0.06 | 0.17 | 0.41 | 0.94 |
| cos | LSPR | 0.14 | 0.18 | 0.31 | 0.58 |
| cos | SR | 0.26 | 0.26 | 0.35 | 0.6 |
| pul | EPR | 0.37 | 0.36 | 0.54 | 1.65 |
| pul | MFF | 0.12 | 0.21 | 0.43 | 0.8 |
| pul | NLLS | 0.08 | 0.17 | 0.33 | 0.72 |
| pul | MESA | 0.07 | 0.18 | 0.43 | 1.04 |
| pul | LSPR | 0.07 | 0.16 | 0.32 | 0.61 |
| pul | SR | 0.29 | 0.3 | 0.38 | 0.75 |
| dblp | EPR | 0.12 | 0.2 | 0.49 | 2.16 |
| dblp | MFF | 0.07 | 0.17 | 0.37 | 1.1 |
| dblp | NLLS | 0.14 | 0.23 | 0.47 | 1.03 |
| dblp | MESA | 0.08 | 0.23 | 0.55 | 1.36 |
| dblp | LSPR | 0.21 | 0.27 | 0.46 | 0.86 |
| dblp | SR | 0.55 | 0.55 | 0.71 | 1.36 |
| shl | EPR | 0.24 | 0.28 | 0.5 | 0.86 |
| shl | MFF | 0.05 | 0.12 | 0.26 | 0.6 |
| shl | NLLS | 0.07 | 0.16 | 0.3 | 0.54 |
| shl | MESA | 0.06 | 0.16 | 0.3 | 0.59 |
| shl | LSPR | 0.11 | 0.15 | 0.27 | 0.51 |
| shl | SR | 0.21 | 0.25 | 0.42 | 0.94 |
| asym | EPR | 0.33 | 0.55 | 0.88 | 2.02 |
| asym | MFF | 0.14 | 0.17 | 0.31 | 0.82 |
| asym | NLLS | 0.1 | 0.25 | 0.52 | 0.91 |
| asym | MESA | 0.08 | 0.21 | 0.45 | 1.03 |
| asym | LSPR | 0.48 | 0.48 | 0.54 | 0.8 |
| asym | SR | 0.44 | 0.44 | 0.54 | 1.66 |
| all | EPR | 0.29 | 0.35 | 0.61 | 1.66 |
| all | MFF | 0.1 | 0.18 | 0.36 | 0.82 |
| all | NLLS | 0.09 | 0.19 | 0.38 | 0.76 |
| all | MESA | 0.07 | 0.19 | 0.43 | 0.99 |
| all | LSPR | 0.2 | 0.25 | 0.38 | 0.67 |
| all | SR | 0.35 | 0.36 | 0.48 | 1.06 |

Table S2b. Impact of walking noise level on absolute error.

| Shape^1^ | Method | NL 30%^2^ | NL 80%^2^ | NL 160%^2^ | NL 300%^2^ |
| --- | --- | --- | --- | --- | --- |
| cos | EPR | 0.4 | 0.42 | 0.7 | 1.93 |
| cos | MFF | 0.16 | 0.28 | 0.51 | 1.54 |
| cos | NLLS | 0.11 | 0.26 | 0.5 | 1.44 |
| cos | MESA | 0.11 | 0.33 | 0.67 | 1.36 |
| cos | LSPR | 0.16 | 0.26 | 0.45 | 1.43 |
| cos | SR | 0.25 | 0.33 | 0.56 | 3.37 |
| pul | EPR | 0.36 | 0.37 | 0.63 | 1.87 |
| pul | MFF | 0.14 | 0.23 | 0.43 | 1.58 |
| pul | NLLS | 0.12 | 0.29 | 0.5 | 1.66 |
| pul | MESA | 0.11 | 0.39 | 0.77 | 1.37 |
| pul | LSPR | 0.1 | 0.24 | 0.46 | 1.57 |
| pul | SR | 0.29 | 0.36 | 0.61 | 4.07 |
| dblp | EPR | 0.11 | 0.17 | 0.69 | 2.71 |
| dblp | MFF | 0.07 | 0.16 | 0.5 | 2.53 |
| dblp | NLLS | 0.14 | 0.34 | 0.88 | 2.23 |
| dblp | MESA | 0.13 | 0.49 | 0.95 | 2.16 |
| dblp | LSPR | 0.22 | 0.38 | 0.89 | 2.44 |
| dblp | SR | 0.56 | 0.64 | 1.46 | 7.21 |
| shl | EPR | 0.21 | 0.23 | 0.36 | 1.75 |
| shl | MFF | 0.07 | 0.17 | 0.37 | 1.16 |
| shl | NLLS | 0.14 | 0.32 | 0.59 | 1.27 |
| shl | MESA | 0.12 | 0.31 | 0.63 | 1.35 |
| shl | LSPR | 0.12 | 0.26 | 0.51 | 1.27 |
| shl | SR | 0.21 | 0.39 | 0.79 | 4.58 |
| asym | EPR | 0.3 | 0.5 | 0.86 | 2.33 |
| asym | MFF | 0.14 | 0.19 | 0.39 | 1.72 |
| asym | NLLS | 0.13 | 0.34 | 0.71 | 2.32 |
| asym | MESA | 0.13 | 0.34 | 0.74 | 1.93 |
| asym | LSPR | 0.44 | 0.46 | 0.67 | 2.21 |
| asym | SR | 0.43 | 0.52 | 1.02 | 6.02 |
| all | EPR | 0.28 | 0.34 | 0.65 | 2.12 |
| all | MFF | 0.12 | 0.21 | 0.44 | 1.71 |
| all | NLLS | 0.13 | 0.31 | 0.64 | 1.79 |
| all | MESA | 0.12 | 0.37 | 0.75 | 1.64 |
| all | LSPR | 0.21 | 0.32 | 0.6 | 1.78 |
| all | SR | 0.35 | 0.45 | 0.89 | 5.05 |

Data sets with noise level were analysed using all the methods and the average absolute error is reported in the table. The absolute error is defined as the absolute value of the difference between calculated period and the expected value (24.08 for asym signal and 24h for the others). Data sets were created by adding noise of given level to the hourly-sampled templates of 3 days duration. 1) The base shape of the signal: cosine (cos), pulse (pul); double pulse (dpl); shoulder (shl) and moderate asymmetry (asym), (all) represents aggregated results from all the sets2) NL- noise level as the percentage of the original signal amplitude.
