# Supplementary material for: Strengths and Limitations of Period Estimation Methods for Circadian Data
Source: PLoS One. 2014 May 8;9(5):e96462. doi: 10.1371/journal.pone.0096462 (PMC4014635; doi:10.1371/journal.pone.0096462)
Supplement: Table S3 — Impact of data duration on mean period. (DOCX) [file pone.0096462.s010.docx]

Table S3. Impact of data duration on period estimates (walking noise data set).

| Shape | Method | 3 | 5 | 10 |
| --- | --- | --- | --- | --- |
| cos | EPR | 23.97 (1.29)+ | 23.9 (0.3) | 23.96 (0.1) |
| cos | MFF | 23.92 (0.73)+ | 23.97 (0.29)+ | 23.99 (0.09)+ |
| cos | NLLS | 23.89 (0.7) | 23.94 (0.31) | 23.99 (0.09)+ |
| cos | MESA | 23.72 (0.79) | 23.91 (0.3) | 23.97 (0.15) |
| cos | LSPR | 23.9 (0.71)+ | 23.99 (0.28)+ | 23.99 (0.09)+ |
| cos | SR | 24.39 (0.69) | 24.12 (0.36) | 24.0 (0.11)+ |
| pul | EPR | 23.84 (0.85) | 23.89 (0.23) | 23.96 (0.08) |
| pul | MFF | 23.88 (0.58) | 23.95 (0.25) | 23.98 (0.08) |
| pul | NLLS | 23.81 (0.7) | 23.96 (0.34)+ | 23.98 (0.1) |
| pul | MESA | 23.7 (1.31) | 23.85 (0.34) | 23.91 (0.16) |
| pul | LSPR | 23.96 (0.69)+ | 24.02 (0.31)+ | 23.99 (0.1)+ |
| pul | SR | 24.42 (0.72) | 24.14 (0.37) | 24.0 (0.12)+ |
| dblp | EPR | 24.13 (1.23)+ | 23.97 (0.2)+ | 23.99 (0.06) |
| dblp | MFF | 24.01 (1.09)+ | 24.02 (0.23)+ | 24.0 (0.06)+ |
| dblp | NLLS | 23.76 (1.0) | 23.96 (0.4)+ | 23.99 (0.12)+ |
| dblp | MESA | 23.75 (1.48) | 23.78 (0.44) | 23.86 (0.24) |
| dblp | LSPR | 24.3 (1.22) | 24.13 (0.41) | 24.03 (0.11) |
| dblp | SR | 24.92 (1.61) | 24.25 (0.48) | 24.03 (0.3)+ |
| shl | EPR | 24.38 (0.52) | 24.12 (0.23) | 24.03 (0.07) |
| shl | MFF | 24.03 (0.51)+ | 24.0 (0.21)+ | 24.0 (0.07)+ |
| shl | NLLS | 23.93 (0.7)+ | 23.98 (0.33)+ | 24.0 (0.11)+ |
| shl | MESA | 23.85 (0.65) | 23.87 (0.33) | 23.92 (0.15) |
| shl | LSPR | 24.11 (0.65) | 24.04 (0.32)+ | 24.01 (0.1)+ |
| shl | SR | 24.28 (0.91) | 24.11 (0.33) | 23.99 (0.11)+ |
| asym | EPR | 24.82 (1.08) | 24.18 (0.24) | 24.08 (0.06)+ |
| asym | MFF | 24.25 (0.57) | 24.13 (0.21) | 24.08 (0.07)+ |
| asym | NLLS | 24.34 (0.85) | 24.2 (0.37) | 24.09 (0.11)+ |
| asym | MESA | 23.97 (0.86)+ | 24.01 (0.38) | 24.03 (0.18) |
| asym | LSPR | 24.68 (0.78) | 24.32 (0.34) | 24.13 (0.1) |
| asym | SR | 24.83 (1.14) | 24.29 (0.39) | 24.07 (0.13)+ |
| all | EPR | 24.23 (1.08) | 24.01 (0.27)+ | 24.0 (0.09)+ |
| all | MFF | 24.02 (0.74)+ | 24.01 (0.25)+ | 24.01 (0.08) |
| all | NLLS | 23.95 (0.82)+ | 24.01 (0.36)+ | 24.01 (0.12) |
| all | MESA | 23.8 (1.07) | 23.89 (0.37) | 23.94 (0.19) |
| all | LSPR | 24.19 (0.88) | 24.1 (0.35) | 24.03 (0.11) |
| all | SR | 24.57 (1.1) | 24.18 (0.4) | 24.02 (0.17) |

Data sets with different signal duration were analysed using all the methods. The mean period value is reported in the table (standard deviation is given in brackets). Data sets were created by adding walking noise of 160% of the original signal amplitude to the hourly-sampled templates of different length. 1) The base shape of the signal: cosine (cos), pulse (pul); double pulse (dpl); shoulder (shl) and moderate asymmetry (asym), (all) represents aggregated results from all the signals. +) Means which are accurate, not statistically different from the expected period value, are marked with +. The underlying period was 24.08h for asym data and 24.00h for the other signals.
