# Supplementary material for: Strengths and Limitations of Period Estimation Methods for Circadian Data
Source: PLoS One. 2014 May 8;9(5):e96462. doi: 10.1371/journal.pone.0096462 (PMC4014635; doi:10.1371/journal.pone.0096462)
Supplement: Table S4 — Impact of data duration on absolute error. (DOCX) [file pone.0096462.s011.docx]

Table S4. Impact of data duration on absolute error (walking noise data set).

| Shape | Method | 3 | 5 | 10 |
| --- | --- | --- | --- | --- |
| cos | EPR | 0.83 | 0.25 | 0.08 |
| cos | MFF | 0.6 | 0.25 | 0.07 |
| cos | NLLS | 0.55 | 0.25 | 0.07 |
| cos | MESA | 0.63 | 0.25 | 0.12 |
| cos | LSPR | 0.57 | 0.23 | 0.07 |
| cos | SR | 0.63 | 0.31 | 0.09 |
| pul | EPR | 0.6 | 0.2 | 0.06 |
| pul | MFF | 0.48 | 0.21 | 0.06 |
| pul | NLLS | 0.59 | 0.27 | 0.08 |
| pul | MESA | 0.74 | 0.29 | 0.15 |
| pul | LSPR | 0.55 | 0.24 | 0.08 |
| pul | SR | 0.67 | 0.3 | 0.09 |
| dblp | EPR | 0.64 | 0.16 | 0.04 |
| dblp | MFF | 0.56 | 0.19 | 0.04 |
| dblp | NLLS | 0.81 | 0.32 | 0.09 |
| dblp | MESA | 0.91 | 0.39 | 0.21 |
| dblp | LSPR | 0.86 | 0.35 | 0.09 |
| dblp | SR | 1.17 | 0.44 | 0.13 |
| shl | EPR | 0.49 | 0.19 | 0.05 |
| shl | MFF | 0.39 | 0.17 | 0.05 |
| shl | NLLS | 0.56 | 0.25 | 0.08 |
| shl | MESA | 0.51 | 0.26 | 0.13 |
| shl | LSPR | 0.53 | 0.26 | 0.08 |
| shl | SR | 0.74 | 0.27 | 0.09 |
| asym | EPR | 0.92 | 0.2 | 0.05 |
| asym | MFF | 0.44 | 0.18 | 0.06 |
| asym | NLLS | 0.72 | 0.3 | 0.09 |
| asym | MESA | 0.69 | 0.3 | 0.15 |
| asym | LSPR | 0.77 | 0.32 | 0.09 |
| asym | SR | 1.03 | 0.33 | 0.11 |
| all | EPR | 0.69 | 0.2 | 0.05 |
| all | MFF | 0.49 | 0.2 | 0.06 |
| all | NLLS | 0.64 | 0.28 | 0.09 |
| all | MESA | 0.7 | 0.3 | 0.15 |
| all | LSPR | 0.65 | 0.28 | 0.08 |
| all | SR | 0.85 | 0.33 | 0.1 |

Data sets with different signal duration were analysed using all the methods and the average absolute error is reported in the table. The absolute error is defined as the absolute value of the difference between calculated period and the expected value (24.08 for asym signal and 24h for the others). Data sets were created by adding walking noise of 160% of the original signal amplitude to the hourly-sampled templates of different duration. 1) The base shape of the signal: cosine (cos), pulse (pul); double pulse (dpl); shoulder (shl) and moderate asymmetry (asym), (all) represents aggregated results from all the sets.
