# Supplementary material for: Strengths and Limitations of Period Estimation Methods for Circadian Data
Source: PLoS One. 2014 May 8;9(5):e96462. doi: 10.1371/journal.pone.0096462 (PMC4014635; doi:10.1371/journal.pone.0096462)
Supplement: Table S5 — Impact of sampling frequency on mean period. (DOCX) [file pone.0096462.s012.docx]

Table S5a. Impact of data sampling frequency on period estimates (uniform noise data set).

| Shape | Met. | (3.0) 0.1 | (3.0) 1.0 | (5.0) 0.1 | (5.0) 1.0 | (5.0) 2.0 | (9.99) 2.0 |
| --- | --- | --- | --- | --- | --- | --- | --- |
| cos | EPR | 23.61 (0.15) | 23.65 (0.24) | 23.87 (0.07) | 23.88 (0.09) | 23.89 (0.13) | 23.98 (0.05) |
| cos | MFF | 23.96 (0.08) | 23.88 (0.24) | 23.99 (0.03) | 23.99 (0.08)+ | 23.97 (0.14) | 24.0 (0.04)+ |
| cos | NLLS | 23.96 (0.08) | 23.92 (0.19) | 23.98 (0.03) | 23.99 (0.08) | 23.97 (0.11) | 24.0 (0.04)+ |
| cos | MESA | 23.99 (0.07)+ | 23.98 (0.2)+ | 23.99 (0.03) | 24.0 (0.08)+ | 23.98 (0.13)+ | 23.99 (0.05)+ |
| cos | LSPR | 23.9 (0.06) | 23.88 (0.18) | 23.99 (0.02) | 23.99 (0.08) | 23.98 (0.11) | 24.0 (0.04)+ |
| cos | SR | 24.24 (0.09) | 24.28 (0.18) | 24.11 (0.04) | 24.1 (0.09) | 24.08 (0.13) | 24.01 (0.04) |
| pul | EPR | 23.65 (0.14) | 23.66 (0.21) | 23.88 (0.06) | 23.87 (0.09) | 23.89 (0.12) | 23.98 (0.05) |
| pul | MFF | 23.97 (0.07) | 23.82 (0.24) | 24.0 (0.03)+ | 23.97 (0.08) | 23.95 (0.14) | 24.0 (0.04)+ |
| pul | NLLS | 24.0 (0.08)+ | 23.89 (0.19) | 23.97 (0.03) | 23.97 (0.08) | 23.96 (0.12) | 24.0 (0.04)+ |
| pul | MESA | 24.0 (0.07)+ | 23.92 (0.2) | 23.99 (0.03) | 23.97 (0.09) | 23.95 (0.13) | 23.97 (0.05) |
| pul | LSPR | 24.02 (0.07) | 23.93 (0.19) | 24.03 (0.03) | 24.01 (0.08)+ | 23.99 (0.12)+ | 24.0 (0.04)+ |
| pul | SR | 24.29 (0.09) | 24.28 (0.18) | 24.12 (0.05) | 24.1 (0.1) | 24.09 (0.14) | 24.0 (0.04)+ |
| dblp | EPR | 23.9 (0.13) | 23.87 (0.23) | 23.96 (0.07) | 23.97 (0.09) | 23.96 (0.1) | 23.99 (0.04) |
| dblp | MFF | 23.98 (0.06) | 23.96 (0.24) | 24.0 (0.02)+ | 24.01 (0.08)+ | 24.0 (0.13)+ | 24.01 (0.04) |
| dblp | NLLS | 23.9 (0.12) | 23.87 (0.3) | 23.96 (0.03) | 23.96 (0.11) | 23.95 (0.15) | 24.0 (0.05)+ |
| dblp | MESA | 23.98 (0.09) | 23.93 (0.3) | 23.99 (0.04)+ | 23.97 (0.12) | 23.97 (0.18) | 23.99 (0.08) |
| dblp | LSPR | 24.31 (0.1) | 24.2 (0.3) | 24.12 (0.03) | 24.09 (0.11) | 24.08 (0.15) | 24.03 (0.05) |
| dblp | SR | 24.52 (0.13) | 24.56 (0.27) | 24.16 (0.06) | 24.14 (0.13) | 24.13 (0.18) | 24.0 (0.05)+ |
| shl | EPR | 24.3 (0.15) | 24.3 (0.25) | 24.09 (0.07) | 24.09 (0.09) | 24.11 (0.11) | 24.02 (0.04) |
| shl | MFF | 24.01 (0.05)+ | 24.03 (0.16) | 24.0 (0.03) | 24.01 (0.08)+ | 24.02 (0.1) | 24.0 (0.04)+ |
| shl | NLLS | 23.99 (0.06) | 23.98 (0.19)+ | 23.99 (0.02) | 23.99 (0.09)+ | 24.02 (0.13)+ | 24.0 (0.04)+ |
| shl | MESA | 24.01 (0.07)+ | 24.06 (0.21) | 24.0 (0.03)+ | 24.0 (0.09)+ | 24.04 (0.14) | 24.0 (0.06)+ |
| shl | LSPR | 24.1 (0.05) | 24.14 (0.17) | 24.04 (0.02) | 24.05 (0.08) | 24.08 (0.12) | 24.02 (0.04) |
| shl | SR | 24.31 (0.11) | 24.26 (0.24) | 24.07 (0.04) | 24.1 (0.1) | 24.15 (0.14) | 23.99 (0.05)+ |
| asym | EPR | 24.62 (0.39) | 24.62 (0.58) | 24.09 (0.06) | 24.1 (0.09) | 24.11 (0.11) | 24.07 (0.04)+ |
| asym | MFF | 24.1 (0.07) | 24.02 (0.19) | 24.1 (0.02) | 24.07 (0.1)+ | 24.06 (0.12) | 24.08 (0.05)+ |
| asym | NLLS | 24.11 (0.1) | 24.24 (0.29) | 24.15 (0.04) | 24.13 (0.09) | 24.12 (0.14) | 24.09 (0.05)+ |
| asym | MESA | 24.07 (0.09)+ | 24.07 (0.25)+ | 24.07 (0.03) | 24.1 (0.1) | 24.09 (0.14)+ | 24.08 (0.07)+ |
| asym | LSPR | 24.64 (0.08) | 24.55 (0.23) | 24.28 (0.03) | 24.27 (0.09) | 24.23 (0.13) | 24.12 (0.05) |
| asym | SR | 24.52 (0.14) | 24.54 (0.26) | 24.22 (0.06) | 24.22 (0.12) | 24.22 (0.16) | 24.07 (0.05) |
| all | EPR | 24.02 (0.44)+ | 24.02 (0.51)+ | 23.98 (0.12) | 23.98 (0.13) | 23.99 (0.15) | 24.01 (0.06) |
| all | MFF | 24.0 (0.08)+ | 23.94 (0.23) | 24.02 (0.05) | 24.01 (0.09) | 24.0 (0.13)+ | 24.02 (0.05) |
| all | NLLS | 23.99 (0.11)+ | 23.98 (0.27) | 24.01 (0.08) | 24.01 (0.11) | 24.0 (0.14)+ | 24.02 (0.06) |
| all | MESA | 24.01 (0.09) | 23.99 (0.24)+ | 24.01 (0.04) | 24.01 (0.11) | 24.01 (0.15)+ | 24.01 (0.07) |
| all | LSPR | 24.19 (0.27) | 24.14 (0.32) | 24.09 (0.11) | 24.08 (0.14) | 24.07 (0.15) | 24.04 (0.06) |
| all | SR | 24.37 (0.17) | 24.38 (0.27) | 24.14 (0.07) | 24.13 (0.12) | 24.14 (0.16) | 24.02 (0.05) |

Table S5b. Impact of data sampling frequency on period estimates (walking noise data set).

| Shape | Met. | (3.0) 0.1 | (3.0) 1.0 | (5.0) 0.1 | (5.0) 1.0 | (5.0) 2.0 | (10) 2.0 |
| --- | --- | --- | --- | --- | --- | --- | --- |
| cos | EPR | 23.62 (0.32) | 23.63 (0.3) | 23.93 (0.13) | 23.89 (0.13) | 23.88 (0.14) | 23.97 (0.06) |
| cos | MFF | 23.96 (0.34)+ | 23.88 (0.35) | 24.02 (0.14) | 23.99 (0.14)+ | 23.97 (0.14) | 24.0 (0.05)+ |
| cos | NLLS | 23.89 (0.36) | 23.84 (0.32) | 23.98 (0.15)+ | 23.96 (0.14) | 23.96 (0.13) | 24.0 (0.05)+ |
| cos | MESA | 23.81 (0.34) | 23.87 (0.36) | 23.95 (0.15) | 23.94 (0.15) | 23.95 (0.14) | 23.99 (0.07) |
| cos | LSPR | 23.91 (0.33) | 23.87 (0.31) | 24.01 (0.14)+ | 23.99 (0.13)+ | 23.98 (0.13) | 24.0 (0.05)+ |
| cos | SR | 24.2 (0.35) | 24.25 (0.35) | 24.11 (0.15) | 24.09 (0.15) | 24.07 (0.14) | 24.01 (0.06)+ |
| pul | EPR | 23.67 (0.26) | 23.63 (0.22) | 23.93 (0.11) | 23.88 (0.11) | 23.88 (0.11) | 23.97 (0.05) |
| pul | MFF | 23.95 (0.26) | 23.84 (0.28) | 24.0 (0.11)+ | 23.98 (0.11) | 23.95 (0.12) | 24.0 (0.04)+ |
| pul | NLLS | 23.89 (0.32) | 23.81 (0.33) | 23.97 (0.15) | 23.95 (0.15) | 23.95 (0.14) | 23.99 (0.05)+ |
| pul | MESA | 23.84 (0.32) | 23.83 (0.33) | 23.94 (0.16) | 23.92 (0.15) | 23.93 (0.15) | 23.97 (0.07) |
| pul | LSPR | 24.0 (0.34)+ | 23.9 (0.33) | 24.04 (0.14) | 24.01 (0.13)+ | 23.99 (0.13)+ | 24.0 (0.05)+ |
| pul | SR | 24.26 (0.3) | 24.28 (0.3) | 24.15 (0.16) | 24.11 (0.15) | 24.1 (0.15) | 24.01 (0.05)+ |
| dblp | EPR | 23.85 (0.26) | 23.89 (0.23) | 23.97 (0.06) | 23.96 (0.09) | 23.96 (0.09) | 24.0 (0.03)+ |
| dblp | MFF | 23.92 (0.23) | 23.96 (0.24) | 23.99 (0.09) | 23.99 (0.09)+ | 23.99 (0.1)+ | 24.0 (0.03)+ |
| dblp | NLLS | 23.93 (0.48)+ | 23.88 (0.44) | 23.96 (0.18) | 23.95 (0.16) | 23.94 (0.16) | 24.0 (0.05)+ |
| dblp | MESA | 23.81 (0.46) | 23.83 (0.44) | 23.89 (0.18) | 23.91 (0.17) | 23.92 (0.18) | 23.97 (0.09) |
| dblp | LSPR | 24.34 (0.48) | 24.21 (0.44) | 24.11 (0.17) | 24.1 (0.16) | 24.08 (0.17) | 24.03 (0.05) |
| dblp | SR | 24.58 (0.48) | 24.61 (0.45) | 24.19 (0.2) | 24.17 (0.2) | 24.15 (0.2) | 24.0 (0.06)+ |
| shl | EPR | 24.42 (0.4) | 24.34 (0.34) | 24.11 (0.14) | 24.1 (0.13) | 24.1 (0.12) | 24.01 (0.04) |
| shl | MFF | 24.12 (0.25) | 24.05 (0.26) | 24.04 (0.11) | 24.01 (0.11)+ | 24.02 (0.1) | 24.0 (0.04)+ |
| shl | NLLS | 24.11 (0.41) | 23.95 (0.41)+ | 24.02 (0.18)+ | 23.98 (0.16)+ | 23.98 (0.17)+ | 23.99 (0.06)+ |
| shl | MESA | 24.01 (0.36)+ | 24.02 (0.34)+ | 24.0 (0.16)+ | 23.97 (0.16) | 23.99 (0.16)+ | 23.98 (0.07) |
| shl | LSPR | 24.22 (0.38) | 24.16 (0.38) | 24.06 (0.17) | 24.05 (0.17) | 24.06 (0.16) | 24.01 (0.06) |
| shl | SR | 24.44 (0.48) | 24.3 (0.48) | 24.11 (0.17) | 24.1 (0.17) | 24.13 (0.17) | 23.99 (0.06)+ |
| asym | EPR | 24.54 (0.67) | 24.48 (0.64) | 24.06 (0.12) | 24.1 (0.1) | 24.09 (0.1)+ | 24.08 (0.05)+ |
| asym | MFF | 24.16 (0.34) | 24.05 (0.27)+ | 24.09 (0.09)+ | 24.06 (0.1) | 24.03 (0.1) | 24.09 (0.05)+ |
| asym | NLLS | 24.11 (0.47)+ | 24.11 (0.47)+ | 24.11 (0.18) | 24.12 (0.17) | 24.12 (0.16) | 24.09 (0.06) |
| asym | MESA | 24.0 (0.41) | 24.0 (0.4) | 24.01 (0.17) | 24.02 (0.17) | 24.03 (0.17) | 24.06 (0.09) |
| asym | LSPR | 24.65 (0.48) | 24.55 (0.46) | 24.28 (0.16) | 24.28 (0.16) | 24.24 (0.16) | 24.13 (0.06) |
| asym | SR | 24.53 (0.52) | 24.57 (0.51) | 24.23 (0.19) | 24.22 (0.19) | 24.21 (0.18) | 24.07 (0.07)+ |
| all | EPR | 24.02 (0.56)+ | 23.99 (0.52)+ | 24.0 (0.14)+ | 23.98 (0.15) | 23.98 (0.15) | 24.01 (0.06) |
| all | MFF | 24.02 (0.31) | 23.96 (0.29) | 24.03 (0.11) | 24.01 (0.11)+ | 23.99 (0.12)+ | 24.02 (0.05) |
| all | NLLS | 23.98 (0.42)+ | 23.92 (0.41) | 24.01 (0.18)+ | 23.99 (0.17)+ | 23.99 (0.17)+ | 24.01 (0.07) |
| all | MESA | 23.89 (0.39) | 23.91 (0.38) | 23.96 (0.17) | 23.95 (0.16) | 23.96 (0.17) | 23.99 (0.08) |
| all | LSPR | 24.22 (0.49) | 24.14 (0.46) | 24.1 (0.18) | 24.09 (0.18) | 24.07 (0.18) | 24.03 (0.07) |
| all | SR | 24.4 (0.46) | 24.4 (0.45) | 24.16 (0.18) | 24.14 (0.18) | 24.13 (0.18) | 24.01 (0.07) |

Data sets with different time intervals and selected durations were analysed using all the methods and the mean period value is reported in the table (standard deviations are omitted for clarity). Data sets were created by adding 80% noise to the templates of different duration and time interval between points. The underlying period was 24.08h for asym data and 24.00h for the other signals. 1) The base shape of the signal: cosine (cos), pulse (pul); double pulse (dpl); shoulder (shl) and moderate asymmetry (asym), (all) represents aggregated results from all the sets. 2) the time interval (sampling frequency) in the data set and in brackets the data duration.
