# Supplementary material for: Strengths and Limitations of Period Estimation Methods for Circadian Data
Source: PLoS One. 2014 May 8;9(5):e96462. doi: 10.1371/journal.pone.0096462 (PMC4014635; doi:10.1371/journal.pone.0096462)
Supplement: Table S6 — Impact of sampling frequency on absolute error. (DOCX) [file pone.0096462.s013.docx]

Table S6a. Impact of data sampling frequency on absolute error (uniform noise data set).

| Shape | Method | (3.0) 0.1 | (3.0) 1.0 | (5.0) 0.1 | (5.0) 1.0 | (5.0) 2.0 | (10) 2.0 |
| --- | --- | --- | --- | --- | --- | --- | --- |
| cos | EPR | 0.39 | 0.38 | 0.13 | 0.13 | 0.14 | 0.03 |
| cos | MFF | 0.07 | 0.2 | 0.02 | 0.06 | 0.11 | 0.03 |
| cos | NLLS | 0.07 | 0.16 | 0.03 | 0.06 | 0.09 | 0.03 |
| cos | MESA | 0.06 | 0.15 | 0.02 | 0.07 | 0.1 | 0.04 |
| cos | LSPR | 0.1 | 0.17 | 0.02 | 0.06 | 0.09 | 0.03 |
| cos | SR | 0.24 | 0.29 | 0.11 | 0.11 | 0.12 | 0.03 |
| pul | EPR | 0.35 | 0.35 | 0.12 | 0.13 | 0.13 | 0.03 |
| pul | MFF | 0.06 | 0.23 | 0.02 | 0.07 | 0.11 | 0.03 |
| pul | NLLS | 0.06 | 0.17 | 0.03 | 0.07 | 0.1 | 0.03 |
| pul | MESA | 0.06 | 0.16 | 0.02 | 0.07 | 0.11 | 0.04 |
| pul | LSPR | 0.06 | 0.16 | 0.03 | 0.06 | 0.09 | 0.03 |
| pul | SR | 0.29 | 0.3 | 0.12 | 0.12 | 0.14 | 0.04 |
| dblp | EPR | 0.13 | 0.21 | 0.06 | 0.07 | 0.09 | 0.02 |
| dblp | MFF | 0.05 | 0.2 | 0.02 | 0.07 | 0.11 | 0.03 |
| dblp | NLLS | 0.12 | 0.26 | 0.05 | 0.09 | 0.13 | 0.04 |
| dblp | MESA | 0.07 | 0.24 | 0.03 | 0.1 | 0.14 | 0.06 |
| dblp | LSPR | 0.31 | 0.29 | 0.12 | 0.11 | 0.12 | 0.05 |
| dblp | SR | 0.52 | 0.56 | 0.16 | 0.16 | 0.17 | 0.04 |
| shl | EPR | 0.3 | 0.32 | 0.09 | 0.09 | 0.11 | 0.02 |
| shl | MFF | 0.05 | 0.14 | 0.02 | 0.07 | 0.08 | 0.03 |
| shl | NLLS | 0.05 | 0.15 | 0.02 | 0.07 | 0.1 | 0.03 |
| shl | MESA | 0.06 | 0.17 | 0.02 | 0.07 | 0.12 | 0.05 |
| shl | LSPR | 0.1 | 0.17 | 0.04 | 0.08 | 0.11 | 0.03 |
| shl | SR | 0.31 | 0.29 | 0.07 | 0.12 | 0.17 | 0.04 |
| asym | EPR | 0.54 | 0.62 | 0.05 | 0.07 | 0.09 | 0.04 |
| asym | MFF | 0.06 | 0.18 | 0.03 | 0.08 | 0.11 | 0.04 |
| asym | NLLS | 0.08 | 0.25 | 0.07 | 0.09 | 0.11 | 0.03 |
| asym | MESA | 0.08 | 0.21 | 0.03 | 0.09 | 0.11 | 0.05 |
| asym | LSPR | 0.56 | 0.47 | 0.2 | 0.2 | 0.17 | 0.05 |
| asym | SR | 0.44 | 0.46 | 0.14 | 0.16 | 0.17 | 0.04 |
| all | EPR | 0.34 | 0.38 | 0.09 | 0.1 | 0.11 | 0.03 |
| all | MFF | 0.06 | 0.19 | 0.02 | 0.07 | 0.1 | 0.03 |
| all | NLLS | 0.08 | 0.2 | 0.04 | 0.08 | 0.11 | 0.03 |
| all | MESA | 0.06 | 0.19 | 0.02 | 0.08 | 0.12 | 0.05 |
| all | LSPR | 0.22 | 0.25 | 0.08 | 0.1 | 0.12 | 0.04 |
| all | SR | 0.36 | 0.38 | 0.12 | 0.13 | 0.16 | 0.04 |

Table S6b. Impact of data sampling frequency on absolute error (walking noise data set).

| Shape | Method | (3.0) 0.1 | (3.0) 1.0 | (5.0) 0.1 | (5.0) 1.0 | (5.0) 2.0 | (10) 2.0 |
| --- | --- | --- | --- | --- | --- | --- | --- |
| cos | EPR | 0.39 | 0.4 | 0.08 | 0.14 | 0.15 | 0.04 |
| cos | MFF | 0.27 | 0.28 | 0.11 | 0.11 | 0.11 | 0.04 |
| cos | NLLS | 0.29 | 0.28 | 0.12 | 0.12 | 0.11 | 0.04 |
| cos | MESA | 0.31 | 0.3 | 0.12 | 0.13 | 0.12 | 0.05 |
| cos | LSPR | 0.26 | 0.26 | 0.11 | 0.11 | 0.1 | 0.04 |
| cos | SR | 0.32 | 0.35 | 0.15 | 0.15 | 0.13 | 0.05 |
| pul | EPR | 0.33 | 0.38 | 0.07 | 0.13 | 0.13 | 0.03 |
| pul | MFF | 0.21 | 0.26 | 0.09 | 0.09 | 0.1 | 0.03 |
| pul | NLLS | 0.27 | 0.31 | 0.13 | 0.12 | 0.12 | 0.04 |
| pul | MESA | 0.28 | 0.29 | 0.14 | 0.14 | 0.13 | 0.06 |
| pul | LSPR | 0.27 | 0.28 | 0.12 | 0.11 | 0.11 | 0.04 |
| pul | SR | 0.32 | 0.34 | 0.18 | 0.16 | 0.15 | 0.04 |
| dblp | EPR | 0.18 | 0.2 | 0.03 | 0.07 | 0.07 | 0.01 |
| dblp | MFF | 0.19 | 0.19 | 0.07 | 0.07 | 0.08 | 0.03 |
| dblp | NLLS | 0.39 | 0.36 | 0.14 | 0.13 | 0.14 | 0.04 |
| dblp | MESA | 0.38 | 0.37 | 0.16 | 0.15 | 0.15 | 0.07 |
| dblp | LSPR | 0.49 | 0.4 | 0.16 | 0.16 | 0.15 | 0.05 |
| dblp | SR | 0.63 | 0.64 | 0.23 | 0.22 | 0.21 | 0.05 |
| shl | EPR | 0.42 | 0.35 | 0.11 | 0.12 | 0.12 | 0.02 |
| shl | MFF | 0.23 | 0.21 | 0.09 | 0.09 | 0.09 | 0.04 |
| shl | NLLS | 0.34 | 0.34 | 0.15 | 0.13 | 0.14 | 0.04 |
| shl | MESA | 0.29 | 0.28 | 0.13 | 0.13 | 0.13 | 0.05 |
| shl | LSPR | 0.37 | 0.34 | 0.14 | 0.14 | 0.14 | 0.04 |
| shl | SR | 0.55 | 0.46 | 0.17 | 0.16 | 0.17 | 0.05 |
| asym | EPR | 0.54 | 0.53 | 0.1 | 0.07 | 0.07 | 0.04 |
| asym | MFF | 0.27 | 0.24 | 0.07 | 0.09 | 0.11 | 0.04 |
| asym | NLLS | 0.38 | 0.36 | 0.14 | 0.14 | 0.13 | 0.05 |
| asym | MESA | 0.33 | 0.32 | 0.14 | 0.14 | 0.14 | 0.07 |
| asym | LSPR | 0.61 | 0.53 | 0.21 | 0.21 | 0.18 | 0.06 |
| asym | SR | 0.56 | 0.57 | 0.18 | 0.18 | 0.17 | 0.06 |
| all | EPR | 0.37 | 0.37 | 0.08 | 0.11 | 0.11 | 0.03 |
| all | MFF | 0.23 | 0.23 | 0.09 | 0.09 | 0.1 | 0.03 |
| all | NLLS | 0.33 | 0.33 | 0.14 | 0.13 | 0.13 | 0.04 |
| all | MESA | 0.32 | 0.31 | 0.14 | 0.14 | 0.13 | 0.06 |
| all | LSPR | 0.4 | 0.36 | 0.15 | 0.14 | 0.14 | 0.05 |
| all | SR | 0.48 | 0.47 | 0.18 | 0.17 | 0.17 | 0.05 |

Data sets with different time interval and selected durations were analysed using all the methods and the average absolute error is reported in the table, the absolute error is defined as the absolute value of the difference between calculated period and the expected value (24.08 for asym signal and 24h for the others). Data sets were created by adding 80 % noise to the templates of different duration and time interval between points. 1) The base shape of the signal: cosine (cos), pulse (pul); double pulse (dpl); shoulder (shl) and moderate asymmetry (asym), (all) represents aggregated results from all the sets. 2) the time interval (sampling frequency) in the data set and in brackets the data duration in days.
