# Supplementary material for: Strengths and Limitations of Period Estimation Methods for Circadian Data
Source: PLoS One. 2014 May 8;9(5):e96462. doi: 10.1371/journal.pone.0096462 (PMC4014635; doi:10.1371/journal.pone.0096462)
Supplement: Table S7 — Impact of baseline trends on mean period. (DOCX) [file pone.0096462.s014.docx]

Table S7. Impact of baseline trend level on periods estimates.

| Trend | Method | Mextric | 0 | 1 | 5 | 10 | 20 | 100 |
| --- | --- | --- | --- | --- | --- | --- | --- | --- |
| exp | NLLS | MP | 23.95 | 23.92 | 23.66 | 612.09 | 705.89 | 682.72 |
| exp | LSPR | MP | 24.01 | 23.92 | 23.45 | 22.88 | 35.63 | 35.74 |
| exp | MESA | MP | 23.92 | 23.92 | 23.92 | 23.9 | 23.88 | 23.88 |
| exp | MFF | MP | 23.98 | 23.94 | 23.38 | 22.95 | 31.87 | 35.01 |
| exp | EPR | MP | 23.88 | 23.87 | 23.65 | 23.05 | 35.1 | 35.1 |
| exp | SR | MP | 24.11 | 24.11 | 24.1 | 24.1 | 24.09 | 24.01 |
| exp | NLLS | STD | 0.15 | 0.14 | 0.28 | 657 | 227.48 | 52.87 |
| exp | LSPR | STD | 0.13 | 0.13 | 0.14 | 0.09 | 1.66 | 0.02 |
| exp | MESA | STD | 0.15 | 0.15 | 0.15 | 0.16 | 0.17 | 0.17 |
| exp | MFF | STD | 0.11 | 0.13 | 0.1 | 0.08 | 5.31 | 0.01 |
| exp | EPR | STD | 0.11 | 0.11 | 0.26 | 1.16 | 0 | 0 |
| exp | SR | STD | 0.15 | 0.15 | 0.15 | 0.15 | 0.15 | 0.16 |
| ipar | NLLS | MP | 23.95 | 24.15 | 269.56 | 280.77 | 173.73 | 34.9 |
| ipar | LSPR | MP | 24.01 | 24.31 | 34.88 | 35.3 | 35.31 | 35.32 |
| ipar | MESA | MP | 23.92 | 23.98 | 23.98 | 24.19 | 24.69 | 34.67 |
| ipar | MFF | MP | 23.98 | 23.76 | 34.62 | 34.9 | 34.85 | 34.82 |
| ipar | EPR | MP | 23.88 | 23.8 | 34.61 | 34.62 | 34.6 | 34.6 |
| ipar | SR | MP | 24.11 | 24.1 | 24.07 | 29.57 | 46.88 | 46.75 |
| ipar | NLLS | STD | 0.15 | 0.25 | 77.77 | 23.87 | 120.04 | 6.57 |
| ipar | LSPR | STD | 0.13 | 0.2 | 1.77 | 0.04 | 0.02 | 0 |
| ipar | MESA | STD | 0.15 | 0.15 | 0.18 | 0.22 | 0.54 | 1.95 |
| ipar | MFF | STD | 0.11 | 0.04 | 1.65 | 0.03 | 0.02 | 0.01 |
| ipar | EPR | STD | 0.11 | 0.17 | 0.73 | 0.09 | 0.05 | 0 |
| ipar | SR | STD | 0.15 | 0.15 | 0.15 | 6.6 | 0.5 | 0.13 |
| lin | NLLS | MP | 23.95 | 23.96 | 24 | 24.05 | 24.14 | 24.74 |
| lin | LSPR | MP | 24.01 | 24.02 | 24.07 | 24.12 | 24.23 | 24.98 |
| lin | MESA | MP | 23.92 | 23.93 | 23.97 | 24.01 | 24.08 | 24.52 |
| lin | MFF | MP | 23.98 | 23.99 | 24.02 | 24.06 | 24.15 | 24.73 |
| lin | EPR | MP | 23.88 | 23.89 | 23.92 | 23.95 | 24.03 | 24.63 |
| lin | SR | MP | 24.11 | 24.12 | 24.14 | 24.17 | 24.22 | 40.67 |
| lin | NLLS | STD | 0.15 | 0.14 | 0.14 | 0.15 | 0.15 | 0.14 |
| lin | LSPR | STD | 0.13 | 0.13 | 0.13 | 0.13 | 0.13 | 0.11 |
| lin | MESA | STD | 0.15 | 0.15 | 0.15 | 0.15 | 0.15 | 0.25 |
| lin | MFF | STD | 0.11 | 0.11 | 0.11 | 0.1 | 0.1 | 0.06 |
| lin | EPR | STD | 0.11 | 0.1 | 0.1 | 0.1 | 0.1 | 0.12 |
| lin | SR | STD | 0.16 | 0.15 | 0.15 | 0.15 | 0.14 | 10.82 |
| 1/3par | NLLS | MP | 23.95 | 23.99 | 24.12 | 115.73 | 127.41 | 130.92 |
| 1/3par | LSPR | MP | 24.01 | 23.99 | 23.87 | 23.66 | 34.92 | 35.15 |
| 1/3par | MESA | MP | 23.92 | 23.98 | 24.02 | 23.98 | 23.94 | 24.21 |
| 1/3par | MFF | MP | 23.98 | 24 | 23.69 | 23.4 | 32.1 | 34.49 |
| 1/3par | EPR | MP | 23.88 | 23.9 | 24.05 | 24.51 | 33.59 | 34.22 |
| 1/3par | SR | MP | 24.11 | 24.11 | 24.13 | 24.14 | 24.26 | 76.06 |
| 1/3par | NLLS | STD | 0.15 | 0.15 | 0.12 | 30.91 | 5.16 | 1.18 |
| 1/3par | LSPR | STD | 0.13 | 0.13 | 0.13 | 0.15 | 1.06 | 0.02 |
| 1/3par | MESA | STD | 0.15 | 0.15 | 0.15 | 0.16 | 0.17 | 0.25 |
| 1/3par | MFF | STD | 0.11 | 0.11 | 0.32 | 0.11 | 4.45 | 0.02 |
| 1/3par | EPR | STD | 0.11 | 0.11 | 0.26 | 0.23 | 0.43 | 0.08 |
| 1/3par | SR | STD | 0.15 | 0.15 | 0.16 | 0.16 | 0.73 | 0.58 |
| 2/3ipar | NLLS | MP | 23.95 | 24.03 | 344.52 | 380.95 | 289.22 | 320.2 |
| 2/3ipar | LSPR | MP | 24.01 | 24.15 | 25.18 | 34.49 | 35.53 | 35.58 |
| 2/3ipar | MESA | MP | 23.92 | 23.98 | 23.98 | 23.92 | 23.91 | 24.09 |
| 2/3ipar | MFF | MP | 23.98 | 23.94 | 25.05 | 34.72 | 35.02 | 34.99 |
| 2/3ipar | EPR | MP | 23.88 | 23.87 | 24.9 | 34.54 | 34.81 | 34.9 |
| 2/3ipar | NLLS | STD | 0.15 | 0.13 | 529.38 | 435.3 | 120.98 | 66.86 |
| 2/3ipar | LSPR | STD | 0.13 | 0.14 | 0.45 | 2.75 | 0.05 | 0.01 |
| 2/3ipar | MESA | STD | 0.15 | 0.15 | 0.16 | 0.17 | 0.18 | 0.2 |
| 2/3ipar | MFF | STD | 0.11 | 0.15 | 0.8 | 1.51 | 0 | 0.01 |
| 2/3ipar | EPR | STD | 0.11 | 0.1 | 1.31 | 1.49 | 0.09 | 0.02 |
| 2/3ipar | SR | STD | 0.15 | 0.15 | 0.15 | 0.15 | 0.15 | 18.14 |

Data sets with different levels of baseline trend and different trend forms were analysed using all the methods and the mean period value (MP) and standard deviation (STD) are reported in the table. Data sets were created by taking a standard pulse signal data set (5 days data, hourly sampled, 80% walking noise level, 24h underlying period) and adding to it 5 different envelope shapes with increasing amplitude. 1) The trend/envelope shapes: linear increase (lin); exponential increase (exp); inverse parabola (ipar); 2/3 inverse parabola (2/3ipar) and 1/3 parabola (1/3par). 2) The baseline level is defined as ration between trend total amplitude and the original signal amplitude (0 no trend, 20 trend is 20 times higher than signal).
