# Supplementary material for: Strengths and Limitations of Period Estimation Methods for Circadian Data
Source: PLoS One. 2014 May 8;9(5):e96462. doi: 10.1371/journal.pone.0096462 (PMC4014635; doi:10.1371/journal.pone.0096462)
Supplement: Table S8 — Impact of amplitude trends on mean period. (DOCX) [file pone.0096462.s015.docx]

Table S8. Impact of amplitude dampening on periods estimates.

| Trend | Method | Metric | 0 | 0.1 | 0.2 | 0.4 | 0.6 | 0.8 | 0.9 | 1 |
| --- | --- | --- | --- | --- | --- | --- | --- | --- | --- | --- |
| exp | NLLS | MP | 23.95 | 23.95 | 23.96 | 23.96 | 23.97 | 23.98 | 24.01 | 24.01 |
| exp | LSPR | MP | 24.01 | 24.01 | 24.01 | 24 | 24 | 23.96 | 23.92 | 23.81 |
| exp | MESA | MP | 23.92 | 23.93 | 23.94 | 23.95 | 23.97 | 23.98 | 23.98 | 23.95 |
| exp | MFF | MP | 23.98 | 23.98 | 23.98 | 23.98 | 23.98 | 23.95 | 23.91 | 23.78 |
| exp | EPR | MP | 23.88 | 23.89 | 23.89 | 23.89 | 23.89 | 23.88 | 23.85 | 23.76 |
| exp | SR | MP | 24.11 | 24.12 | 24.12 | 24.14 | 24.17 | 24.23 | 24.32 | 24.43 |
| exp | NLLS | STD | 0.15 | 0.15 | 0.14 | 0.14 | 0.14 | 0.14 | 0.16 | 0.18 |
| exp | LSPR | STD | 0.13 | 0.13 | 0.13 | 0.13 | 0.13 | 0.14 | 0.15 | 0.19 |
| exp | MESA | STD | 0.15 | 0.15 | 0.15 | 0.16 | 0.2 | 0.29 | 0.38 | 0.48 |
| exp | MFF | STD | 0.11 | 0.11 | 0.11 | 0.11 | 0.12 | 0.15 | 0.18 | 0.25 |
| exp | EPR | STD | 0.11 | 0.11 | 0.1 | 0.1 | 0.11 | 0.12 | 0.13 | 0.15 |
| exp | SR | STD | 0.15 | 0.15 | 0.15 | 0.15 | 0.15 | 0.18 | 0.22 | 0.3 |
| lin | NLLS | MP | 23.95 | 23.95 | 23.95 | 23.95 | 23.95 | 23.94 | 23.94 | 23.92 |
| lin | LSPR | MP | 24.01 | 24.01 | 24.01 | 24 | 23.99 | 23.96 | 23.94 | 23.91 |
| lin | MESA | MP | 23.92 | 23.92 | 23.92 | 23.92 | 23.9 | 23.87 | 23.85 | 23.81 |
| lin | MFF | MP | 23.98 | 23.98 | 23.97 | 23.97 | 23.96 | 23.93 | 23.91 | 23.87 |
| lin | EPR | MP | 23.88 | 23.88 | 23.88 | 23.88 | 23.87 | 23.84 | 23.82 | 23.79 |
| lin | SR | MP | 24.11 | 24.11 | 24.12 | 24.13 | 24.15 | 24.17 | 24.21 | 24.25 |
| lin | NLLS | STD | 0.15 | 0.15 | 0.14 | 0.14 | 0.14 | 0.14 | 0.15 | 0.17 |
| lin | LSPR | STD | 0.13 | 0.13 | 0.13 | 0.13 | 0.13 | 0.13 | 0.14 | 0.15 |
| lin | MESA | STD | 0.15 | 0.15 | 0.15 | 0.16 | 0.18 | 0.21 | 0.25 | 0.29 |
| lin | MFF | STD | 0.11 | 0.11 | 0.11 | 0.11 | 0.12 | 0.13 | 0.14 | 0.17 |
| lin | EPR | STD | 0.11 | 0.11 | 0.11 | 0.11 | 0.11 | 0.11 | 0.11 | 0.12 |
| lin | SR | STD | 0.15 | 0.15 | 0.15 | 0.15 | 0.15 | 0.17 | 0.19 | 0.21 |
| 1/3par | NLLS | MP | 23.95 | 23.95 | 23.95 | 23.95 | 23.94 | 23.93 | 23.92 | 23.9 |
| 1/3par | LSPR | MP | 24.01 | 24.01 | 24 | 23.99 | 23.98 | 23.96 | 23.94 | 23.91 |
| 1/3par | MESA | MP | 23.92 | 23.92 | 23.91 | 23.88 | 23.84 | 23.77 | 23.72 | 23.67 |
| 1/3par | MFF | MP | 23.98 | 23.97 | 23.97 | 23.96 | 23.94 | 23.9 | 23.89 | 23.85 |
| 1/3par | EPR | MP | 23.88 | 23.87 | 23.87 | 23.86 | 23.84 | 23.81 | 23.79 | 23.76 |
| 1/3par | SR | MP | 24.11 | 24.12 | 24.12 | 24.12 | 24.14 | 24.16 | 24.17 | 24.19 |
| 1/3par | NLLS | STD | 0.15 | 0.15 | 0.14 | 0.14 | 0.14 | 0.14 | 0.15 | 0.16 |
| 1/3par | LSPR | STD | 0.13 | 0.13 | 0.13 | 0.13 | 0.13 | 0.13 | 0.14 | 0.15 |
| 1/3par | MESA | STD | 0.15 | 0.15 | 0.15 | 0.16 | 0.18 | 0.22 | 0.26 | 0.3 |
| 1/3par | MFF | STD | 0.11 | 0.11 | 0.11 | 0.11 | 0.11 | 0.14 | 0.14 | 0.16 |
| 1/3par | EPR | STD | 0.11 | 0.11 | 0.1 | 0.1 | 0.1 | 0.1 | 0.11 | 0.11 |
| 1/3par | SR | STD | 0.16 | 0.15 | 0.15 | 0.15 | 0.15 | 0.17 | 0.18 | 0.2 |
| 2/3par | NLLS | MP | 23.95 | 23.96 | 23.96 | 23.97 | 23.98 | 24.01 | 24.04 | 23.99 |
| 2/3par | LSPR | MP | 24.01 | 24.01 | 24.01 | 24 | 23.98 | 23.92 | 23.81 | 23.65 |
| 2/3par | MESA | MP | 23.92 | 23.93 | 23.94 | 23.95 | 23.98 | 23.96 | 23.91 | 23.81 |
| 2/3par | MFF | MP | 23.98 | 23.98 | 23.98 | 23.98 | 23.97 | 23.91 | 23.78 | 23.36 |
| 2/3par | EPR | MP | 23.88 | 23.89 | 23.89 | 23.9 | 23.9 | 23.88 | 23.82 | 23.6 |
| 2/3par | SR | MP | 24.11 | 24.12 | 24.13 | 24.15 | 24.19 | 24.31 | 24.47 | 24.85 |
| 2/3par | NLLS | STD | 0.15 | 0.14 | 0.14 | 0.14 | 0.15 | 0.15 | 0.17 | 1 |
| 2/3par | LSPR | STD | 0.13 | 0.13 | 0.13 | 0.13 | 0.14 | 0.15 | 0.17 | 1.05 |
| 2/3par | MESA | STD | 0.15 | 0.15 | 0.15 | 0.17 | 0.23 | 0.4 | 0.5 | 0.63 |
| 2/3par | MFF | STD | 0.11 | 0.11 | 0.11 | 0.12 | 0.13 | 0.19 | 0.26 | 0.65 |
| 2/3par | EPR | STD | 0.11 | 0.11 | 0.1 | 0.1 | 0.11 | 0.14 | 0.18 | 0.44 |
| 2/3par | SR | STD | 0.15 | 0.15 | 0.15 | 0.15 | 0.16 | 0.22 | 0.31 | 0.58 |
| par | NLLS | MP | 23.95 | 23.96 | 23.97 | 24 | 24.03 | 24.09 | 24.1 | 24.37 |
| par | LSPR | MP | 24.01 | 24.01 | 24.01 | 24.01 | 24.01 | 24.01 | 24 | 23.99 |
| par | MESA | MP | 23.92 | 23.94 | 23.96 | 23.99 | 23.99 | 23.74 | 23.38 | 22.64 |
| par | MFF | MP | 23.98 | 23.98 | 23.99 | 24.01 | 24.02 | 23.98 | 23.97 | 24.23 |
| par | EPR | MP | 23.88 | 23.89 | 23.91 | 23.94 | 23.97 | 24.04 | 24.08 | 24.34 |
| par | SR | MP | 24.11 | 24.12 | 24.13 | 24.15 | 24.19 | 24.22 | 24.25 | 25.23 |
| par | NLLS | STD | 0.15 | 0.15 | 0.15 | 0.16 | 0.17 | 0.16 | 0.2 | 2.49 |
| par | LSPR | STD | 0.13 | 0.13 | 0.13 | 0.14 | 0.15 | 0.16 | 0.18 | 0.2 |
| par | MESA | STD | 0.15 | 0.15 | 0.16 | 0.19 | 0.3 | 0.46 | 0.57 | 0.7 |
| par | MFF | STD | 0.11 | 0.11 | 0.11 | 0.12 | 0.13 | 0.19 | 0.23 | 1.75 |
| par | EPR | STD | 0.11 | 0.1 | 0.11 | 0.11 | 0.13 | 0.15 | 0.17 | 1.28 |
| par | SR | STD | 0.15 | 0.15 | 0.15 | 0.15 | 0.16 | 0.17 | 0.18 | 1.64 |

Data sets with different levels of amplitude trend and different trend forms were analysed using all the methods and the mean period (MP) and standard deviation (STD) are reported in the table. The amplitude trends were obtained by dampening the test data sets to the stated level at the last day using different trend shapes/envelopes. Dampening was applied to a standard pulse data set (5 days data, hourly sampled, 80% walking noise level, 24h underlying period) and adding to it 5 different envelope shapes with increasing amplitude. 1) The trend/envelope shapes: linear decrease (lin); exponential decrease; parabola (par); 2/3 parabola (2/3par) and 1/3 parabola (1/3par) 2) The level of amplitude trend, ie, the maximal level of original signal deduction. 0 means no dampening, and for example 0.6 for lin. trend means that at the end of 5^th^ day, the signal is reduced to 40% of its original vale.
