# Supplementary material for: Strengths and Limitations of Period Estimation Methods for Circadian Data
Source: PLoS One. 2014 May 8;9(5):e96462. doi: 10.1371/journal.pone.0096462 (PMC4014635; doi:10.1371/journal.pone.0096462)
Supplement: Table S9 — Analysis of white noise signal. (DOCX) [file pone.0096462.s016.docx]

Table S10. Ratio of different results types when uniform noise was analysed.

| Method | NLLS | LSPR | MESA | MFF | EPR | SR |
| --- | --- | --- | --- | --- | --- | --- |
| N | 100 | 100 | 100 | 100 | 100 | 100 |
| Arrhythmic | 21 | 99 | 0 | 0 | 6 | 0 |
| Circadian (False positives) | 2 | 0 | 62 | 78 | 32 | 0 |
| Non-Circadian | 77 | 1 | 38 | 22 | 62 | 100 |

The results of 100 replicates of analysis of uniform noise were classified as: arrhythmic (if were rejected by method), circadian if the period found was in the range 16-32 hours (treated as false positives), and non-circadian for any other period value.
