# Supplementary material for: Strengths and Limitations of Period Estimation Methods for Circadian Data
Source: PLoS One. 2014 May 8;9(5):e96462. doi: 10.1371/journal.pone.0096462 (PMC4014635; doi:10.1371/journal.pone.0096462)
Supplement: Table S10 — Analysis of strongly dampened signals for arhythmicity test. (DOCX) [file pone.0096462.s017.docx]

Table S11a. Ratio of different results types when dampened pulse signal was analysed.

| False positive | | Pulse | Noise 30% | |  |  |  |  |
| --- | --- | --- | --- | --- | --- | --- | --- | --- |
| DMP | 1 | 1.5 | 2 | 2.5 | 3 | 4 | 5 | 10 |
| NLLS | 2 | 89 | 95 | 93 | 84 | 17 | 0 | 0 |
| LSPR | 12 | 99 | 100 | 98 | 91 | 13 | 0 | 0 |
| MESA | 77 | 55 | 32 | 26 | 9 | 0 | 0 | 0 |
| MFF | 9 | 92 | 99 | 96 | 90 | 24 | 0 | 0 |
| EPR | 0 | 1 | 56 | 90 | 78 | 4 | 0 | 0 |
| SR | 0 | 78 | 99 | 93 | 79 | 7 | 0 | 0 |
| In range |  | Pulse | Noise 30% | |  |  |  |  |
| DMP | 1 | 1.5 | 2 | 2.5 | 3 | 4 | 5 | 10 |
| NLLS | 0 | 7 | 5 | 7 | 16 | 83 | 100 | 100 |
| LSPR | 0 | 0 | 0 | 2 | 9 | 87 | 100 | 100 |
| MESA | 15 | 45 | 68 | 74 | 91 | 100 | 100 | 100 |
| MFF | 0 | 0 | 1 | 4 | 10 | 76 | 100 | 100 |
| EPR | 0 | 0 | 2 | 10 | 22 | 96 | 100 | 100 |
| SR | 0 | 20 | 1 | 7 | 21 | 93 | 100 | 100 |
| Arrythmic | | Pulse | Noise 30% | |  |  |  |  |
| DMP | 1 | 1.5 | 2 | 2.5 | 3 | 4 | 5 | 10 |
| NLLS | 0 | 0 | 0 | 0 | 0 | 0 | 0 | 0 |
| LSPR | 14 | 0 | 0 | 0 | 0 | 0 | 0 | 0 |
| MESA | 0 | 0 | 0 | 0 | 0 | 0 | 0 | 0 |
| MFF | 0 | 0 | 0 | 0 | 0 | 0 | 0 | 0 |
| EPR | 100 | 99 | 42 | 0 | 0 | 0 | 0 | 0 |
| SR | 0 | 0 | 0 | 0 | 0 | 0 | 0 | 0 |
| False positive | | Pulse | Noise 80% | |  |  |  |  |
| DMP | 1 | 1.5 | 2 | 2.5 | 3 | 4 | 5 | 10 |
| NLLS | 22 | 70 | 86 | 82 | 83 | 50 | 3 | 0 |
| LSPR | 18 | 64 | 87 | 90 | 86 | 55 | 4 | 0 |
| MESA | 58 | 72 | 55 | 44 | 34 | 10 | 2 | 0 |
| MFF | 48 | 79 | 91 | 91 | 84 | 58 | 7 | 0 |
| EPR | 8 | 28 | 64 | 80 | 79 | 50 | 8 | 0 |
| SR | 8 | 70 | 88 | 71 | 66 | 30 | 7 | 0 |
| In range |  | Pulse | Noise 80% | |  |  |  |  |
| DMP | 1 | 1.5 | 2 | 2.5 | 3 | 4 | 5 | 10 |
| NLLS | 1 | 6 | 10 | 18 | 17 | 50 | 97 | 100 |
| LSPR | 1 | 5 | 9 | 10 | 14 | 45 | 96 | 100 |
| MESA | 7 | 27 | 45 | 56 | 66 | 90 | 98 | 100 |
| MFF | 1 | 4 | 4 | 9 | 16 | 42 | 93 | 100 |
| EPR | 0 | 3 | 5 | 17 | 21 | 50 | 92 | 100 |
| SR | 3 | 9 | 10 | 29 | 34 | 70 | 93 | 100 |
| Arrythmic | | Pulse | Noise 80% | |  |  |  |  |
| DMP | 1 | 1.5 | 2 | 2.5 | 3 | 4 | 5 | 10 |
| NLLS | 0 | 0 | 0 | 0 | 0 | 0 | 0 | 0 |
| LSPR | 59 | 23 | 2 | 0 | 0 | 0 | 0 | 0 |
| MESA | 0 | 0 | 0 | 0 | 0 | 0 | 0 | 0 |
| MFF | 0 | 0 | 0 | 0 | 0 | 0 | 0 | 0 |
| EPR | 91 | 69 | 30 | 3 | 0 | 0 | 0 | 0 |
| SR | 0 | 0 | 0 | 0 | 0 | 0 | 0 | 0 |

The results of 100 replicates of analysis of dampened data, which were classified as: arrhythmic (if were rejected by method), in range if the period found was in the range 24h+-0.5 hours, and false positive if the period was circadian (18-30h) but not in the range of 24h+-0.5h, table presents numbers for each category and each method.

Table S11b. Ratio of different results types when dampened double pulse signal was analysed.

| False positive | | Double pulse | | Noise 30% | |  |  |  |
| --- | --- | --- | --- | --- | --- | --- | --- | --- |
| DMP | 1 | 1.5 | 2 | 2.5 | 3 | 4 | 5 | 10 |
| NLLS | 0 | 29 | 95 | 94 | 94 | 70 | 0 | 0 |
| LSPR | 2 | 52 | 100 | 100 | 100 | 60 | 0 | 0 |
| MESA | 70 | 56 | 30 | 22 | 3 | 0 | 0 | 0 |
| MFF | 17 | 89 | 98 | 91 | 76 | 4 | 0 | 0 |
| EPR | 0 | 0 | 31 | 84 | 76 | 6 | 0 | 0 |
| SR | 0 | 76 | 99 | 88 | 74 | 17 | 0 | 0 |
| In range |  | Double pulse | | Noise 30% | |  |  |  |
| DMP | 1 | 1.5 | 2 | 2.5 | 3 | 4 | 5 | 10 |
| NLLS | 0 | 1 | 5 | 6 | 6 | 30 | 100 | 100 |
| LSPR | 0 | 0 | 0 | 0 | 0 | 40 | 100 | 100 |
| MESA | 13 | 44 | 70 | 78 | 97 | 100 | 100 | 100 |
| MFF | 1 | 0 | 0 | 9 | 24 | 96 | 100 | 100 |
| EPR | 0 | 0 | 0 | 16 | 24 | 94 | 100 | 100 |
| SR | 0 | 19 | 1 | 12 | 26 | 83 | 100 | 100 |
| Arrythmic | | Double pulse | | Noise 30% | |  |  |  |
| DMP | 1 | 1.5 | 2 | 2.5 | 3 | 4 | 5 | 10 |
| NLLS | 0 | 0 | 0 | 0 | 0 | 0 | 0 | 0 |
| LSPR | 33 | 38 | 0 | 0 | 0 | 0 | 0 | 0 |
| MESA | 0 | 0 | 0 | 0 | 0 | 0 | 0 | 0 |
| MFF | 0 | 0 | 0 | 0 | 0 | 0 | 0 | 0 |
| EPR | 100 | 100 | 69 | 0 | 0 | 0 | 0 | 0 |
| SR | 0 | 0 | 0 | 0 | 0 | 0 | 0 | 0 |
| False positive | | Double pulse | | Noise 80% | |  |  |  |
| DMP | 1 | 1.5 | 2 | 2.5 | 3 | 4 | 5 | 10 |
| NLLS | 17 | 48 | 82 | 85 | 85 | 79 | 26 | 0 |
| LSPR | 8 | 25 | 69 | 90 | 94 | 77 | 21 | 0 |
| MESA | 52 | 83 | 65 | 55 | 39 | 18 | 8 | 0 |
| MFF | 51 | 76 | 87 | 88 | 84 | 41 | 9 | 0 |
| EPR | 6 | 16 | 43 | 69 | 78 | 44 | 7 | 0 |
| SR | 16 | 44 | 87 | 75 | 68 | 37 | 13 | 1 |
| In range |  | Double pulse | | Noise 80% | |  |  |  |
| DMP | 1 | 1.5 | 2 | 2.5 | 3 | 4 | 5 | 10 |
| NLLS | 0 | 1 | 7 | 13 | 15 | 21 | 74 | 100 |
| LSPR | 0 | 0 | 1 | 4 | 6 | 23 | 79 | 100 |
| MESA | 6 | 13 | 35 | 45 | 61 | 82 | 92 | 100 |
| MFF | 3 | 6 | 5 | 11 | 16 | 59 | 91 | 100 |
| EPR | 0 | 3 | 4 | 14 | 22 | 56 | 93 | 100 |
| SR | 2 | 9 | 6 | 25 | 32 | 63 | 87 | 99 |
| Arrythmic | | Double pulse | | Noise 80% | |  |  |  |
| DMP | 1 | 1.5 | 2 | 2.5 | 3 | 4 | 5 | 10 |
| NLLS | 0 | 0 | 0 | 0 | 0 | 0 | 0 | 0 |
| LSPR | 76 | 68 | 26 | 6 | 0 | 0 | 0 | 0 |
| MESA | 0 | 0 | 0 | 0 | 0 | 0 | 0 | 0 |
| MFF | 0 | 0 | 0 | 0 | 0 | 0 | 0 | 0 |
| EPR | 93 | 80 | 49 | 14 | 0 | 0 | 0 | 0 |
| SR | 0 | 0 | 0 | 0 | 0 | 0 | 0 | 0 |

The results of 100 replicates of analysis of dampened data, which were classified as: arrhythmic (if were rejected by method), in range if the period found was in the range 24h+-0.5 hours, and false positive if the period was circadian (18-30h) but not in the range of 24h+-0.5h, table presents numbers for each category and each method.
