# Supplementary material for: Strengths and Limitations of Period Estimation Methods for Circadian Data
Source: PLoS One. 2014 May 8;9(5):e96462. doi: 10.1371/journal.pone.0096462 (PMC4014635; doi:10.1371/journal.pone.0096462)
Supplement: Doc S1 — Modification of EPR algorithm. (DOCX) [file pone.0096462.s018.docx]

Modification of Enright Periodogram algorithm.

In his original paper Enright used hourly sampled data to construct the “Buys Ballot” table, based on which ‘the spectrum value’ for particular period was calculated. It was straight forward for integer period value as the number of table columns matched the period value and each data point could be inserted into the table by walking from left to the right and top to the bottom. However to construct such table for fractional period (for example 24.5) another approach was necessary. The table could not obviously have 24 and half columns, hence it had only 24 columns, but the table was populated “by advancing through the data at an average rate of 24.5 hours”. For 24.5 hour period, it lead to skipping data point at time 49 (2*24.5) and using the next one instead (numbered 50).

However, this method is limited only to long data. For example to calculate the spectrum value for period 24.1, the first point which will be skipped has index 241, so it can only happen after 10 days of measurements. For example when analysing the 5 day data, “buys ballot” tables for the test periods 24h and 24.1h looks exactly the same, and the method cannot distinguish between those periods.

In our approach, the data are always interpolated to provide time interval 0.1 hour, the periods are also always scanned with 0.1 hour resolution, and the buys ballot table is constructed accordingly. Thus, the table for 24 hour period has 240 columns, and the one for 24.1 has 241, also each data point (although artificially generated) is used to populate the tables. Even for the short data (3 days) the “buys ballot” tables for 24h and 24.1h periods looks different and the method, in principle, can distinguish between them.

To illustrate it, we generated times series of periods between 24h and 25h with 0.1h step and fixed duration of 5 days. We analysed those data with ‘classic’ EPR implementation and the interpolating one. For the test data with underlying period in the range 24-24.3h the classic version reported the same period value (Figure A left), while the interpolating version was able to distinguish between them (however the calculated periods values are smaller than the expected ones). As explained above, for data lasting 5 days, classic method performed exactly the same calculations for the periods between 24 and 24.3h. When duration of the test data was longer than 10 days, the classic method could correctly estimate periods with 0.1h resolutions (Figures A right).

|  |
| --- |
| Figure A. Calculated period values vs expected period values, for ‘classic’ implementation of EPR and the ‘interpolating’, results for time series of 120 (left) and 240 (right) hours of duration |
