# Supplementary material for: Strengths and Limitations of Period Estimation Methods for Circadian Data
Source: PLoS One. 2014 May 8;9(5):e96462. doi: 10.1371/journal.pone.0096462 (PMC4014635; doi:10.1371/journal.pone.0096462)
Supplement: Doc S2 — NLLS computation time. (DOCX) [file pone.0096462.s019.docx]

Figure B: Instability of computation time for FFT-NLLS. The examples show how different time series require different computation times (top) and furthermore the addition of one data point can have a dramatic impact on the computation time (bottom).
